# Diversified food fortification portfolios can enhance coverage of micronutrient-vulnerable populations in West Africa

**DOI:** 10.1038/s43016-026-01412-2

**Published:** 2026-09-02

**Authors:** Kevin Tang, Uchenna Agu, Soukeyna Mbodj, Melas Adoko, Emily Becher, Gabriel Battcock, Daniel Hernandez, Mohammed Osman, Vanessa Elias, Suresh Lakshminarayanan, Saskia De Pee, Mahamadou Tanimoune, Frances Knight

**Affiliations:** 1 Nutrition and Food Quality Service, United Nations World Food Programme, Rome, Italy; 2 Faculty of Epidemiology and Population Health, London School of Hygiene & Tropical Medicine, London, UK; 3 Western and Central Africa Regional Office, United Nations World Food Programme, Dakar, Senegal; 4 Friedman School of Nutrition Science and Policy, Tufts University, Boston, MA USA

**Keywords:** Nutrition, Health policy, Malnutrition

## Abstract

Food fortification is central to addressing micronutrient deficiencies in West Africa and elsewhere. Yet evidence remains limited on whether current staple food fortification vehicles equitably reach vulnerable populations and whether candidate vehicles warrant inclusion. We analysed household consumption and expenditure surveys from ten countries in West Africa to estimate vulnerability to inadequate intake of five micronutrients, assessing wheat flour and edible oil as current standard vehicles and rice, maize, millet and sorghum as candidates. Vulnerability to inadequate intake was high across all ten countries: on average, households were probably vulnerable to around three to four of the five priority micronutrients assessed (mean probability of inadequacy 0.56–0.73), with Mali and Niger the most affected. More than half the population was vulnerable to inadequate iron, zinc and folate, and vulnerability was consistently higher in poorer households. Wheat flour and edible oil showed limited reach among poorer populations and low consumption among those most in need. Rice showed high coverage and alignment with vulnerability profiles across countries, while millet and sorghum showed potential to reduce inadequacy in the Sahel. These findings support the expansion of the regional vehicle portfolio through prioritization aligned with vulnerability and vehicle access.

## Abstract (web summary)

While staple food fortification is a recognized strategy for addressing micronutrient inadequacy, there is limited localized evidence on whether currently fortified foods, as well as candidate staples for future fortification, reach at-risk populations. This study finds that wheat flour and edible oil have limited coverage, whereas rice, millet and sorghum show greater potential across West Africa.

## Main

Micronutrient deficiencies remain a major public health challenge across West Africa, a region having some of the world’s highest burdens^1,2^. Vitamin A and zinc deficiencies elevate child mortality risk through impaired immune function and infection susceptibility, while iron, folate and vitamin B_12_ deficiencies contribute to maternal mortality and adverse birth outcomes via anaemia^3^. National surveys report anaemia in more than 40% of women of reproductive age and vitamin A deficiency affecting 30–75% of children under five across countries in the region^1^. The persistence of micronutrient deficiencies partly reflects nutrient-inadequate diets shaped by food systems that provide limited access to nutrient-dense or fortified foods and rely heavily on staples with low micronutrient content^4^. Given their structural drivers, reducing these widespread deficiencies depends on government action to develop and scale population-level interventions capable of improving micronutrient intake across diverse dietary contexts.

Large-scale food fortification (LSFF), which involves adding micronutrients to widely consumed staple foods and condiments (fortification vehicles) during industrial processing, is a cost-effective, population-level intervention that, when effectively implemented, consistently improves micronutrient intake^5^. The Economic Community of West African States (ECOWAS) has set regional fortification standards for wheat flour, edible oil and salt^6^, with several West African countries adopting corresponding national legislation over the past two decades^7^. However, current LSFF programmes in West Africa face two constraints that together limit population-level impact: incomplete industry compliance and inequitable vehicle reach. First, compliance assessments in Senegal^8,9^ and Nigeria^9^ document substantial shortfalls against national fortification standards, indicating that nutrient content in fortified vehicles often diverges from regulatory specification. Second, fortifiable wheat flour and edible oil disproportionately reach urban and wealthier households according to modelled assessments of national programmes in Senegal^10^, Burkina Faso^11^ and Nigeria^12^, leaving those at greatest nutritional risk underserved.

Rice, maize, sorghum and millet are candidate vehicles outside current regional standards, requiring operational models distinct from the established wheat flour and edible oil pathway. Dedicated global fortification guidance for rice and maize flour exists^13,14^, and their fortification operates on two pathways. Imports can be fortified through mandatory checks at the border^15,16^, or locally milled vehicles depend on consolidating fragmented small- and medium-scale mills and anchoring demand through public procurement^17,18^. Countries including Senegal, Nigeria and Côte d’Ivoire have embedded rice fortification initiatives in broader national nutrition strategies, indicating growing political momentum behind the vehicle^19–21^. Sorghum and millet fortification remains at earlier stages, with no mandatory regional or national programmes currently in place, although early-stage research suggests viable technological pathways for fortifying these grains^22^.

Fortification policy in West Africa is structured across two hierarchical layers, in which regional standards recommend the parameters within which national policy is set^7^. Regional standards specify candidate vehicles, micronutrient parameters and a shared monitoring framework, enabling intraregional trade and compliance pathways across mills at scale. National governments select which vehicles to mandate, calibrate fortification levels to country-specific dietary patterns and vulnerability, and handle implementation and enforcement. West African countries share enough structural features (for example, overlapping dietary staples, common import sources, similar systems for locally produced grains) to justify a common regional layer, while cross-country variation in dietary patterns and vulnerability profiles means that regional evidence can also illuminate where country-specific adaptation is needed^15,23^. However, existing analyses are predominantly country specific, leaving regional standard setting without comparable regional-level evidence.

The effectiveness of a fortification programme depends on the interaction between population vulnerability to inadequate micronutrient intake and access to fortified foods^24^: where vulnerability is high but access to the vehicle is limited, complementary approaches are needed. For vehicles already included in regional standards, this requires quantifying whether the fortifiable forms of the vehicle reach vulnerable subgroups. For candidate vehicles outside standards, the fundamental question is whether fortification is warranted at all and, if so, to what extent given observed consumption patterns characterizing pre-existing demand for the staple.

This study applies this framework across ten West African countries to identify where fortification can most equitably improve micronutrient adequacy to provide an evidence base for strengthening regional fortification efforts. To do so, we first estimate the magnitude and distribution of vulnerability to inadequate intake of five priority micronutrients across West Africa. We then assess fortification vehicles included in the current regional standards (wheat flour, edible oil) by examining their reach and consumption, as well as other staple foods (rice, maize, millet, sorghum) as candidate fortification vehicles in the region, by examining current consumption patterns to identify opportunities for future programme development. Finally, we explore the public health potential of fortifying each staple food by quantifying its coverage among the vulnerable populations who would benefit.

## Results

### Vulnerability to inadequate micronutrient intake

Table 1 presents the national estimates of the overall mean probability of inadequacy (MPI) for this study’s five priority micronutrients and percentage vulnerable (%) to inadequate intake of each individual micronutrient. For the 10 study countries, the national overall MPI ranged from 0.56 to 0.73, and the countries with the highest overall MPI were Mali (0.73) and Niger (0.73). For each micronutrient at the national level, more than half of the population were vulnerable to inadequate intake of zinc (range: 64–85%), folate (range: 57–82%) and iron (range: 56–74%). The highest zinc vulnerability was observed in Togo (85%), while folate inadequacy was greatest in Guinea-Bissau (89%) and Senegal (82%), and iron inadequacy was highest in Guinea-Bissau (74%) and Mali (73%). By comparison, the percentage vulnerable to inadequate vitamin A (range: 46–94%) and vitamin B_12_ (range: 23–88%) intake showed greater variability between countries. The countries most vulnerable to inadequate vitamin A intake were Niger (94%), Burkina Faso (94%) and Mali (85%), and the countries that were most vulnerable to inadequate vitamin B_12_ intake were Niger (88%), Benin (64%) and Togo (58%).

**Table 1:** National-level vulnerability to inadequate micronutrient intake for ten countries in the West African region

Country	Overall MPI	Percentage vulnerable to inadequate intake				
		Vitamin A (%)	Folate (%)	Vitamin B_12_ (%)	Zinc (%)	Iron (%)
Benin	0.61	47	60	64	77	57
Burkina Faso	0.64	94	66	45	76	56
Côte d’Ivoire	0.67	74	80	39	79	65
Ghana	0.56	60	63	29	64	62
Guinea-Bissau	0.66	46	89	43	81	74
Mali	0.73	85	75	54	81	73
Niger	0.73	94	47	88	81	58
Nigeria	0.63	84	57	49	75	55
Senegal	0.63	63	82	23	81	72
Togo	0.69	58	73	58	85	69

Inadequate intake is measured as the MPI and the percentage of the population at risk for inadequate intake of vitamin A, folate, vitamin B_12_, zinc and iron^53^. Estimates are from the EHCVM (2021–2022), GLSS (2016–2017) and NLSS (2018–2019). The overall MPI is the arithmetic MPI values across all five micronutrients (probability approach); it is not equivalent to the mean of the percentage vulnerable values in adjacent columns, which use the cut-point approach (vitamin A, folate, vitamin B_12_, zinc)^74^ and full probability approach (iron)^73^. The cut-point threshold is the apparent intake below the harmonized average requirement for a non-pregnant, non-lactating woman aged 18–29 (ref. ^72^). Iron and zinc requirement distributions assume low bioavailability (unrefined-grain diet high in dietary phytate).

**Fig. 1:**
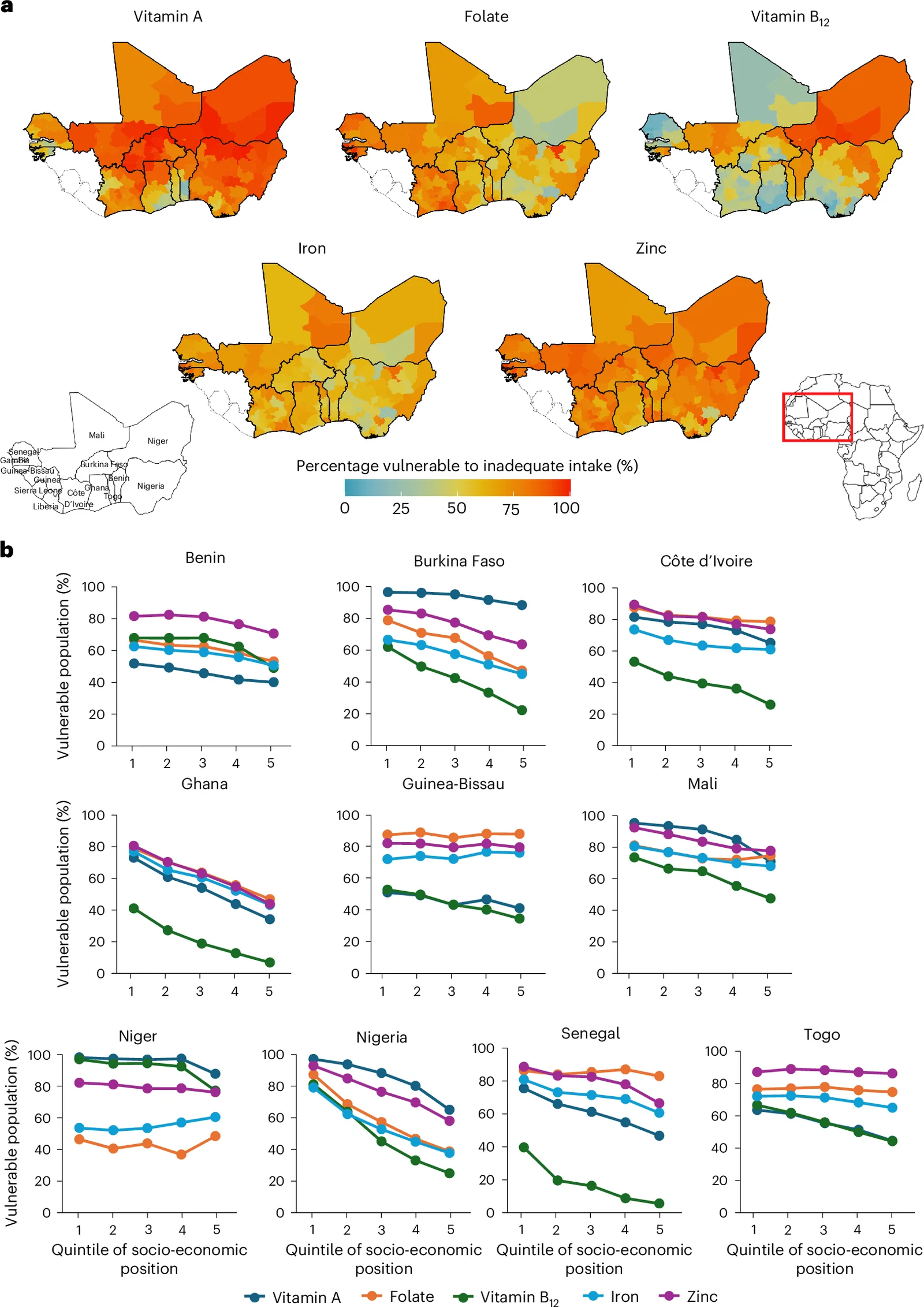
Percentage of the population vulnerable to inadequacy of vitamin A, folate, vitamin B_12_, iron and zinc for ten countries in the West African region. **a**,**b**, Percentages are shown by first administrative units (ADM1) (**a**) and quintile of socio-economic position (poorest = 1 to wealthiest = 5) (**b**). Percentages vulnerable to inadequate intake were estimated using the EHCVM (2021–2022), the GLSS (2016–2017) and the NLSS (2018–2019). The percentage vulnerable to inadequate intake was estimated using the cut-point approach^74^, in which apparent intakes lower than the harmonized average requirement of a non-pregnant, non-lactating 18–29-year-old woman^72^ were identified as vulnerable to inadequate intake. The percentage vulnerable to iron inadequacy was estimated using the full probability approach^73^. The daily requirement distributions were adjusted to assume low bioavailability for iron and zinc based on a diet of unrefined grains.

Subnational assessment is presented by geography and socio-economic position for the overall MPI (Supplementary Fig. 1) and individual micronutrients (Fig. 1). Geographically, the countries that had the greatest variation in overall MPIs between lowest and highest (Δ) administrative level 1 (ADM1) regions were Nigeria (Δ0.53) and Ghana (Δ0.35), where, in both contexts, the overall MPI was greater in the north of the country than in the south. The micronutrients contributing most to geographic overall MPI variation differed between countries as presented in Supplementary Table 1. For vitamin B_12_, higher vulnerability to inadequacy was concentrated in Niger, Benin, northern regions of Burkina Faso (Sahel, Est, Boucle du Mouhoun), southern regions of Mali (Koulikoro, Ségou, Kayes), and northern and eastern regions of Nigeria (Sokoto, Jigawa, Kano, Taraba, Bauchi). While vulnerability to vitamin A inadequacy was high across the region (national range: 46–94%), vulnerability was highest in Mali, Burkina Faso, Niger, Nigeria, northern regions of Ghana (upper west, northern, upper east) and the northern regions of Togo (Savanes) and Benin (Atacora, Alibori, Borgou). Vulnerability to iron inadequacy was elevated across much of the region, with several subnational pockets showing particularly high vulnerability, including Ziguinchor in Senegal (89%), Oio in Guinea-Bissau (86%), Bamako in Mali (85%) and Grand Lomé in Togo (83%).

Disaggregating the overall MPI by wealth quintiles showed that poorer populations are more vulnerable to inadequacy. First, for seven of the included countries, vulnerability to inadequacy incrementally increased with poorer populations according to the overall MPI, with higher values observed in poorer subgroups as observed in Nigeria (Δ0.41), Ghana (Δ0.35), Burkina Faso (Δ0.24), Senegal (Δ0.21), Mali (Δ0.16), Côte d’Ivoire (Δ0.15) and Benin (Δ0.13). Second, for Guinea-Bissau, Niger and Togo, vulnerability to inadequacy was high and within a closer range (Δ < 0.10) across all socio-economic positions. When each micronutrient was examined independently by socio-economic position within each country (Supplementary Table 2), all micronutrients showed increasing vulnerability with poorer populations, consistent with the overall MPI pattern across countries.

### Reach and consumption of vehicles in current regional standards

For wheat flour and edible oil, percentage reach (the percentage of the population consuming any amount of the staple) and median per capita daily consumption among consumers in grams per day (g d^−1^) per adult female equivalent (AFE) are presented nationally in Table 2 and sub-nationally in Supplementary Tables 3 and 4. These indicators reflect the current programme reach of the fortifiable (market-acquired and assumed to be industrially processed) form.

**Table 2:** National estimates of reach and per capita daily consumption of wheat flour, edible oil, rice, maize, millet and sorghum for ten countries in the West African region

Country	Wheat flour		Edible oil		Rice		Maize		Millet		Sorghum	
	Reach (%)	Per capita daily consumption (g d^−1^per AFE)^2^	Reach (%)	Per capita daily consumption (g d^−1^per AFE)	Reach (%)	Per capita daily consumption (g d^−1^per AFE)	Reach (%)	Per capita daily consumption (g d^−1^per AFE)	Reach (%)	Per capita daily consumption (g d^−1^per AFE)	Reach (%)	Per capita daily consumption (g d^−1^per AFE)
Benin	65	9 (4, 17)	33	32 (20, 52)	87	85 (56, 129)	91	180 (113, 279)	5	59 (34, 139)	13	143 (81, 288)
Burkina Faso	43	8 (4, 19)	55	13 (7, 24)	62	95 (58, 149)	64	144 (70, 258)	14	175 (80, 391)	24	231 (142, 365)
Côte d’Ivoire	69	10 (5, 18)	85	10 (5, 17)	84	171 (113,245)	42	46 (26, 81)	2	38 (27, 61)	0	36 (13, 75)
Ghana	15	7 (2, 19)	64	16 (7, 32)	75	89 (43, 178)	59	114 (57, 219)	4	42 (15, 102)	2	83 (40, 188)
Guinea-Bissau	69	8 (3, 22)	65	25 (12, 51)	99	277 (198, 371)	4	60 (40, 99)	4	77 (40, 135)	3	99 (55, 163)
Mali	58	11 (4, 23)	72	18 (10, 31)	92	148 (74, 243)	26	130 (70, 232)	49	145 (75, 253)	17	126 (62, 234)
Niger	28	8 (4, 17)	82	16 (9, 28)	81	140 (81, 227)	41	206 (117, 324)	83	320 (196, 479)	27	258 (158, 397)
Nigeria	73	19 (4, 38)	66	12 (8, 19)	87	72 (46, 108)	44	72 (39, 127)	25	90 (50, 162)	35	118 (61, 219)
Senegal	78	34 (7, 60)	80	47 (29, 69)	95	213 (155, 283)	29	58 (25, 117)	47	77 (35, 167)	3	84 (42, 176)
Togo	18	9 (5, 16)	60	15 (8, 26)	70	71 (40, 118)	94	183 (93, 299)	1	71 (41, 181)	2	150 (67, 269)

Reach is defined as the percentage of households that consume any amount of the staple. Per capita daily consumption in grams per day per AFE is defined as the median (IQR) total quantity of the staple apparently consumed among those consuming. For wheat flour and edible oil, reach and per capita daily consumption reflect the current programme reach of the fortifiable (market-acquired industrially processed) form. For rice, maize, millet and sorghum, they reflect total current consumption regardless of form, serving as a demand signal for prospective fortification.

**Fig. 2:**
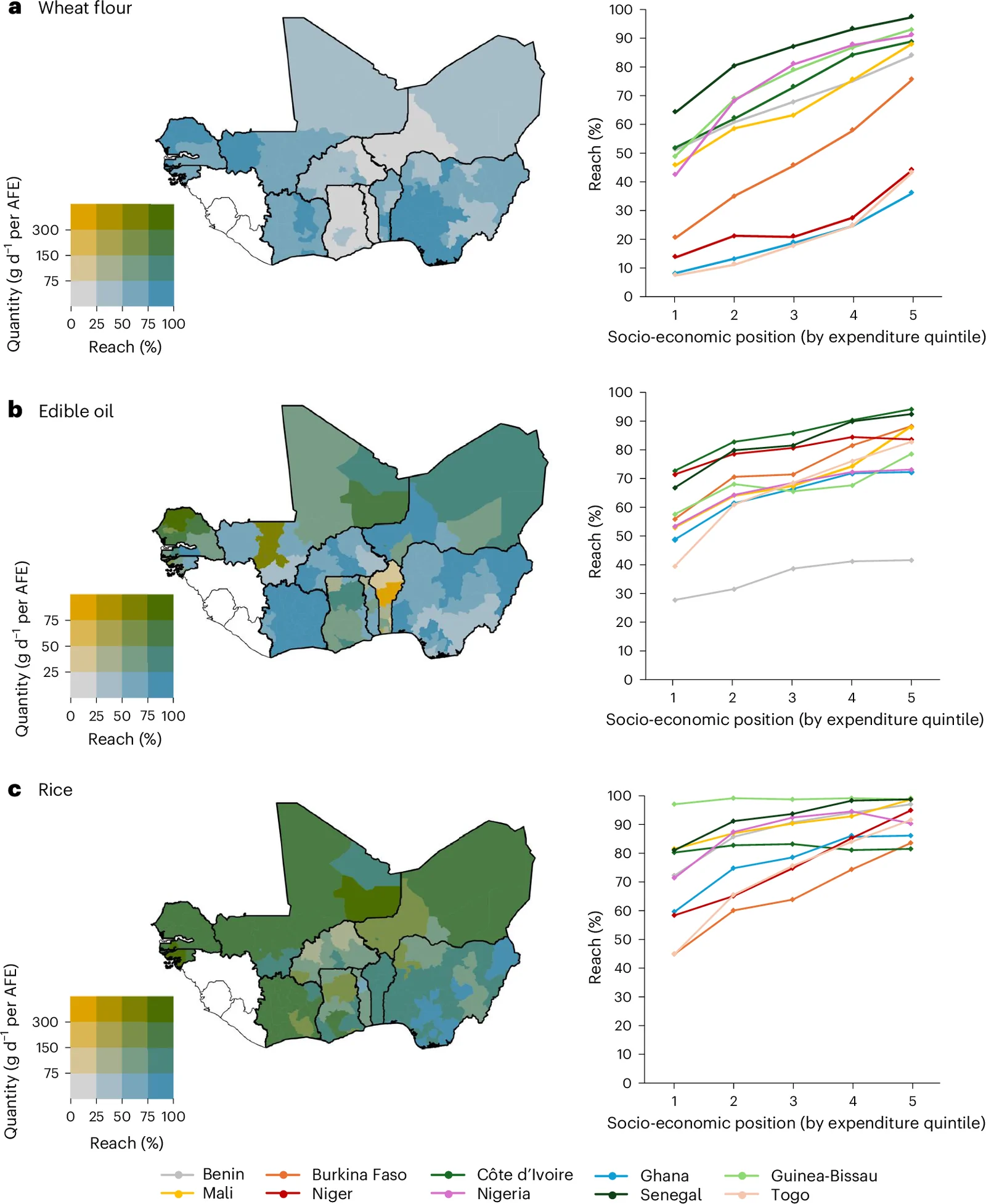
Geographic and socio-economic variation in access to fortifiable wheat flour, edible oil and rice for ten countries in the West African region. **a**, Wheat flour. **b**, Edible oil. **c**, Rice. Bivariate maps show the reach and per capita daily consumption quantity by first administrative units alongside reach by socio-economic position (poorest = 1 to wealthiest = 5). Reach is defined as the percentage of households that consume any amount of the staple. Quantity is defined as grams per day per AFE of the staple consumed, among consumers.

For vehicles included in existing regional standards, wheat flour reach varied widely between countries from 15% in Ghana to 78% in Senegal. Across all countries, national per capita daily wheat flour consumption ranged from 7 g d^−1^per AFE (interquartile range (IQR): 2, 19) to 34 g d^−1^per AFE (IQR: 7, 60), in which all countries were concentrated at the lower end of the lowest consumption category. When assessing subnational wheat flour consumption within all study countries (Fig. 2a), reach was lower among the poorest populations than among wealthier populations, with differences between the poorest and wealthiest quintiles ranging from 28 to 55 percentage points. Reach of edible oil also varied between countries (Fig. 2b), from 33% in Benin to 85% in Côte d’Ivoire, with the median per capita daily consumption ranging from 10 g d^−1^per AFE (IQR: 5, 17) to 47 g d^−1^per AFE (IQR: 29, 69). Similar to wheat flour, edible oil reach was incrementally lower among poorer populations across all countries, with the difference in reach between the wealthiest and poorest population quintiles ranging from 13 to 43 percentage points.

### Consumption patterns of candidate vehicles considered for inclusion

For rice, maize, millet and sorghum, or staple foods considered as candidate vehicles, consumption patterns are presented nationally in Table 2 and sub-nationally in Supplementary Tables 3 and 4. These indicators reflect total current consumption regardless of form, serving as a demand signal for prospective fortification.

**Fig. 3:**
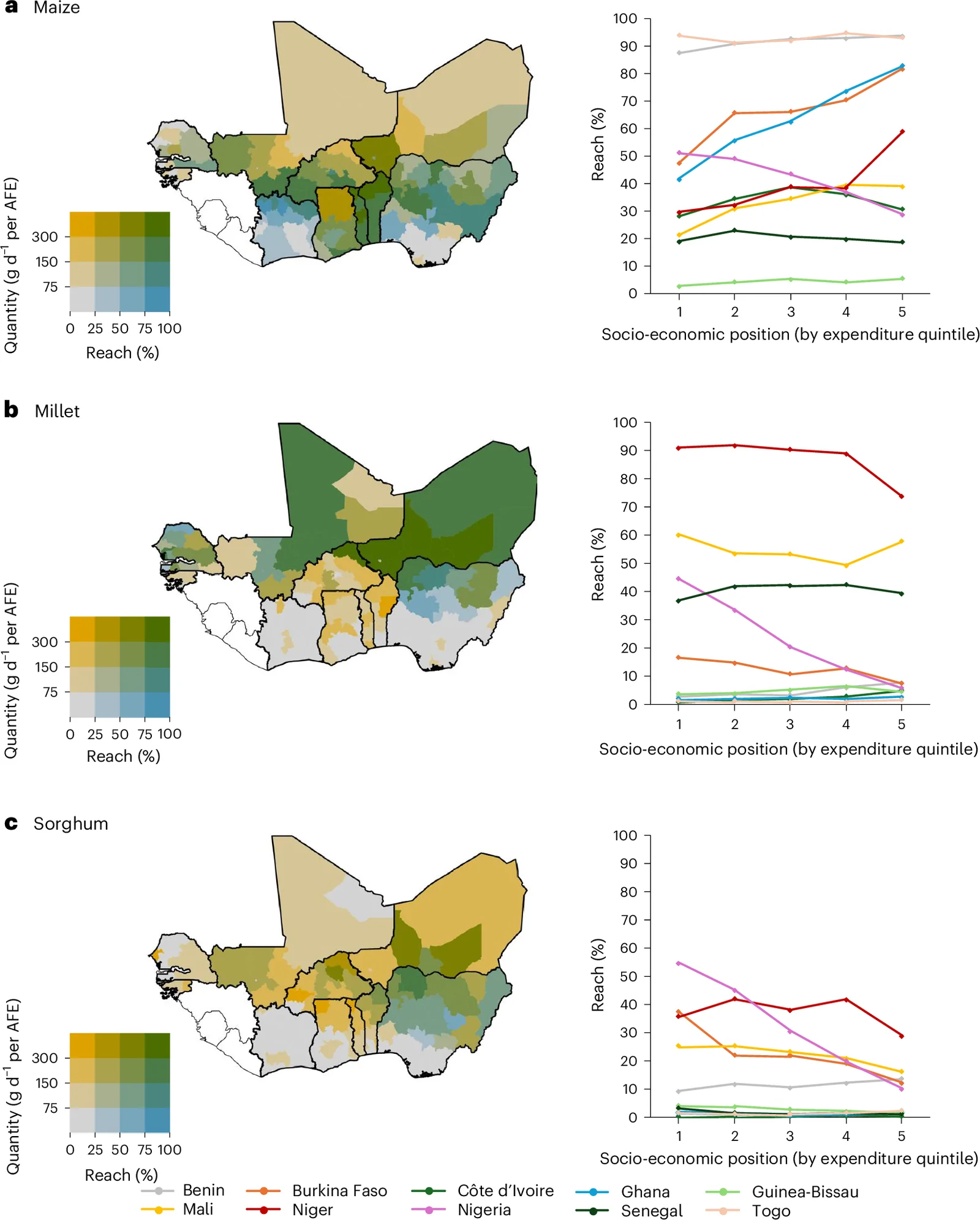
Geographic and socio-economic variation in access to fortifiable maize, millet and sorghum for ten countries in the West African region. **a**, Maize. **b**, Millet. **c**, Sorghum. Bivariate maps show the reach and per capita daily consumption quantity by first administrative units alongside reach by socio-economic position (poorest = 1 to wealthiest = 5). Reach is defined as the percentage of households that consume any amount of the staple. Quantity is defined as grams per day per AFE of the staple consumed among consumers.

Rice had the highest reach of all included staples, ranging from 62% to 95% of the population across countries. At national levels, per capita daily consumption varied by country: Côte d’Ivoire, Guinea-Bissau and Senegal fell within the 150–300 g d^−1^ range; Benin, Burkina Faso, Ghana, Mali and Niger within the 75–149 g d^−1^ range; and Nigeria and Togo below 75 g d^−1^ (Fig. 2c). Maize consumption was high in several countries, with the countries having the highest maize reach being Togo (94%), Benin (91%), Burkina Faso (64%), Ghana (59%) and Nigeria (44%) (Fig. 3a). By socio-economic position, reach was equally high across all socio-economic positions in Benin and Togo. Reach was incrementally lower in poorer populations in Burkina Faso and Ghana, and higher in poorer populations in Nigeria. For other candidate vehicles, populations consuming millet and sorghum were concentrated in certain countries, predominantly within the Sahel region (Fig. 3b,c). Millet reach was highest in Niger (83%), Mali (49%), Senegal (47%) and Nigeria (25%, concentrated in the north) with per capita daily consumption ranging from 71 g d^−1^per AFE (IQR: 41, 181) to 320 g d^−1^per AFE (IQR: 196, 479) in these countries. For these countries, millet reach was equal across all socio-economic positions, with the exception of Nigeria where poorer populations had incrementally higher millet reach (44%) than wealthy populations (6%). Sorghum reach was highest in Nigeria (35%, concentrated in the north), Niger (27%), Burkina Faso (24%) and Mali (17%), with median per capita daily consumption ranging in these countries from 118 g d^−1^per AFE (IQR: 61, 219) to 258 g d^−1^ per AFE (IQR: 158, 397). In all of these countries, sorghum reach was higher among the poorest populations than among the wealthiest, indicating that poorer expenditure quintiles were associated with higher sorghum reach.

### Public health potential of fortifiable staple foods

Table 3 presents the public health potential of each vehicle by country, expressed as the proportion of the population with potential to benefit (vehicle-specific MPI greater than 0.5) and vehicle coverage (proportion of the beneficiary population consuming the vehicle). In countries where vehicle coverage was greater than 10%, the consumption quantity (g d^−1^ per AFE) and corresponding consumption category among consumers are also presented.

**Table 3:** Public health potential of each vehicle for ten countries in the West African region based on vehicle consumption among potential beneficiaries

Country	Vehicle	Beneficiary population share (%)	Of the beneficiary population		
			Coverage (%)	Median (IQR) PCDC	Consumption category
Benin	Wheat flour	69	64	14 (7, 25)	Lowest
	Edible oil	47	29	33 (21, 53)	Mid-low
	Rice	62	86	76 (51, 110)	Mid-low
	Maize	69	90	162 (105, 239)	Mid-high
	Millet	72	4	–	–
	Sorghum	72	10	–	–
Burkina Faso	Wheat flour	65	38	10 (5, 20)	Lowest
	Edible oil	94	74	15 (8, 29)	Lowest
	Rice	58	57	78 (49, 122)	Mid-low
	Maize	65	62	153 (79, 261)	Mid-high
	Millet	66	12	165 (72, 323)	Mid-high
	Sorghum	66	27	225 (141, 358)	Mid-high
Côte d’Ivoire	Wheat flour	75	69	16 (10, 24)	Lowest
	Edible oil	74	86	9 (5, 16)	Lowest
	Rice	72	79	164 (110, 231)	Mid-high
	Maize	75	30	43 (25, 72)	Lowest
	Millet	76	2	–	–
	Sorghum	76	1	–	–
Ghana	Wheat flour	58	18	5 (1, 16)	Lowest
	Edible oil	60	54	13 (6, 28)	Lowest
	Rice	56	68	77 (40, 139)	Mid-low
	Maize	58	46	68 (40, 111)	Lowest
	Millet	57	2	–	–
	Sorghum	57	1	–	–
Guinea-Bissau	Wheat flour	81	73	18 (9, 32)	Lowest
	Edible oil	46	65	24 (11, 45)	Lowest
	Rice	79	99	277 (213, 362)	Mid-high
	Maize	81	4	–	–
	Millet	81	4	–	–
	Sorghum	81	3	–	–
Mali	Wheat flour	80	65	17 (8, 29)	Lowest
	Edible oil	85	68	18 (10, 29)	Lowest
	Rice	76	90	145 (75, 228)	Mid-low
	Maize	80	34	132 (72, 222)	Mid-low
	Millet	80	52	121 (67, 207)	Mid-low
	Sorghum	80	23	118 (60, 215)	Mid-low
Niger	Wheat flour	70	23	10 (4, 20)	Lowest
	Edible oil	94	80	16 (9, 27)	Lowest
	Rice	55	81	142 (82, 232)	Mid-low
	Maize	70	42	184 (110, 288)	Mid-high
	Millet	77	85	284 (173, 407)	Mid-high
	Sorghum	77	33	227 (143, 327)	Mid-high
Nigeria	Wheat flour	62	73	27 (17, 46)	Lowest
	Edible oil	84	68	12 (8, 18)	Lowest
	Rice	55	86	64 (42, 97)	Lowest
	Maize	62	40	73 (40, 124)	Lowest
	Millet	63	23	86 (48, 149)	Mid-low
	Sorghum	63	31	115 (61, 212)	Mid-low
Senegal	Wheat flour	76	83	46 (27, 64)	Lowest
	Edible oil	63	78	44 (28, 65)	Mid-low
	Rice	73	90	211 (161, 271)	Mid-high
	Maize	82	1	–	–
	Millet	77	37	59 (30, 130)	Lowest
	Sorghum	77	1	–	–
Togo	Wheat flour	81	19	10 (5, 17)	Lowest
	Edible oil	58	62	14 (8, 25)	Lowest
	Rice	75	71	63 (37, 102)	Lowest
	Maize	81	92	166 (83, 274)	Mid-high
	Millet	82	1	–	–
	Sorghum	82	1	–	–

Estimates from the EHCVM (2021–2022), GLSS (2016–2017) and NLSS (2018–2019). Beneficiary population: households with vehicle-specific MPI > 0.5 (for edible oil, which delivers only vitamin A, defined via the cut-point approach against the harmonized average requirement). For wheat flour and edible oil, coverage and consumption reflect the current programme reach of the fortifiable form; for rice, maize, millet and sorghum, they reflect total consumption regardless of form, indicating demand and opportunity for future fortification. Coverage: percentage of the beneficiary population consuming the vehicle. Per capita daily consumption (PCDC) (median, IQR; grams per AFE per day) is shown where coverage >10%. Consumer categories: low, mid-low, mid-high and high use <75 g, 75–149 g, 150–300 g and > 300 g, respectively, for staple grains, and <25 g, 25–49 g, 50–75 g and >75 g, respectively, for edible oil. See Methods for vehicle-specific MPI construction and bioavailability assumptions.

Across all countries and vehicles, fortification has the potential to benefit large proportions of the population, ranging from 46% to 94%. The highest proportions of populations that could benefit from fortification of staple grains, which can deliver iron, folate, vitamin B_12_ and zinc collectively, were observed in Togo (75–82%), Guinea-Bissau (79–81%) and Mali (76–80%). For edible oil fortification with vitamin A, the largest potential beneficiary populations were observed in Burkina Faso (94%), Niger (94%), Mali (85%) and Nigeria (84%).

For wheat flour and edible oil, these indicators characterize how well existing fortification programmes can cover vulnerable populations. Wheat flour showed variable coverage among the beneficiary population, ranging from 18% to 81% across countries. However, per capita daily consumption was consistently low, with median consumption ranging from 5 g d^−1^per AFE (IQR: 1, 16) to 46 g d^−1^per AFE (IQR: 27, 64), corresponding to the low consumption category in all countries. Edible oil showed considerable heterogeneity, with coverage ranging from 29% to 86%. However, per capita daily consumption corresponded to the lowest consumption category in all countries, except Senegal and Benin, which corresponded to mid-low.

For rice, maize, millet and sorghum, these indicators characterize the alignment between current staple consumption and vulnerability, informing where future fortification programmes could target public health need. Relative to other candidate vehicles, rice consistently presented high coverage (range: 57% to 99%) and higher median per capita daily consumption (range: 63 g d^−1^per AFE to 277 g d^−1^ per AFE) across all countries, with particularly high coverage and per capita daily consumption observed in Guinea-Bissau, Senegal and Mali. Maize showed considerable heterogeneity across countries. Coverage ranged from 1% to 92%, with higher coverage generally corresponding to higher per capita daily consumption in countries where maize was more widely consumed. Coverage of millet and sorghum was above 10% in Burkina Faso, Mali, Niger, Nigeria and Senegal (only millet), where, in these countries, median per capita daily consumption suggested mid-low to mid-high (for example, 75–300 g d^−1^ per AFE) consumption levels.

Sensitivity analyses applying a higher vehicle-specific MPI threshold (Supplementary Table 5) and incorporating normative fortification specifications (Supplementary Table 6) produced consistent patterns. A further sensitivity analysis using more favourable bioavailability assumptions for iron (15% absorption) and zinc (refined diet) reduced estimated vulnerability by approximately 20 and 30 percentage points, respectively, although high vulnerability and the socio-economic gradient persisted across all countries (Supplementary Table 7).

## Discussion

This study examined variability in vulnerability to inadequate micronutrient intake and access to fortifiable staple foods across West Africa and their alignment to provide an evidence base to strengthen regional food fortification efforts equitably. Vulnerability to inadequate intake was high across the region, driven largely by consistently elevated vulnerabilities to vitamin A, folate, iron and zinc inadequacies. There were elevated vulnerabilities to vitamin B_12_ inadequacy in Niger and sub-nationally localized geographies in other countries in the region. Wheat flour and edible oil appear to have limits as fortification vehicles: national per capita consumption medians were low for wheat flour (5–46 g d^−1^ per AFE) and mid-low for edible oil (9–44 g d^−1^ per AFE) among the population in need, and both vehicles had the lowest reach among the poorest populations across all countries in the region. Pre-existing demand for other staple grains presents potential opportunities for fortification in West Africa, with rice showing high coverage and per capita daily consumption among the population in need in most countries and millet and sorghum showing consumption concentrated in economically vulnerable populations and geographies with elevated vulnerability to inadequate micronutrient intake in the Sahel and certain regions of other countries. The cross-country variation in vulnerability profiles and vehicle access observed here shows that no single national configuration fits the entire region.

This study provides a structured approach for assessing the public health potential of fortifiable staple foods by linking vulnerability to inadequate micronutrient intake with patterns of vehicle consumption. The framework, which is complementary to other frameworks used to evaluate fortification programmes^25,26^, combines three components: the proportion of the population with the potential to benefit from each vehicle based on vulnerability to the micronutrients it can deliver, the coverage of the vehicle among this population and the quantity consumed among those reached. Vehicles are most appropriate for fortification where high levels of vulnerability coincide with high coverage and sufficient consumption, as all three conditions are required for fortification to meaningfully contribute to reducing risk of inadequate micronutrient intake. Where these conditions are not met, fortification of a given vehicle is unlikely to substantially reduce risk of inadequacy, indicating the need for complementary interventions to increase intake where fortification alone is insufficient. This approach provides a generalizable and reproducible framework that can be applied across settings where household consumption and expenditure survey (HCES) data exist and are updated over time as dietary patterns and nutritional vulnerabilities evolve. This study illustrates an example of how combining information on vulnerability and vehicle access can support more systematic and equitable prioritization of fortification strategies.

Across the West African region, vulnerability to inadequate micronutrient intake was widespread, with the poorest populations facing the greatest vulnerability. Elevated vulnerability to inadequate intake across all countries aligns with findings using biomarkers from recent national micronutrient surveys in Ghana^27^, Senegal^28^ and Nigeria^29^. As summarized in Supplementary Table 8, these surveys report high prevalence of deficiencies in vitamin A (12–31%) and zinc (35%) for children under five, and folate deficiencies (50–91%) and anaemia (22–29%) for women of reproductive age, all of which constitute moderate to severe public health problems according to global guidance^30–32^. These findings support our modelled estimates suggesting elevated vulnerability to inadequate intake across West Africa. These findings are consistent with existing estimates from sub-Saharan Africa, which indicate that approximately 62% of preschool-aged children have at least one deficiency in vitamin A, zinc or iron, and that nearly 80% of non-pregnant, non-lactating women of reproductive age are affected by at least one of these deficiencies^2^. The findings from our modelled estimates and national micronutrient survey data suggest that there is a high burden of micronutrient deficiencies that are probably driven by inadequate micronutrient intake, justifying the need for population-level diet interventions, such as food fortification, across the West African region.

The wheat flour and edible oil fortification pathways operate through established large-scale industrial supply chains, with vehicle selection informed by consumption patterns, industrial processing characteristics and other criteria outlined in global guidance^26^. Wheat flour is frequently the entry point for staple grain fortification in African countries, given its centralized production, established processing capacity, and existing quality control and regulatory frameworks. Despite its feasibility, this study found that wheat flour may be limited as a vehicle with inequitable access reflected in both low reach and low per capita consumption among the poorest populations within countries. Evidence from other data sources supports these findings, indicating that the limited reach of wheat flour is consistent across methods and settings. National survey data from Nigeria^29^ show that only 19% of the poorest households consume wheat flour products compared with 35% overall, and similar inequalities have been reported in Ethiopia^33^, Malawi^34^ and Tanzania^35^. These findings were also broadly consistent with national per capita availability estimates derived from national food supply accounts (Supplementary Table 9), which similarly indicate relatively low wheat availability across most countries in the region. Senegal represents a partial exception, with higher reach (78%) and per capita daily consumption (34 g d^−1^ per AFE) compared with other countries in the region.

Edible oil shows similar inequities, with low consumption among poorer households across the region, which limits its ability to serve as an equitable vehicle. This is especially relevant for vitamin A, as edible oil is the most appropriate and widely used vehicle for its fortification (similarly for vitamin D), given the limited alternatives among staple grains. This is an important gap because vitamin A inadequacy was consistently high, and staple grain vehicles have well-recognized technical challenges for vitamin A fortification linked to fat solubility and shelf life^36^. This implies that together wheat flour and edible oil fortification are best positioned as programmatically practical entry points that can reach substantial segments of the population, particularly in urban and wealthier subpopulations. Additional programmes are needed to extend micronutrient programme equity.

For the candidate vehicles considered for inclusion in regional standards (that is, rice, maize, millet and sorghum), comparable industrial delivery systems for fortification are often not yet established at scale^37^. Unlike wheat flour and edible oil, which have existing supply chains that determine fortification feasibility, these vehicles deal with the the primary question of whether consumption is sufficient to justify building that infrastructure. Total consumption captures the level of demand that could justify investment in supply chains, while the current fortifiable share reflects only the existing open-market structure through which fortification can presently be delivered^38^. Achieving the economies of scale needed for fortification in these settings may require producer aggregation, institutional procurement and integration with social protection systems. Examples of this include experiences documented in maize and rice fortification programmes in East Africa and other low- and middle-income settings^39,40^.

Rice presented substantial public health potential if fortified, as characterized in this study, with more advanced stages of programme development already underway in West Africa. In this study, rice showed consistently high coverage (57–99%) and per capita daily consumption (63–277 g d^−1^ per AFE) among the populations with potential to benefit across most countries and socio-economic positions within countries. Rice fortification in West Africa operates through two pathways: fortification of imported rice at the border, with existing examples from Senegal^10^, and fortification of domestically produced rice through the consolidation of small- and medium-scale milling. Programme development for domestically produced rice fortification is already underway in West Africa, drawing on operational pilots that have tested implementation models in the region^20,41^, fortification technologies^42^ and international technical guidance^13,43^ to inform the design of regional guidance. While these pilots have advanced programme design, they have also revealed operational challenges that remain from a highly decentralized network of small- and medium-scale mills^44^. Decentralized milling limits consistency in blending, quality assurance and regulatory monitoring, and access to fortified rice kernels remains uneven across the region^45^. As a result, populations that consume the most rice may have limited access to a fortified product, unless capacity for supply chains and milling infrastructure can be strengthened. Closing these gaps will require further research, including mapping intraregional rice trade flows and milling locations, and clarifying the contributions of industrially milled, locally milled and imported rice to consumer demand for rice.

Maize, millet and sorghum present opportunities because their consumption patterns align closely with the populations facing the greatest vulnerability to inadequate micronutrient intake, although fortification pathways for these staple grains remain at an earlier developmental stage. This study identified clear geographic and socio-economic patterns in grain consumption, with millet and sorghum most commonly consumed in the Sahel and among poorer households, and maize showing higher consumption in Benin, Togo and certain regions within Burkina Faso, Ghana and Nigeria. Fortification of sorghum and millet at scale would depend on industrial milling and food-technology capacity that remain at the research and development stage in the region. Previous experimental research shows that sorghum and millet can be fortified with iron, zinc and folic acid^22,46^, showing promising results for nutrient retention, product stability and sensory acceptability under experimental conditions. For maize, the predominance of small-scale, decentralized milling systems across parts of West Africa presents challenges for ensuring consistent fortification. A recent study combining fortification assessment surveys across Africa found that while maize reach averaged 73%, only 37% was fortifiable, presenting a challenging feasibility gap in expanding programme coverage^47^. Closing these gaps will require further research, including validating food technology for fortifying these grains at scale and developing industrial milling capacity in the region.

These findings reinforce the framework laid out in the World Health Organization (WHO) and Food and Agriculture Organization of the United Nations (FAO) Guidelines on Food Fortification with Micronutrients, in which harmonized international or regional reference parameters support trade and regulation while national decisions determine which vehicles to mandate and calibrate fortification levels to local contexts^26^. In West Africa, harmonization efforts through regional governing institutions have produced meaningful progress^7^. Our findings suggest that regional fortification guidance should harmonize the elements that support intraregional trade, regulatory infrastructure and cross-country comparability such as the candidate vehicle menu, the micronutrient parameters and a shared monitoring framework. The national policy determines which subset of those vehicles to mandate and calibrates fortification levels to country-specific dietary patterns, production systems and vulnerability profiles. The diversity of consumption patterns across West Africa documented in this study underscores the value of an expanded regional menu of candidate vehicles and parameters, so that countries can select the vehicles best suited to their context.

This study had several limitations. First, this analysis reflects current consumption patterns, which may not fully capture how access and affordability of fortified staples could shift once fortification is implemented and consumer preferences and market prices adjust. Second, the market-purchased share used as a proxy for the fortifiable form of vehicles in current regional standards is imperfect, in which informal channels carry some industrially processed product, and some market-purchased grain is milled locally. For candidate vehicles, consumption is treated as a demand signal and does not characterize the processing capacity that would need to be built for fortification at scale. Third, the underlying data also permit quantitative modelling of fortification’s impact on intake gaps, the prevalence of inadequacy and the risk of high intakes, but this analysis was scoped to the upstream task of characterizing which vehicles align with vulnerability across the region. Setting fortification standards, whether establishing new ones or adapting existing ones, should also be informed by direct impact modelling of single and combined vehicles, a logical next stage building on the priorities identified here. Fourth, salt and bouillon could not be included in the analysis despite interest in their fortification in the region, as bouillon cubes are sometimes absent from HCES food item lists, and discretionary salt intake quantified using HCES dietary recall methods faces issues of low precision^48^. Fifth, HCES data for Nigeria and Ghana were drawn from different survey instruments and time periods than those used for other countries, which may affect cross-country comparability. Lastly, the analysis was limited to West African countries with available HCES data, and although the sample population captures the majority of the region’s population, five countries were omitted (that is, Cape Verde, the Gambia, Guinea, Liberia and Sierra Leone) owing to data unavailability.

In conclusion, this study assessed the public health potential of fortifiable staple foods across West Africa by linking vulnerability to inadequate micronutrient intake with patterns of current and potential vehicle consumption. Wheat flour and edible oil represent established programmatic entry points, but their limited reach among the poorest populations constrains their equity contributions. Rice shows consistent coverage and consumption across most of the region, with programme development already underway, although closing the gap between demand and fortifiable supply will require further development. Maize, millet and sorghum present opportunities aligned with the most nutritionally vulnerable populations, but remain at earlier stages of fortification programme development. By integrating vulnerability, vehicle coverage and the conditions needed for fortification to meaningfully reduce inadequacy, the framework provides a reproducible basis for identifying where investment is most likely to yield equitable impact. Regional multilateral mechanisms can build on these findings to harmonize the candidate vehicle menu, micronutrient parameters and monitoring frameworks that enable trade and regulatory comparability, while national policy determines which vehicles to mandate based on country-specific dietary patterns and production systems. Building practical channels for regular exchange of experience and insights among countries across the West African region will be critical to maintaining momentum and coherence across regional fortification efforts.

## Methods

### Model framework and parameters from dietary data

This study used the nutrient supply model framework, which has been refined through repeated application across diverse country contexts^34,49^. The framework draws on nationally representative HCES, a type of economic survey that collects microdata on household-level food consumption using standardized methods^50^. This includes a fixed food item list, multi-day recall periods (typically 7 days), identification of food acquisition sources and prices, and emphasis on foods consumed collectively by the household. Alongside food consumption, these surveys also capture a wide range of socio-economic and living standard indicators, including household demographics, expenditures, income sources, assets, educational attainment and health conditions. The framework generates comparable estimates of household nutrient supply^51^ that can be compared with recommended intakes and used as a proxy to identify nutritionally vulnerable populations and highlight opportunities for micronutrient interventions, including food fortification.

This study included ten West African countries, selected to include as many members of the ECOWAS and/or the Alliance des États du Sahel as possible where recent nationally representative HCES were available (within the past 10 years). Most countries participated in the Union Économique et Monétaire Ouest-Africaine’s standardized Enquête Harmonisée sur les Conditions de Vie des Ménages (EHCVM) 2021–2022, a regionally coordinated HCES designed to ensure comparability of socio-economic and consumption indicators across countries. The included EHCVM surveys were conducted in Benin, Burkina Faso, Côte d’Ivoire, Guinea-Bissau, Mali, Niger, Senegal and Togo. For Ghana and Nigeria, data were obtained from the Ghana Living Standards Survey (GLSS; 2016–2017) and the Nigeria Living Standards Survey (NLSS; 2018–2019) and were prepared ex post to align with the EHCVM structure for regional comparability. The survey type, sample size, number of food items and time horizon for each country are summarized in Supplementary Table 10.

### Data preparation

Food consumption data from each country’s HCES were processed using standardized procedures described in detail elsewhere^34^ to ensure high quality and consistency across countries. Indicators for the analysis were then derived from the food consumption module, with all foods included for the assessment of nutritional vulnerability (objective 1) and each staple (that is, grains, grain-based products, edible oils) isolated for the analysis of fortifiable food vehicles (objective 2). For the EHCVM surveys and NLSS, reported quantities of food items were converted into metric units using provided conversion factors and adjusted to exclude inedible parts of foods (for example, the skin of a banana), to generate estimates of daily apparent household consumption. For Ghana, household food expenditure estimates were collected rather than food consumption quantities. Consumption quantity estimates, previously derived from these expenditure data by the Micronutrient Intervention Modeling (MINIMOD) project^52^, were used as the inputs for this analysis and were subsequently adjusted to exclude inedible portions of foods as described above. To address extreme values probably reflecting recall errors, outliers were identified separately for each country and food item among households consuming that item: reported quantities were log transformed, and any value exceeding the consumer mean plus four standard deviations on the log scale was flagged. Flagged values were replaced with the geometric mean of consumer-reported quantities for that food item in that country.

### Vulnerability to inadequate micronutrient intake indicators

The primary indicator used to assess a population’s vulnerability to inadequate micronutrient intake was the overall MPI of five priority micronutrients (that is, vitamin A, zinc, iron, folate, vitamin B_12_)^53^, and the secondary indicator was the percentage of the population vulnerable to inadequacy for each micronutrient^51^.

These five micronutrients were selected because their deficiencies contribute to maternal and child mortality through well-established biological pathways and are therefore commonly prioritized in LSFF programmes. Vitamin A and zinc deficiencies increase child mortality risk by impairing immune function and thereby increasing the incidence and severity of infections, particularly diarrhoea (both) and measles (vitamin A)^3,54–56^. Iron, folate and vitamin B_12_ deficiencies elevate maternal mortality risk via severe anaemia and contribute to adverse birth outcomes^57^. Iron deficiency is the leading cause of anaemia globally^58^, while folate and vitamin B_12_ deficiencies clinically manifest as megaloblastic anaemia through impaired DNA synthesis^59,60^. Folate deficiency additionally contributes to neonatal and infant mortality through increased risk of neural tube defects^61^. These micronutrient deficiencies have also been linked to longer-term developmental outcomes, including impaired linear growth and cognitive development^3^, aligning with priorities reflected in the United Nations Sustainable Development Goals.

For each micronutrient, every food item reportedly consumed in the HCES was matched to corresponding nutrient composition values from relevant food composition tables (FCTs). Food item matches previously completed by the MINIMOD project for the 2018–2019 EHCVM data in Senegal and Burkina Faso^62,63^ were used as a foundation for the 2021–2022 EHCVM food item matches undertaken in this study. For EHCVM countries, the Senegal food item matches were applied to coastal countries (that is, Benin, Côte d’Ivoire, Guinea-Bissau, Senegal, Togo), while Burkina Faso food item matches were applied to Sahel countries (that is, Burkina Faso, Mali, Niger) with country-specific adjustment applied to all contexts (for example, country-specific fish species and green leafy vegetable varieties). For Nigeria and Ghana, food item matches were drawn from other previous studies conducted by the MINIMOD project specific to those countries^12,64^. In all countries, the 2019 West African Food Composition Table (WAFCT) served as the primary source of nutrient values, and the Malawi FCT, the USDA FoodData Central and the Nutrition Coordinating Center Nutrient Database for Standard Reference were used when no suitable WAFCT match was available^65–67^. For aggregate items, such as ‘melon/watermelon’, nutrient composition values were assigned as averages of multiple FCT entries.

Household micronutrient supply was derived by aggregating micronutrient values from all reported foods and dividing the total by household size standardized to AFEs. The primary assumption in the AFE approach is that household micronutrient supply is distributed across household members proportionally according to each member’s mean daily energy requirements^68^ estimated using information on age and sex of household members from the HCES household roster. Mean daily energy requirements were drawn from the FAO, WHO and United Nations University (UNU) Human Energy Requirements report and applied uniformly across all ten countries^69^. AFEs are defined as the ratio of each household member’s mean daily energy requirement to that of a reference non-pregnant non-lactating 18–29-year-old woman^69^. In households with a child under 2 years of age, two adjustments were made: the woman of reproductive age was allocated additional energy requirements for breastfeeding^70^, and the child’s energy requirement was reduced to exclude the contribution of breastmilk^34,71^. All other women of reproductive age (including those who may have been pregnant, which was not included in the HCESs) were assigned the energy requirement of a non-pregnant non-breastfeeding woman. The AFE approach addresses variation in energy requirements across household members but does not correct for household-level reporting error. This distinction is a recognized limitation of the HCES-based nutrient supply framework^51^, and MPI estimates should be interpreted as population-level proxies for vulnerability to inadequate intake rather than precise measures of individual consumption.

For the overall MPI, apparent intake distributions for each micronutrient were transformed into probabilities of inadequacy using a daily nutrient requirement distribution. For vitamin A, zinc, folate and vitamin B_12_, probabilities of inadequacy used the harmonized average requirement (H-AR) of a non-pregnant, non-lactating 18–29-year-old woman^72^. Probability of iron inadequacy was estimated using the right-skewed requirements distribution for 18–29-year-old women^73^. The daily requirement distributions were adjusted to assume low bioavailability for iron and zinc based on a diet of unrefined grains in line with dietary patterns observed in the data. As a sensitivity analysis, vulnerability to inadequate iron and zinc intake was re-estimated using more favourable bioavailability assumptions: 15% absorption for iron^26^ and requirements corresponding to a refined diet for zinc^72^. The overall MPI was calculated as the arithmetic mean of the probabilities of inadequacy across the five micronutrients.

Individually for vitamin A, zinc, folate and vitamin B_12_, the percentage of the population vulnerable to inadequacy was estimated using the cut-point approach, in which apparent intakes lower than the H-AR for each corresponding micronutrient were identified as vulnerable to inadequate intake^72,74^. The population vulnerable to iron inadequacy was estimated using the full probability approach to account for the skewed iron requirement distribution for 18–29-year-old women^73^.

### Reach and consumption of vehicles in current regional standards

Wheat flour and edible oil are both included in current ECOWAS standards^7^ and supported by well-defined food technologies, existing quality assurance and monitoring systems, and supply chains operating at industrial scale. These vehicles were assessed using the indicators reach and per capita daily consumption^34,75^, which together contextualize consumer demand for fortification. Reach is defined as the percentage of households that consume any amount of the vehicle. Per capita daily consumption (presented in grams per day per AFE) is defined as the median quantity of the vehicle consumed among consumers. Consumption was measured as the fortifiable share, defined as the portion produced through industrial processing. Consumption data were restricted to food items purchased from the market, used here as a proxy for industrially processed product.

### Consumption patterns of candidate vehicles considered for inclusion

For rice, maize, millet and sorghum, fortification pathways do not exist or are underdeveloped. Consumption was measured as the total consumed quantity to reflect the full demand that would justify and shape future fortification infrastructure. Reach and per capita daily consumption were estimated for these vehicles using the same operational definitions. Consumption data were extracted irrespective of source (that is, home produced, market acquired, or gifted or other), as the analytical objective for these vehicles is to characterize total demand rather than reach of currently fortifiable forms. A full list of the HCES food items assigned to each vehicle for each country is provided in Supplementary Table 11.

### Public health potential of fortifiable staple foods

The public health potential of each vehicle, or whether or not each vehicle has the potential to provide additional micronutrients in sufficient amounts to the population in need, was assessed using a framework derived from the ‘household monitoring’ recommendations in the WHO Guidelines on Food Fortification with Micronutrients^26^. This framework combines three components: the proportion of the population with the potential to benefit from each vehicle based on vulnerability to the micronutrients it can deliver, the coverage of the vehicle among this population and the quantity of the vehicle consumed among those reached, as an indicator of its potential contribution to micronutrient intake.

The population with the potential to benefit was defined for each vehicle at the national level based on the set of micronutrients that each vehicle could plausibly deliver (Supplementary Table 12). The micronutrients that each vehicle could plausibly deliver were informed by global normative and operational guidance for wheat flour^76^, edible oil^77^, rice^13,43^ and maize flour^14^, and by experimental studies describing feasible micronutrient fortification of millet and sorghum^22,46^. For each vehicle, a vehicle-specific MPI was constructed using only the micronutrients that could plausibly be delivered. Within this framework, households were classified as having the potential to benefit from the vehicle when the vehicle-specific MPI exceeded 0.5, that is, households that were more likely than not to be inadequate of the micronutrients the vehicle could deliver. For edible oil, which contains only vitamin A, households were classified by whether the apparent vitamin A intake fell below the harmonized-average requirement for a non-pregnant non-lactating 18–29-year-old woman^72^.

The public health potential, or the extent to which each vehicle could reduce risk of inadequacy among the aforementioned beneficiary populations, was assessed by examining characteristics of consumption of each vehicle among the vehicle-specific beneficiary population. Coverage, or the percentage of the beneficiary population that consumes the vehicle, was calculated for each vehicle. Coverage defines the maximum proportion of the beneficiary population that could potentially benefit from the vehicle’s fortification. In countries where vehicle coverage exceeded 10%, the median (IQR) per capita daily consumption among consumers, or the quantity of the vehicle consumed (in grams per day per AFE), was estimated. Median consumption quantities were used to classify countries into categories to characterize consumption patterns for each vehicle, with thresholds specified in the following section.

One-way sensitivity analyses were conducted to assess the robustness of parameter assumptions. First, the vehicle-specific MPI threshold was increased from 0.5 to 0.75 to examine a scenario adopting a more narrow definition of the beneficiary population that prioritizes households with higher vehicle-specific MPIs. Second, vitamin A was added to the vehicle-specific MPI for wheat flour, rice and maize to represent a scenario assuming full adherence to WHO fortification guidance, setting aside the retention and normative-guidance considerations that lead many West African countries to carry vitamin A primarily through edible oil rather than cereal vehicles^76,78^.

### Statistical analysis

The primary unit of analysis was the household. Estimates were first aggregated to the national level for each country and then stratified separately by geographic area (first administrative units; ADM1) and socio-economic position (defined by national household annual expenditure quintiles). Survey weights provided with the survey data were applied to obtain weighted summary statistics in all instances, to ensure representativeness at both national and subnational levels. Geographic boundaries for ADM1 were defined using shapefiles obtained from Open Street Map collated by the United Nations World Food Programme. Subpopulation assessment characterized within-country variability, defined as the range (Δ) between highest and lowest estimates within the subpopulation, and across-country variability, defined as consistent geographic (for example, east–west or north–south) or socio-economic gradients (for example, increasing or decreasing vulnerability with socio-economic positions).

Summary statistics were generated separately for each indicator according to its measurement scale. For nutritional vulnerability, the overall MPI was presented as a continuous mean probability ranging from 0 to 1, and individual micronutrients were expressed as the percentage of a population vulnerable to inadequacy. For staple consumption nationally, reach was presented as the percentage of households consuming any amount of the staple, and per capita daily consumption as the median with interquartile ranges among consumers to account for non-normal distributions. For staple grains sub-nationally, per capita daily consumption medians were assessed against the categorical thresholds defined for distinguishing fortification levels in the WHO guidelines for wheat flour fortification^76^ and World Food Programme specifications for rice fortification^42,43,79^, with four consumption ranges used to classify intake: low (<75 g d^−1^ per AFE), mid-low (75–149 g d^−1^ per AFE), mid-high (150–300 g d^−1^ per AFE) and high (>300 g d^−1^ per AFE). WHO guidance for edible oil fortification does not specify consumption-based thresholds^77^, so categories were derived from the quartiles of edible oil apparent consumption sub-nationally across all included countries: low (<25 g d^−1^ per AFE), mid-low (25–49 g d^−1^ per AFE), mid-high (50–75 g d^−1^ per AFE) and high (>75 g d^−1^ per AFE).

All analyses were conducted in R version 4.5.1.

### Reporting summary

Further information on research design is available in the Nature Portfolio Reporting Summary linked to this article.

## Supplementary information

Supplementary Information Supplementary Fig. 1 and Tables 1–12.

Reporting Summary

Peer Review File

## Data Availability

The household survey data underlying the vulnerability to inadequate intake estimates are held by third parties and are available from their original sources. The EHCVM and Nigeria Living Standards Survey data are available through the World Bank Microdata Library (https://microdata.worldbank.org/index.php/catalog/lsms), subject to user registration and acceptance of the Microdata Library’s terms of use, which do not permit redistribution of the data by third parties. The Ghana Living Standards Survey data are available from the Ghana Statistical Service under equivalent conditions (https://microdata.statsghana.gov.gh/index.php/catalog/97). The authors received no special access privileges to these data. The food item matches and nutrient composition values generated for this analysis, together with documentation of all data sources and how each was accessed, are available via Zenodo at https://doi.org/10.5281/zenodo.21470669 (ref. ^80^).

## Code availability

The R scripts for the HCES nutrient supply model used to clean, process and analyse the household survey data are available at https://github.com/MIMI-wfp/West-Africa-LSFF-Analysis (ref. ^80^).
