# Supplementary material for: Diversified food fortification portfolios can enhance coverage of micronutrient-vulnerable populations in West Africa
Source: Nat Food. 2026 Sep 2;7(9):902–16. doi: 10.1038/s43016-026-01412-2 (PMC13593565; doi:10.1038/s43016-026-01412-2)
Supplement: Supplementary file 1 [file 43016_2026_1412_MOESM1_ESM.pdf]

nature food

Supplementary information

https://doi.org/10.1038/s43016-026-01412-2

Diversified food fortification portfolios can enhance coverage of micronutrient-vulnerable populations in West Africa

In the format provided by the authors and unedited

Potential of fortifiable staple foods for addressing micronutrient vulnerability in West Africa

- SUPPLEMENTARY MATERIALS –

Table of Contents

Figure S1. *Subnational assessment of vulnerability to inadequate micronutrient intake by household mean probabilities of inadequacy (MPI) for vitamin A, folate, vitamin B12, iron, and zinc in ten West African countries, shown by (A) geography by first administrative units (ADM1) and (B) quintile of socioeconomic position (poorest = 1 to wealthiest = 5).* 3

Table S1. *Subnational-level vulnerability to inadequate micronutrient intake by administrative level 1 in Benin, Burkina Faso, and Cote D'Ivoire, including the mean probability of inadequacy (MPI) and the percentage of the population vulnerable to inadequate intake for vitamin A, folate, vitamin B12, iron, and zinc.* 4

Table S1 (continued). *Subnational-level vulnerability to inadequate micronutrient intake by administrative level 1 in Ghana, Guinea-Bissau, Mali, Niger, and Nigeria, including the mean probability of inadequacy (MPI) and the percentage of population vulnerable to inadequate intake for vitamin A, folate, vitamin B12, iron, and zinc.* 5

Table S1 (continued). *Subnational-level vulnerability to inadequate micronutrient intake by administrative level 1 in Nigeria (continued), Senegal, and Togo, including the mean probability of inadequacy (MPI) and the percentage of the population vulnerable to inadequate intake for vitamin A, folate, vitamin B12, iron, and zinc.* 6

Table S2. *Subnational-level vulnerability to inadequate micronutrient intake by socioeconomic wealth quintile in ten countries in the West African region, including the mean probability of inadequacy (MPI) and the percentage of the population vulnerable to inadequate intake for vitamin A, folate, vitamin B12, iron, and zinc.* 7

Table S3. *Subnational estimates of percent reach and per capita daily consumption (PCDC) in grams per day per adult female equivalent of wheat flour, edible oil, rice, maize, millet and sorghum at administrative level one in Benin, Burkina Faso, and Cote D'Ivoire.* 9

Table S3 (continued). *Subnational estimates of percent reach and per capita daily consumption (PCDC) in grams per day per adult female equivalent of wheat flour, edible oil, rice, maize, millet and sorghum at administrative level one in Ghana, Guinea-Bissau, Mali Niger, and Nigeria.* 10

Table S3 (continued). *Subnational estimates of percent reach and per capita daily consumption (PCDC) in grams per day per adult female equivalent of wheat flour, edible oil, rice, maize, millet and sorghum at administrative level one in Nigeria (continued), Senegal, and Togo.* 11

Table S4. *Subnational estimates of percent reach and per capita daily consumption (PCDC) in grams per day per adult female equivalent of wheat flour, edible oil, rice, maize, millet and sorghum by socioeconomic wealth quintile across ten countries in the West Africa region.* *12*

Table S5. *Sensitivity analysis of public health potential of staple grains based on the beneficiary population size and characteristics of vehicle consumption amongst potential beneficiaries assuming a higher threshold (MPI > 0.75) to define beneficiaries* *13*

Table S6. *Sensitivity analysis of the public health potential of wheat flour, rice and maize assuming addition of vitamin A, based on the beneficiary population size and characteristics of vehicle consumption amongst potential beneficiaries.* *15*

Table S7. *Sensitivity analysis of vulnerability to inadequate iron and zinc intake assuming bioavailability of 15% for iron (REF WHO 2006) and a refined diet for zinc (REF Allen et al., 2020)* *16*

Table S8. *Summary of national micronutrient survey results from Ghana, Nigeria, and Senegal reporting the prevalence of deficiencies in vitamin A and zinc among children aged 12–59 months and folate, iron, and anaemia among women of reproductive age, along with designations used in global guidance to determine when deficiency prevalence constitutes a public health problem requiring population intervention.* *17*

Table S9. *Summary of per capita daily consumption estimates of staple foods from this study using Household Consumption & Expenditure surveys (HCES) compared to per capita daily availability (PCDA) estimates using the Food and Agriculture Organization Supply Utilisation Accounts (SUA).* *18*

Table S10. *Included countries and the corresponding Household Consumption and Expenditure Survey (HCES) used in this study* *19*

Table S11. *Food items in each Household Consumption and Expenditure Survey classified as or containing fortifiable staple grains and industrially produced edible oils.* *20*

Table S12. *Fortification recommendations for wheat flour, edible oil, rice, sorghum, millet, and maize presented in global normative guidance and experimental research* *22*

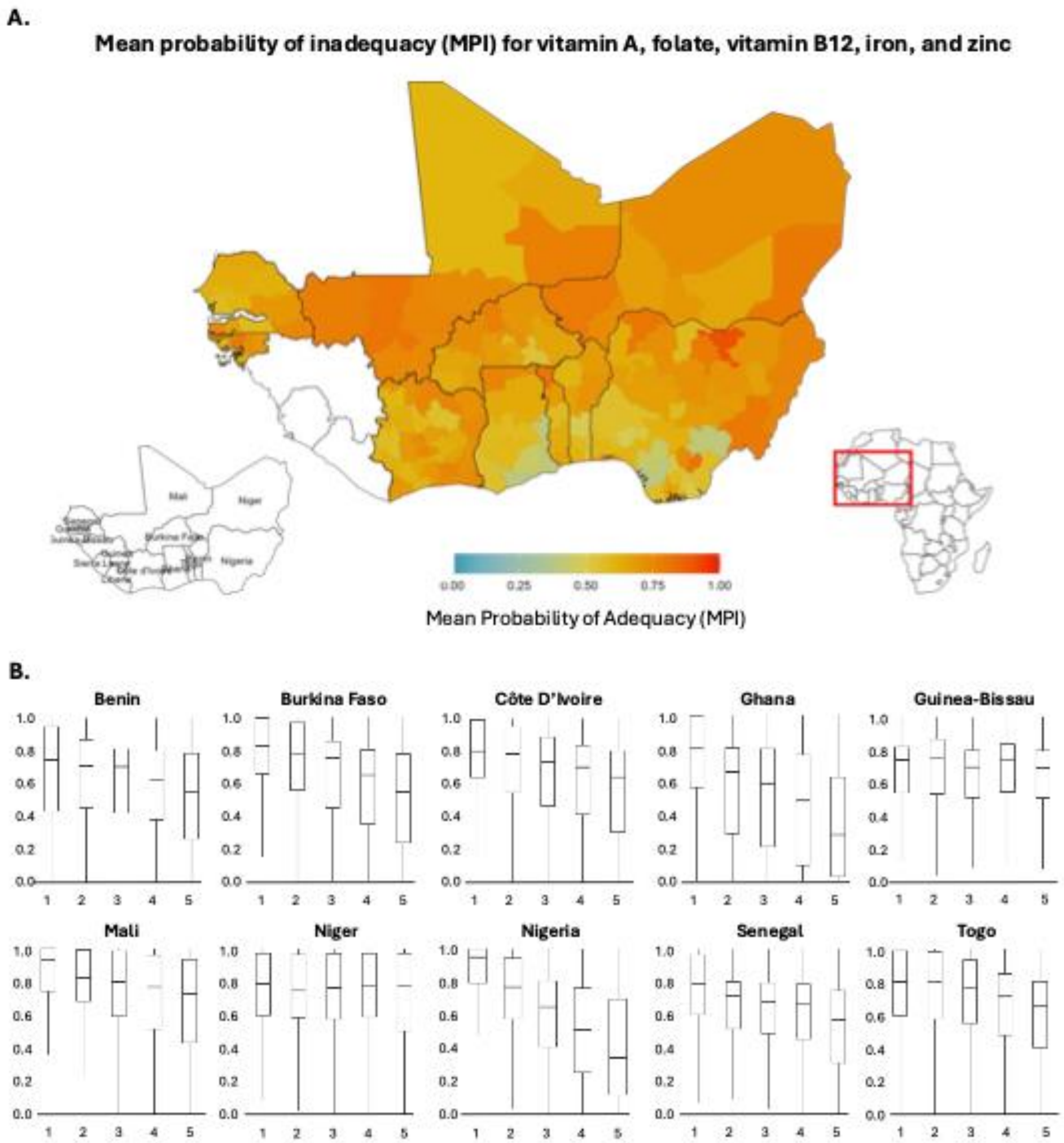

Footnote. Mean probability of adequacy (MPI) were estimated using the Enquête Harmonisée sur les Conditions de Vie des Ménages (EHCVM, 2021/2022), the Ghana Living Standards Survey (2016/2017) and Nigeria Living Standards Survey (2018/2019). MPI was estimated by combining the simple average of the probability of inadequacy for all five micronutrients. The daily requirements distributions were adjusted to assume low bioavailability for iron and zinc based on a diet of unrefined grains (REF Allen 2006 and Allen 2020)

Figure S1. *Subnational assessment of vulnerability to inadequate micronutrient intake by household mean probabilities of inadequacy (MPI) for vitamin A, folate, vitamin B12, iron, and zinc in ten West African countries, shown by (A) geography by first administrative units (ADM1) and (B) quintile of socioeconomic position (poorest = 1 to wealthiest = 5).*

Table S1. Subnational-level vulnerability to inadequate micronutrient intake by administrative level 1 in Benin, Burkina Faso, and Cote D'Ivoire, including the mean probability of inadequacy (MPI) and the percentage of the population vulnerable to inadequate intake for vitamin A, folate, vitamin B12, iron, and zinc.

			**Percent vulnerable to inadequate intake**				
Country	ADM 1	Overall MPI[1]	Vitamin A (%)	Folate (%)	Vitamin B12 (%)	Iron (%)	Zinc (%)
Benin	Alibori	0.69	83	62	77	54	70
	Atacora	0.59	84	47	72	41	59
	Atlantique	0.61	44	64	58	59	84
	Borgou	0.71	80	64	76	62	76
	Collines	0.52	18	43	71	51	64
	Couffo	0.61	40	62	69	57	81
	Donga	0.66	55	66	76	63	74
	Littoral	0.67	47	74	56	72	86
	Mono	0.61	39	70	49	62	84
	Oueme	0.52	23	58	42	55	80
	Plateau	0.54	24	56	55	55	84
	Zou	0.58	20	58	72	55	86
Burkina Faso	Boucle du Mouhoum	0.69	91	72	62	62	79
	Cascades	0.61	97	66	24	55	75
	Centre	0.59	93	52	16	54	74
	Centre-Est	0.54	87	54	24	45	60
	Centre-Nord	0.68	97	71	56	54	79
	Centre-Ouest	0.67	98	66	50	59	74
	Centre-Sud	0.67	95	72	27	62	78
	Est	0.63	77	66	64	53	73
	Hauts-Bassins	0.64	97	65	30	58	79
	Nord	0.75	99	81	56	64	90
	Plateau-Central	0.68	99	74	42	58	87
	Sahel	0.65	99	53	81	47	74
	Sud-Ouest	0.64	94	61	53	58	68
Cote D'Ivoire	Agneby-Tiassa	0.74	76	93	43	78	89
	Autonome d'Abidjan	0.72	74	88	38	80	84
	Bafing	0.63	45	87	35	73	80
	Bagoue	0.71	82	81	39	74	84
	Belier	0.62	76	79	27	54	75
	Bere	0.6	52	69	39	60	77
	Bounkani	0.73	89	80	51	64	79
	Cavally	0.61	74	71	39	53	75
	Folon	0.70	87	80	43	72	76
	Gbeke	0.68	79	83	35	66	80
	Gbokle	0.6	72	72	38	51	72
	Goh	0.65	79	76	37	60	78
	Gontougo	0.74	93	88	39	69	85
	Grands-Ponts	0.74	69	92	38	82	87
	Guemon	0.54	64	63	38	48	67
	Hambol	0.54	74	67	27	46	63
	Haut-Sassandra	0.67	78	76	54	57	80
	Iffou	0.65	87	73	39	53	67
	Indenie-Djuablin	0.71	86	83	36	69	81
	Kabadougou	0.60	62	75	33	62	68
	La Me	0.74	75	87	40	79	85
	Loh-Djiboua	0.70	74	82	40	68	87
	Marahoue	0.74	87	82	51	68	86
	Moronou	0.66	81	84	30	64	78
	N'Zi	0.61	75	75	35	54	67
	Nawa	0.60	70	69	38	55	72
	Poro	0.74	85	85	46	76	85
	San Pedro	0.70	78	83	44	64	79
	Sud-Comoe	0.74	78	92	41	74	89
	Tchologo	0.76	89	85	48	76	86
	Tonkpi	0.58	47	77	31	64	75
	Worodougou	0.62	50	74	44	67	78
	Yamoussoukro	0.70	72	88	35	73	90

Table S1 (continued). Subnational-level vulnerability to inadequate micronutrient intake by administrative level 1 in Ghana, Guinea-Bissau, Mali, Niger, and Nigeria, including the mean probability of inadequacy (MPI) and the percentage of population vulnerable to inadequate intake for vitamin A, folate, vitamin B12, iron, and zinc.

			Percent vulnerable to inadequate intake				
Country	ADM 1	Overall MPI[1]	Vitamin A (%)	Folate (%)	Vitamin B12 (%)	Iron (%)	Zinc (%)
Ghana	Ashanti	0.57	57	75	25	67	70
	Brong Ahafo	0.57	66	69	21	65	67
	Central	0.41	29	56	10	53	53
	Eastern	0.47	39	59	12	59	59
	Greater Accra	0.41	40	49	15	47	53
	Northern	0.64	88	58	43	63	67
	Upper East	0.75	86	65	73	72	78
	Upper West	0.76	96	72	62	74	77
	Volta	0.42	40	44	14	53	56
	Western	0.55	57	79	11	67	64
Guinea-Bissau	Bafata	0.73	55	85	69	73	82
	Biombo	0.69	49	93	33	82	86
	Bolama Bijagos	0.52	31	90	5	60	74
	Cacheu	0.61	47	84	33	68	75
	Gabu	0.7	35	81	77	75	82
	Oio	0.81	61	96	70	86	92
	Quinara	0.67	45	93	37	77	86
	SAB	0.62	41	87	25	76	79
	Tombali	0.61	48	88	33	64	75
Mali	Bamako	0.75	66	88	42	85	91
	Gao	0.78	89	87	46	83	81
	Kayes	0.76	92	78	71	69	84
	Kidal	0.63	83	66	28	79	69
	Koulikoro	0.81	86	80	81	75	88
	Mopti	0.70	96	71	55	68	80
	Segou	0.75	92	63	76	67	84
	Sikasso	0.74	89	74	62	70	85
	Tombouctou	0.56	75	69	24	60	69
Niger	Agadez	0.71	91	43	86	64	82
	Diffa	0.80	98	56	86	72	91
	Dosso	0.74	96	48	91	56	81
	Maradi	0.71	97	36	95	45	81
	Niamey	0.75	82	71	72	73	84
	Tahoua	0.70	98	34	93	46	74
	Tillaberi	0.78	97	60	91	65	87
	Zinder	0.67	94	31	93	41	72
Nigeria	Abia	0.52	72	55	44	46	51
	Abuja	0.57	94	45	38	48	62
	Adamawa	0.75	88	77	62	71	83
	Akwa Ibom	0.49	71	58	24	48	54
	Anambra	0.49	68	56	25	48	60
	Bauchi	0.70	90	56	80	52	79
	Bayelsa	0.53	71	61	18	54	76
	Benue	0.41	76	23	41	27	42
	Borno	0.72	91	72	61	66	89
	Cross River	0.55	79	64	21	52	63
	Delta	0.38	51	43	9	39	55
	Ebonyi	0.81	87	76	70	79	96
	Edo	0.44	58	49	18	41	58
	Ekiti	0.51	73	35	43	41	74
	Enugu	0.72	88	75	42	74	88
	Gombe	0.67	91	44	84	48	71
	Imo	0.57	78	63	38	55	58
	Jigawa	0.90	100	95	82	84	95
	Kaduna	0.71	90	56	69	63	81
	Kano	0.80	99	65	81	67	88
	Katsina	0.58	97	35	56	36	69
	Kebbi	0.62	81	58	57	43	76
	Kogi	0.60	86	43	44	51	84
	Kwara	0.56	85	40	36	45	76

Table S1 (continued). Subnational-level vulnerability to inadequate micronutrient intake by administrative level 1 in Nigeria (continued), Senegal, and Togo, including the mean probability of inadequacy (MPI) and the percentage of the population vulnerable to inadequate intake for vitamin A, folate, vitamin B12, iron, and zinc.

			Percent vulnerable to inadequate intake				
Country	ADM 1	Overall MPI[1]	Vitamin A (%)	Folate (%)	Vitamin B12 (%)	Iron (%)	Zinc (%)
Nigeria	Lagos	0.71	89	68	46	73	83
	Nassarawa	0.67	95	54	57	58	77
	Niger	0.68	89	64	53	59	76
	Ogun	0.58	83	48	33	58	77
	Ondo	0.59	88	50	26	56	82
	Osun	0.48	73	40	22	46	65
	Oyo	0.53	74	46	37	48	71
	Plateau	0.69	81	72	47	65	84
	Rivers	0.65	82	64	39	64	85
	Sokoto	0.81	98	67	91	61	88
	Taraba	0.78	92	73	81	72	83
	Yobe	0.69	93	58	61	56	84
	Zamfara	0.73	98	74	56	58	82
Senegal	Dakar	0.61	53	90	18	72	75
	Diourbel	0.59	64	87	7	67	81
	Fatick	0.59	56	74	14	67	79
	Kaffrine	0.61	79	70	16	67	77
	Kaolack	0.57	62	75	7	66	77
	Kedougou	0.72	61	83	65	77	88
	Kolda	0.58	38	76	28	69	80
	Louga	0.61	76	84	10	68	81
	Matam	0.63	75	77	23	65	74
	Saint-Louis	0.64	69	89	12	73	84
	Sedhiou	0.68	54	85	37	79	86
	Tambacounda	0.69	80	77	41	73	81
	Thies	0.62	62	89	9	70	82
	Ziguinchor	0.74	57	96	38	89	95
Togo	Centrale	0.56	55	52	60	51	68
	Grand Lome	0.72	49	87	50	83	94
	Kara	0.71	61	71	62	72	86
	Maritime	0.68	47	83	42	76	89
	Plateaux	0.62	49	68	61	58	84
	Savanes	0.82	89	78	74	76	91

Footnote. Mean probability of adequacy (MPI) and percentage vulnerable to inadequate intake were estimated using the Enquête Harmonisée sur les Conditions de Vie des Ménages (EHCVM, 2021/2022), the Ghana Living Standards Survey (2016/2017) and Nigeria Living Standards Survey (2018/2019). MPI was estimated by combining the simple average of the probability of inadequacy for all five micronutrients. For vitamin A, folate, vitamin B12, and zinc percent vulnerable to inadequate intake was estimated using the cut-point approach, where apparent intakes lower than the Harmonized Average Requirement of a non-pregnant, non-lactating woman 18-29-year-old woman, were identified as vulnerable to inadequate intake. The percent vulnerable to iron inadequacy was estimated using the full probability approach. The daily requirements distributions were adjusted to assume low bioavailability for iron and zinc based on a diet of unrefined grains (REF Allen 2006 and Allen 2020)

Table S2. Subnational-level vulnerability to inadequate micronutrient intake by socioeconomic wealth quintile in ten countries in the West African region, including the mean probability of inadequacy (MPI) and the percentage of the population vulnerable to inadequate intake for vitamin A, folate, vitamin B12, iron, and zinc.

			**Percent vulnerable to inadequate intake**				
Country	Socioeconomic position (by quintile)	Overall MPI[1]	Vitamin A (%)	Folate (%)	Vitamin B12 (%)	Iron (%)	Zinc (%)
Benin	1 (poorest)	0.65	52	67	68	63	82
	2	0.64	49	63	68	60	82
	3	0.63	46	63	68	59	81
	4	0.58	42	58	62	56	76
	5 (wealthiest)	0.52	40	53	49	51	71
Burkina Faso	1 (poorest)	0.77	96	79	62	66	85
	2	0.72	96	71	50	63	83
	3	0.67	95	68	42	57	77
	4	0.59	92	56	33	51	69
	5 (wealthiest)	0.53	88	47	22	45	64
Cote D'Ivoire	1 (poorest)	0.76	81	88	53	74	89
	2	0.71	78	83	44	67	82
	3	0.69	77	82	39	63	81
	4	0.66	73	79	36	62	77
	5 (wealthiest)	0.61	65	79	26	61	74
Ghana	1 (poorest)	0.70	73	79	41	77	81
	2	0.59	61	70	27	65	70
	3	0.52	54	64	19	61	63
	4	0.43	44	56	13	52	55
	5 (wealthiest)	0.35	34	47	7	43	44
Guinea-Bissau	1 (poorest)	0.69	51	88	53	72	82
	2	0.69	49	89	50	74	82
	3	0.65	43	86	43	72	80
	4	0.67	47	88	40	77	82
	5 (wealthiest)	0.64	41	88	35	76	80
Mali	1 (poorest)	0.83	95	81	73	80	92
	2	0.79	93	77	66	76	88
	3	0.76	91	73	64	73	83
	4	0.71	84	72	55	70	79
	5 (wealthiest)	0.67	71	74	47	68	77
Niger	1 (poorest)	0.75	98	46	97	54	82
	2	0.72	97	41	94	52	81
	3	0.72	97	44	94	53	78
	4	0.70	97	37	92	57	79
	5 (wealthiest)	0.68	88	48	77	60	76
Nigeria	1 (poorest)	0.87	97	87	81	79	93
	2	0.74	94	69	64	62	85
	3	0.64	88	57	45	53	76
	4	0.55	80	47	33	45	70
	5 (wealthiest)	0.46	65	39	25	38	58
Senegal	1 (poorest)	0.73	75	86	39	81	89
	2	0.64	66	84	19	73	83
	3	0.63	61	85	16	71	82
	4	0.59	55	87	9	69	78
	5 (wealthiest)	0.52	47	83	5	60	66
Togo	1 (poorest)	0.72	64	76	67	72	87
	2	0.72	61	77	62	72	89
	3	0.70	55	78	56	71	88
	4	0.67	51	76	50	68	87
	5 (wealthiest)	0.63	44	75	44	65	86

Footnote. Mean probability of adequacy (MPI) and percentage vulnerable to inadequate intake were estimated using the Enquête Harmonisée sur les Conditions de Vie des Ménages (EHCVM, 2021/2022), the Ghana Living Standards Survey (2016/2017) and Nigeria Living Standards Survey (2018/2019). MPI was estimated by combining the simple average of the probability of inadequacy for all five micronutrients. For vitamin A, folate, vitamin B12, and zinc percent vulnerable to inadequate intake was estimated using the cut-point approach, where apparent

intakes lower than the Harmonized Average Requirement of a non-pregnant, non-lactating woman 18-29-year-old woman, were identified as vulnerable to inadequate intake. The percent vulnerable to iron inadequacy was estimated using the full probability approach. The daily requirements distributions were adjusted to assume low bioavailability for iron and zinc based on a diet of unrefined grains (REF Allen 2006 and Allen 2020)

Table S3. Subnational estimates of percent reach and per capita daily consumption (PCDC) in grams per day per adult female equivalent of wheat flour, edible oil, rice, maize, millet and sorghum at administrative level one in Benin, Burkina Faso, and Cote D'Ivoire.

		Wheat flour		Edible oil		Rice		Maize		Millet		Sorghum	
Country	ADM 1	Reach (%)	PCDC (g/day/AFE)	Reach (%)	PCDC (g/day/AFE)	Reach (%)	PCDC (g/day/AFE)	Reach (%)	PCDC (g/day/AFE)	Reach (%)	PCDC (g/day/AFE)	Reach (%)	PCDC (g/day/AFE)
Benin	Alibori	51	7	6	31	89	92	90	338	12	105	57	148
	Atacora	47	11	9	31	84	97	93	463	8	193	44	205
	Atlantique	73	11	44	32	93	109	94	183	2	53	2	52
	Borgou	35	6	10	50	76	92	91	273	9	50	29	120
	Collines	90	9	31	33	94	79	93	218	6	43	5	138
	Couffo	63	6	54	31	79	72	95	247	1	41	1	275
	Donga	54	5	19	58	81	76	91	239	10	63	15	98
	Littoral	74	12	61	32	89	86	82	132	7	41	2	97
	Mono	50	8	47	38	79	85	79	275	1	19	0	0
	Oueme	93	9	54	33	95	74	93	157	2	40	1	41
	Plateau	80	7	6	26	90	69	96	213	1	19	1	210
	Zou	72	9	56	26	95	83	96	189	1	21	1	25
Burkina Faso	Boucle du Mouhoum	35	10	49	10	48	94	70	268	10	168	16	240
	Cascades	71	10	30	17	63	131	87	176	2	64	2	46
	Centre	74	10	71	17	93	112	85	108	1	34	1	126
	Centre-Est	63	8	68	18	75	106	84	186	3	72	10	154
	Centre-Nord	40	7	71	11	41	79	35	176	20	156	64	280
	Centre-Ouest	49	6	58	12	68	86	59	207	28	140	41	159
	Centre-Sud	36	5	62	13	77	95	92	203	4	62	8	96
	Est	13	6	32	10	58	70	30	175	15	184	41	236
	Hauts-Bassins	49	15	36	16	67	92	92	221	2	115	2	315
	Nord	31	6	73	9	53	71	49	196	11	37	50	240
	Plateau-Central	39	3	70	13	66	72	47	186	6	74	29	179
	Sahel	19	8	51	12	37	82	10	176	77	421	39	407
	Sud-Ouest	42	7	42	13	61	83	95	298	7	102	8	174
Cote D'Ivoire	Agneby-Tiassa	61	9	81	7	71	144	15	51	2	38	0	0
	Autonome d'Abidjan	84	11	89	11	73	154	18	36	2	39	1	7
	Bafing	65	16	79	9	96	188	45	55	2	45	0	0
	Bagoue	67	11	90	10	93	203	81	99	1	154	0	0
	Belier	84	8	92	10	73	158	16	44	2	20	0	0
	Bere	53	13	88	8	96	254	65	74	1	36	0	0
	Bounkani	60	12	84	7	88	129	82	75	2	20	3	50
	Cavally	53	7	69	10	88	213	44	59	2	26	1	7
	Folon	64	10	81	12	96	219	89	97	4	39	2	101
	Gbeke	79	11	93	12	83	159	23	57	4	47	0	36
	Gbokle	67	13	83	12	83	198	40	67	2	26	0	0
	Goh	73	10	80	8	91	186	38	56	2	91	0	78
	Gontougo	70	8	91	8	77	137	36	58	2	38	1	57
	Grands-Ponts	82	9	89	10	79	150	25	56	2	28	1	19
	Guemon	71	7	76	10	91	190	38	71	3	34	1	4
	Hambol	85	10	95	10	90	189	73	71	2	34	0	0
	Haut-Sassandra	58	11	80	11	85	172	40	67	1	70	0	0
	Iffou	76	9	94	10	86	145	15	60	0	0	0	0
	Indenie-Djuablin	72	9	89	10	82	147	41	58	1	33	1	28
	Kabadougou	63	12	88	10	97	244	77	65	4	23	1	28
	La Me	67	9	85	9	71	158	19	69	2	53	0	0
	Loh-Djiboua	75	9	82	8	81	145	42	61	3	35	1	87
	Marahoue	53	8	84	8	86	175	35	64	1	172	0	0
	Moronou	78	11	90	9	68	142	9	52	1	44	0	0
	N'Zi	62	8	87	9	76	147	12	42	1	25	0	0
	Nawa	71	9	83	10	87	224	37	68	3	42	1	98
	Poro	75	13	90	10	93	207	77	72	2	36	1	27
	San Pedro	68	10	81	10	80	195	27	52	3	78	0	0
	Sud-Comoe	72	11	92	8	82	168	24	53	4	29	0	0
	Tchologo	79	12	89	10	91	167	80	76	4	34	3	46
	Tonkpi	57	8	61	9	92	179	20	47	2	34	1	27
	Worodougou	64	12	81	9	92	227	74	67	2	47	1	29
	Yamoussoukro	73	8	91	10	68	143	19	38	5	34	1	56

Table S3 (continued). Subnational estimates of percent reach and per capita daily consumption (PCDC) in grams per day per adult female equivalent of wheat flour, edible oil, rice, maize, millet and sorghum at administrative level one in Ghana, Guinea-Bissau, Mali Niger, and Nigeria.

		Wheat flour		Edible oil		Rice		Maize		Millet		Sorghum	
Country	ADM 1	Reach (%)	PCDC (g/day/AFE)	Reach (%)	PCDC (g/day/AFE)	Reach (%)	PCDC (g/day/AFE)	Reach (%)	PCDC (g/day/AFE)	Reach (%)	PCDC (g/day/AFE)	Reach (%)	PCDC (g/day/AFE)
Ghana	Ashanti	26	11	62	17	78	90	65	116	2	24	1	43
	Brong Ahafo	7	2	64	14	70	72	52	261	1	40	0	0
	Central	23	4	79	14	91	98	77	179	1	0	1	54
	Eastern	17	3	68	17	81	94	62	168	1	42	1	20
	Greater Accra	36	8	55	18	75	109	79	169	1	35	1	43
	Northern	8	1	78	15	71	114	45	455	8	50	8	98
	Upper East	8	1	71	12	69	75	46	387	16	94	7	123
	Upper West	3	2	34	15	55	75	38	439	9	88	6	154
	Volta	13	2	71	12	79	56	75	255	1	33	1	37
	Western	13	5	63	18	81	89	47	138	1	0	0	0
Guinea-Bissau	Bafata	69	7	68	15	99	290	11	95	11	69	8	88
	Biombo	70	7	83	19	99	299	1	37	1	38	0	0
	Bolama Bijagos	54	8	66	37	100	305	2	40	1	96	1	278
	Cacheu	71	11	67	31	99	294	1	102	2	55	1	98
	Gabu	72	7	52	15	97	245	13	100	14	87	13	108
	Oio	61	5	67	24	99	289	3	58	4	87	2	92
	Quinara	65	7	48	21	100	292	1	65	1	102	1	55
	SAB	92	9	74	36	98	258	3	71	3	60	0	0
	Tombali	64	8	55	16	99	330	2	57	1	30	1	45
Mali	Bamako	93	14	95	22	99	56	29	48	59	58	12	49
	Gao	31	16	85	30	98	308	4	131	44	153	4	134
	Kayes	76	16	74	16	91	169	55	162	18	88	49	157
	Kidal	27	47	89	31	100	115	1	84	2	132	1	27
	Koulikoro	65	8	63	23	85	168	36	163	50	104	33	161
	Mopti	55	7	72	17	95	195	2	137	88	204	17	53
	Segou	64	8	70	14	78	173	21	171	76	217	17	131
	Sikasso	67	9	44	17	86	122	85	245	27	103	17	128
	Tombouctou	48	26	53	25	100	224	3	113	80	148	5	68
Niger	Agadez	45	16	98	27	98	194	24	113	87	201	5	154
	Diffa	27	10	88	23	90	137	35	137	88	232	18	189
	Dosso	13	10	66	16	72	139	72	305	90	320	16	207
	Maradi	20	7	73	12	68	103	36	192	95	362	56	225
	Niamey	39	15	85	29	96	242	59	142	35	97	4	78
	Tahoua	15	6	87	16	72	128	13	187	95	402	52	284
	Tillaberi	25	5	87	12	74	163	58	303	85	268	11	187
	Zinder	39	9	72	19	78	124	34	211	88	316	57	294
Nigeria	Abia	87	18	84	9	95	62	15	40	1	29	2	38
	Abuja	94	14	86	11	79	77	88	114	10	79	55	111
	Adamawa	72	15	42	7	93	56	1	60	2	14	15	14
	Akwa Ibom	91	7	82	14	97	76	9	39	1	37	1	32
	Anambra	95	7	74	9	70	90	54	158	60	122	65	212
	Bauchi	69	22	48	12	80	48	1	83	1	7	1	17
	Bayelsa	92	8	45	11	76	74	17	105	5	29	28	98
	Benue	38	9	87	12	87	66	75	89	33	55	50	114
	Borno	71	17	49	12	97	58	2	47	1	14	2	24
	Cross River	78	10	75	14	90	72	2	70	1	19	1	48
	Delta	92	26	36	8	96	63	1	71	0	0	0	0
	Ebonyi	40	14	87	12	100	59	22	42	1	26	4	38
	Edo	85	11	32	14	93	74	14	57	1	23	15	22
	Ekiti	79	29	46	11	96	51	22	42	1	76	1	50
	Enugu	74	15	66	11	96	59	75	61	35	50	67	72
	Gombe	71	22	70	8	76	129	73	205	53	124	57	253
	Imo	87	21	86	13	94	67	23	18	1	58	1	60
	Jigawa	41	20	79	13	76	70	41	81	71	79	67	148
	Kaduna	64	23	86	17	93	75	93	107	29	42	62	78
	Kano	68	35	83	14	88	122	63	138	58	68	58	178
	Katsina	77	26	73	16	73	76	89	177	75	128	74	256
	Kebbi	64	21	50	10	71	136	66	176	79	166	61	226
	Kogi	92	7	74	8	97	60	65	44	20	22	49	38
	Kwara	76	30	42	7	87	92	49	77	6	49	71	112

Table S3 (continued). Subnational estimates of percent reach and per capita daily consumption (PCDC) in grams per day per adult female equivalent of wheat flour, edible oil, rice, maize, millet and sorghum at administrative level one in Nigeria (continued), Senegal, and Togo.

		Wheat flour		Edible oil		Rice		Maize		Millet		Sorghum	
Country	ADM 1	Reach (%)	PCDC (g/day/AFE)	Reach (%)	PCDC (g/day/AFE)	Reach (%)	PCDC (g/day/AFE)	Reach (%)	PCDC (g/day/AFE)	Reach (%)	PCDC (g/day/AFE)	Reach (%)	PCDC (g/day/AFE)
Nigeria (continued)	Lagos	97	22	81	16	93	45	23	71	2	40	3	30
	Nassarawa	79	12	57	10	96	94	87	113	22	60	60	87
	Niger	80	18	62	11	94	100	81	149	63	69	70	125
	Ogun	93	25	77	11	97	85	55	50	0	14	2	41
	Ondo	79	37	39	11	91	74	11	34	1	35	3	36
	Osun	82	27	58	10	95	71	65	56	2	33	21	41
	Oyo	85	22	77	14	87	78	60	47	4	30	11	51
	Plateau	43	27	57	10	89	61	91	98	32	36	73	58
	Rivers	84	17	55	11	86	104	1	44	2	102	2	54
	Sokoto	29	22	68	12	73	97	39	100	96	201	85	177
	Taraba	30	19	30	8	60	59	89	136	4	32	28	103
	Yobe	72	24	85	13	78	86	28	128	75	133	50	237
	Zamfara	45	17	72	11	75	113	54	96	83	120	85	210
Senegal	Dakar	89	40	83	49	85	183	10	23	21	26	1	5
	Diourbel	94	38	70	58	94	216	9	61	42	113	1	24
	Fatick	83	20	85	45	97	213	48	83	77	135	1	22
	Kaffrine	68	29	89	42	99	238	43	156	73	172	1	112
	Kaolack	83	20	85	47	96	219	46	95	61	110	1	20
	Kedougou	52	37	57	24	89	227	60	114	15	67	2	103
	Kolda	60	17	79	17	95	221	51	140	38	102	14	94
	Louga	82	29	85	63	97	274	16	83	52	66	0	0
	Matam	87	41	90	56	98	252	29	105	48	128	8	63
	Saint-Louis	90	38	86	69	96	188	10	66	50	39	3	57
	Sedhiou	57	19	90	20	98	291	20	109	58	119	0	0
	Tambacounda	71	18	71	26	95	214	48	110	58	134	11	81
	Thies	91	38	91	50	95	218	10	35	38	66	1	242
	Ziguinchor	78	34	65	29	98	218	2	48	30	46	1	46
Togo	Centrale	9	12	56	20	65	108	97	340	1	151	2	185
	Grand Lome	33	9	84	15	83	57	86	107	1	45	1	68
	Kara	17	7	55	12	66	57	95	220	1	66	2	164
	Maritime	16	11	60	10	63	67	96	225	1	243	1	110
	Plateaux	16	10	71	17	74	93	97	266	1	114	2	153
	Savanes	14	6	35	20	68	91	96	317	5	76	3	286

Footnote. Reach is defined as the percentage of households that consume any amount of the staple. Per capita daily consumption (PCDC) is defined grams/day per AFE of the staple consumed, among consumers. For wheat flour and edible oil, reach and per capita daily consumption reflect current programme reach of the fortifiable (market-acquired industrially processed) form. For rice, maize, millet, and sorghum, they reflect total current consumption regardless of form, serving as a demand signal for prospective fortification.

Table S4. Subnational estimates of percent reach and per capita daily consumption (PCDC) in grams per day per adult female equivalent of wheat flour, edible oil, rice, maize, millet and sorghum by socioeconomic wealth quintile across ten countries in the West Africa region.

		Wheat flour		Edible oil		Rice		Maize		Millet		Sorghum	
Country	ADM 1	Reach (%)	PCDC (g/day/AFE)	Reach (%)	PCDC (g/day/AFE)	Reach (%)	PCDC (g/day/AFE)	Reach (%)	PCDC (g/day/AFE)	Reach (%)	PCDC (g/day/AFE)	Reach (%)	PCDC (g/day/AFE)
Benin	1 (poorest)	51	9	28	37	72	77	87	191	3	78	9	101
	2	60	8	32	31	86	82	91	180	3	72	12	140
	3	67	8	39	31	91	84	93	181	3	56	10	149
	4	74	9	41	31	94	89	93	178	6	54	12	148
	5 (wealthiest)	83	10	41	32	97	89	94	168	7	57	14	186
Burkina Faso	1 (poorest)	20	7	44	10	45	82	47	218	16	184	37	241
	2	35	6	54	11	60	90	66	169	15	187	22	208
	3	45	8	55	14	64	96	66	170	11	173	22	250
	4	58	8	60	15	74	96	70	138	13	156	19	211
	5 (wealthiest)	75	10	69	15	84	104	82	94	7	144	12	232
Cote D'Ivoire	1 (poorest)	52	12	73	9	80	180	28	59	1	40	1	37
	2	62	10	82	9	83	172	35	55	1	38	1	12
	3	73	10	86	9	83	173	39	46	2	47	1	37
	4	84	10	90	11	81	172	36	44	3	42	1	36
	5 (wealthiest)	89	9	94	11	82	161	31	34	5	36	1	46
Ghana	1 (poorest)	8	4	49	11	60	61	42	120	2	72	1	91
	2	13	4	62	14	75	77	56	114	2	32	1	94
	3	19	9	66	15	79	89	63	115	2	53	1	78
	4	25	6	72	17	86	97	74	117	2	42	1	55
	5 (wealthiest)	36	7	72	20	86	112	83	110	3	16	1	119
Guinea-Bissau	1 (poorest)	49	11	57	22	97	320	3	76	4	99	4	115
	2	68	9	67	22	99	297	4	64	4	96	4	117
	3	78	8	65	22	99	288	5	61	5	70	3	85
	4	86	7	67	27	99	266	4	85	6	55	2	63
	5 (wealthiest)	92	8	78	30	99	238	6	46	4	60	1	94
Mali	1 (poorest)	45	8	53	15	81	134	21	172	60	187	25	124
	2	58	9	64	18	87	161	31	173	53	165	25	116
	3	63	10	67	20	90	165	35	139	53	158	23	174
	4	75	12	74	19	93	167	40	133	49	131	21	150
	5 (wealthiest)	88	13	88	20	99	100	39	73	58	82	16	62
Niger	1 (poorest)	14	6	71	15	58	105	30	253	91	344	36	329
	2	21	5	78	14	65	121	32	230	92	351	42	295
	3	21	7	79	14	75	140	39	221	90	335	38	263
	4	27	8	84	16	85	139	39	194	89	339	42	245
	5 (wealthiest)	43	12	83	21	95	187	59	157	74	211	29	197
Nigeria	1 (poorest)	42	11	53	9	71	55	51	88	44	96	55	137
	2	68	15	64	10	87	71	49	92	33	102	45	136
	3	81	17	68	11	93	72	44	72	20	84	31	116
	4	87	20	72	12	95	75	37	57	12	72	20	88
	5 (wealthiest)	91	28	73	18	90	77	29	59	6	58	10	77
Senegal	1 (poorest)	64	30	67	35	81	237	19	91	37	144	3	116
	2	80	31	80	44	91	233	23	67	42	93	1	103
	3	87	32	82	46	94	218	21	67	42	100	1	112
	4	93	36	90	49	98	205	20	44	42	58	1	65
	5 (wealthiest)	97	40	92	53	99	185	19	22	39	38	1	55
Togo	1 (poorest)	7	13	40	17	45	85	94	260	1	86	1	285
	2	11	11	61	16	66	81	91	217	1	172	1	216
	3	17	8	69	14	76	67	92	186	1	56	1	153
	4	25	8	76	15	84	69	95	160	1	54	1	173
	5 (wealthiest)	43	9	83	16	92	65	93	116	1	66	2	68

Footnote. Reach is defined as the percentage of households that consume any amount of the staple. Per capita daily consumption (PCDC) is defined grams/day per AFE of the staple consumed, among consumers. For wheat flour and edible oil, reach and per capita daily consumption reflect current programme reach of the fortifiable (market-acquired industrially processed) form. For rice, maize, millet, and sorghum, they reflect total current consumption regardless of form, serving as a demand signal for prospective fortification.

Table S5. *Sensitivity analysis of public health potential of staple grains based on the beneficiary population size and characteristics of vehicle consumption amongst potential beneficiaries assuming a higher threshold (MPI > 0.75) to define beneficiaries*

Country	Vehicle	Beneficiary population[1] national, %	Of the beneficiary[1] population: Coverage[2], %	Median (IQR) PCDC	Consumption category[3]
Benin	Wheat flour	46	61	12 (5, 22)	Low
	Rice	42	85	70 (47, 103)	Low
	Millet	48	4	46 (25, 69)	Low
	Sorghum	48	11	101 (66, 164)	Mid-low
	Maize	46	89	149 (96, 221)	Mid-low
Burkina Faso	Wheat flour	36	28	7 (4, 15)	Low
	Rice	31	45	65 (40, 107)	Low
	Millet	35	13	152 (76, 241)	Mid-high
	Sorghum	35	35	238 (142, 396)	Mid-high
	Maize	36	55	180 (97, 290)	Mid-high
Cote D'Ivoire	Wheat flour	41	65	14 (8, 22)	Low
	Rice	35	76	154 (103, 221)	Mid-high
	Millet	38	1	34 (20, 57)	Low
	Sorghum	38	0	27 (10, 46)	Low
	Maize	41	29	42 (23, 70)	Low
Ghana	Wheat flour	24	16	6 (1, 19)	Low
	Rice	19	45	54 (30, 103)	Low
	Millet	19	2	34 (16, 61)	Low
	Sorghum	19	1	42 (30, 104)	Low
	Maize	24	31	57 (32, 97)	Low
Guinea-Bissau	Wheat flour	47	67	14 (7, 25)	Low
	Rice	42	98	276 (208, 363)	Mid-high
	Millet	43	6	70 (38, 123)	Low
	Sorghum	43	4	94 (51, 125)	Mid-low
	Maize	47	4	56 (31, 97)	Low
Mali	Wheat flour	53	65	14 (8, 26)	Low
	Rice	49	90	142 (74, 221)	Mid-low
	Millet	52	49	117 (66, 194)	Mid-low
	Sorghum	52	25	113 (60, 210)	Mid-low
	Maize	53	36	122 (71, 202)	Mid-low
Niger	Wheat flour	42	21	9 (4, 18)	Low
	Rice	37	84	145 (84, 236)	Mid-low
	Millet	46	80	211 (132, 298)	Mid-high
	Sorghum	46	28	193 (119, 288)	Mid-high
	Maize	42	47	172 (101, 267)	Mid-high
Nigeria	Wheat flour	36	67	24 (15, 43)	Low
	Rice	32	82	61 (41, 95)	Low
	Millet	35	28	81 (47, 133)	Mid-low
	Sorghum	35	36	112 (61, 195)	Mid-low
	Maize	36	43	72 (40, 121)	Low
Senegal	Wheat flour	21	66	35 (15, 57)	Low
	Rice	17	64	192 (141, 261)	Mid-high
	Millet	17	28	103 (48, 169)	Mid-low
	Sorghum	17	3	91 (57, 185)	Mid-low
	Maize	21	18	67 (30, 124)	Low
Togo	Wheat flour	53	17	8 (5, 15)	Low
	Rice	46	68	59 (34, 100)	Low
	Millet	49	1	55 (20, 134)	Low
	Sorghum	49	2	108 (46, 262)	Mid-low
	Maize	53	92	165 (83, 269)	Mid-high

Footnote. Beneficiary population is defined as the proportion of households with vehicle specific mean probability of inadequacy (MPI) > 0.75. Vehicle specific MPI was estimated by combining the simple average of the probability of inadequacy of the micronutrients that each vehicle could plausibly deliver based on global normative and operational guidance and feasibility studies (REF WHO 2016, 2018, 2022, 2025; WFP 2025; Tripathi et al. 2011, 2012). For wheat flour and edible oil, reach and per capita daily consumption reflect current programme reach of the fortifiable (market-acquired industrially

processed) form. For rice, maize, millet, and sorghum, they reflect total current consumption regardless of form, serving as a demand signal for prospective fortification. All presented results were estimated using the Enquête Harmonisée sur les Conditions de Vie des Ménages (EHCVM, 2021/2022), the Ghana Living Standards Survey (2016/2017) and Nigeria Living Standards Survey (2018/2019).
Coverage is defined as the percent of the beneficiary population that consumes the vehicle.
Median (IQR) per capita daily consumption (PCDC), in grams/adult female equivalent/day, was estimated among the beneficiary population for countries where vehicle coverage exceeded 10%.

Median consumption quantities were used to classify countries as *low* (<75g), *mid-low* (75g-149g), *mid-high* (150g-300g) and *high* (>300g) consumer categories, based on global normative and operational guidance (REF WHO 2016, 2018, 2022; WFP 2025)

Table S6. *Sensitivity analysis of the public health potential of wheat flour, rice and maize assuming addition of vitamin A, based on the beneficiary population size and characteristics of vehicle consumption amongst potential beneficiaries.*

Country	Vehicle	Beneficiary population[1] national, %	Of the beneficiary[1] population		
			Coverage[2], %	Median (IQR) PCDC	Consumption category[3]
Benin	Wheat flour	67	63	14 (6, 24)	Low
	Rice	61	85	76 (50, 112)	Mid-low
	Maize	67	90	161 (104, 242)	Mid-high
Burkina Faso	Wheat flour	71	40	11 (5, 21)	Low
	Rice	69	59	85 (52, 130)	Mid-low
	Maize	71	63	148 (75, 261)	Mid-low
Cote D'Ivoire	Wheat flour	77	69	16 (10, 25)	Low
	Rice	74	79	166 (111, 233)	Mid-high
	Maize	77	31	44 (25, 75)	Low
Ghana	Wheat flour	57	17	5 (1, 17)	Low
	Rice	49	65	76 (39, 140)	Mid-low
	Maize	57	45	68 (40, 114)	Low
Guinea-Bissau	Wheat flour	79	72	17 (9, 31)	Low
	Rice	67	98	276 (210, 364)	Mid-high
	Maize	79	4	55 (34, 94)	Low
Mali	Wheat flour	83	64	17 (9, 29)	Low
	Rice	81	89	150 (77, 236)	Mid-high
	Maize	83	33	133 (73, 224)	Mid-low
Niger	Wheat flour	79	23	10 (4, 21)	Low
	Rice	87	76	136 (78, 222)	Mid-low
	Maize	79	41	194 (113, 297)	Mid-high
Nigeria	Wheat flour	66	73	28 (17, 48)	Low
	Rice	64	86	67 (44, 102)	Low
	Maize	66	40	73 (40, 127)	Low
Senegal	Wheat flour	74	82	45 (26, 63)	Low
	Rice	65	89	208 (160, 271)	Mid-high
	Maize	74	18	53 (23, 108)	Low
Togo	Wheat flour	78	19	9 (5, 16)	Low
	Rice	70	70	63 (37, 104)	Low
	Maize	78	92	165 (83, 272)	Mid-high

Footnote. Beneficiary population is defined as the proportion of households with vehicle specific mean probability of inadequacy (MPI) > 0.5. Vehicle specific MPI was estimated by combining the simple average of the probability of inadequacy of the micronutrients that each vehicle could plausibly deliver based on global normative and operational guidance and feasibility studies (REF WHO 2016, 2018, 2022, 2025; WFP 2025; Tripathi et al. 2011, 2012). For the sensitivity analysis vitamin A was added to the vehicle-specific MPI for wheat flour, rice, and maize to represent a scenario assuming full adherence to WHO fortification guidance, disregarding operational constraints associated with vitamin A fortification of cereal vehicles. For wheat flour and edible oil, reach and per capita daily consumption reflect current programme reach of the fortifiable (market-acquired industrially processed) form. For rice, maize, millet, and sorghum, they reflect total current consumption regardless of form, serving as a demand signal for prospective fortification. All presented results were estimated using the Enquête Harmonisée sur les Conditions de Vie des Ménages (EHCVM, 2021/2022), the Ghana Living Standards Survey (2016/2017) and Nigeria Living Standards Survey (2018/2019).
Coverage is defined as the percent of the beneficiary population that consumes the vehicle.
Median (IQR) per capita daily consumption (PCDC), in grams/adult female equivalent/day, was estimated among the beneficiary population for countries where vehicle coverage exceeded 10%.

Median consumption quantities were used to classify countries as *low* (<75g), *mid-low* (75g-149g), *mid-high* (150g-300g) and *high* (>300g) consumer categories, based on global normative and operational guidance (REF WHO 2016, 2018, 2022; WFP 2025)

Table S7. Sensitivity analysis of vulnerability to inadequate iron and zinc intake assuming bioavailability of 15% for iron (REF WHO 2006) and a refined diet for zinc (REF Allen et al., 2020)

		Percent vulnerable to inadequate intake			
Country	Socioeconomic position (by quintile)	Iron assuming 5% bioavailability (%)	Iron assuming 15% bioavailability (%)	Zinc assuming an unrefined diet (%)	Zinc assuming a refined diet (%)
Benin	National	57	35	77	46
	1 (poorest)	63	42	82	56
	2	60	37	82	50
	3	59	35	81	50
	4	56	32	76	42
	5 (wealthiest)	51	28	71	36
Burkina Faso	National	56	36	76	48
	1 (poorest)	66	47	85	66
	2	63	42	83	54
	3	57	36	77	46
	4	51	30	69	38
	5 (wealthiest)	45	25	64	31
Cote D’Ivoire	National	65	44	79	45
	1 (poorest)	74	54	89	62
	2	67	46	82	53
	3	63	42	81	46
	4	62	39	77	41
	5 (wealthiest)	61	38	74	34
Ghana	National	62	47	64	45
	1 (poorest)	77	66	81	64
	2	65	51	70	50
	3	61	44	63	40
	4	52	34	55	30
	5 (wealthiest)	43	25	44	19
Guinea-Bissau	National	74	53	81	56
	1 (poorest)	72	53	82	62
	2	74	54	82	57
	3	72	50	80	52
	4	77	56	82	57
	5 (wealthiest)	76	54	80	52
Mali	National	73	49	81	49
	1 (poorest)	80	60	92	69
	2	76	54	88	57
	3	73	49	83	49
	4	70	44	79	44
	5 (wealthiest)	68	44	77	41
Niger	National	58	34	81	43
	1 (poorest)	54	32	82	44
	2	52	29	81	39
	3	53	31	78	38
	4	57	33	79	32
	5 (wealthiest)	60	35	76	35
Nigeria	National	55	31	75	43
	1 (poorest)	79	55	93	76
	2	62	34	85	54
	3	53	27	76	42
	4	45	21	70	32
	5 (wealthiest)	38	19	58	26
Senegal	National	72	47	81	48
	1 (poorest)	81	60	89	64
	2	73	49	83	50
	3	71	45	82	47
	4	69	42	78	40
	5 (wealthiest)	60	32	66	26
Togo	National	69	48	85	61
	1 (poorest)	72	51	87	66
	2	72	51	89	66
	3	71	50	88	68
	4	68	47	87	65
	5 (wealthiest)	65	43	86	60

Table S8. Summary of national micronutrient survey results from Ghana, Nigeria, and Senegal reporting the prevalence of deficiencies in vitamin A and zinc among children aged 12–59 months and folate, iron, and anaemia among women of reproductive age, along with designations used in global guidance to determine when deficiency prevalence constitutes a public health problem requiring population intervention.

Micronutrient	Target group	Country	Prevalence of deficiency	Public Health Problem (PHP)[1]
Vitamin A	12-59m children	Ghana[2]	20.8	Severe PHP
		Nigeria[3]	31.3	Severe PHP
		Senegal[4]	12.1	Moderate PHP
Zinc	12-59m children	Ghana[2]	-	N/A
		Nigeria[3]	35.2	PHP
		Senegal[4]	-	N/A
Anaemia	15-49y women	Ghana[2]	21.7	Moderate PHP
		Nigeria[3]	23.3	Moderate PHP
		Senegal[4]	28.9	Moderate PHP
Iron	15-49y women	Ghana[2]	13.7	Mild PHP
		Nigeria[3]	10.0	Mild PHP
		Senegal[4]	42.3	Severe PHP
Folate	15-49y women	Ghana[2]	53.8	N/A
		Nigeria[3]	91.2	N/A
		Senegal[4]	50.2	N/A
Vitamin B12	15-49y women	Ghana[2]	6.9	N/A
		Nigeria[3]	1.6	N/A
		Senegal[4]	-	N/A

[1] Classification of a Public Health Problem were consistent with the definitions presented by the World Health Organization for vitamin A, iron, and anaemia and the International Zinc Nutrition Consultative Group (IZiNCG) for zinc.

[2] Ghana Micronutrient Survey 2017 GroundWork LLC, KEMRI Wellcome Trust Research Programme (KWTRP), University of Ghana, University of Wisconsin–Madison. Ghana Micronutrient Survey 2017. Accra, Ghana; 2017

[3] Nigeria National Food Consumption and Micronutrient Survey 2021 Federal Government of Nigeria & International Institute of Tropical Agriculture. National Food Consumption and Micronutrient Survey 2021: Final Report. Ibadan, Nigeria: IITA; 2024.

[4] Senegal National Nutrition Survey 2018 Directorate of Food and Nutrition (DAN), FAO, Food and Human Nutrition Research Laboratory of Cheikh Anta Diop University (LARNAH), Institute of Food Technology (ITA), Ministry of Health and Social Action, National Agency of Statistics and Demography, National Nutrition Development Council, Pasteur Institute of Senegal, UNICEF. Senegal National Nutrition Survey 2018. Dakar, Senegal; 2018.

Table S9. Summary of per capita daily consumption estimates of staple foods from this study using Household Consumption & Expenditure surveys (HCES) compared to per capita daily availability (PCDA) estimates using the Food and Agriculture Organization Supply Utilisation Accounts (SUA).

Staple food	Year	Country	Per capita daily availability from SUA (g/d per capita)	Per capita daily consumption from HCES (g/d per AFE[1])
Wheat	2022	Burkina Faso	36	8
	2022	Côte D'Ivoire	63	10
	2017	Ghana	56	7
	2022	Guinea-Bissau	49	8
	2022	Niger	16	8
	2018	Nigeria	69	19
	2022	Senegal	120	34
Edible oil[2]	2022	Burkina Faso	9	11
	2022	Côte D'Ivoire	18	10
	2017	Ghana	21	16
	2022	Guinea-Bissau	48	25
	2022	Niger	13	16
	2018	Nigeria	67	12
	2022	Senegal	39	47
Rice	2022	Burkina Faso	136	95
	2022	Côte D'Ivoire	330	171
	2017	Ghana	138	89
	2022	Guinea-Bissau	576	277
	2022	Niger	124	140
	2018	Nigeria	97	72
	2022	Senegal	334	213
Maize	2022	Burkina Faso	162	198
	2022	Côte D'Ivoire	65	62
	2017	Ghana	61	257
	2022	Guinea-Bissau	24	69
	2022	Niger	4	199
	2018	Nigeria	91	87
	2022	Senegal	66	88
Millet	2022	Burkina Faso	89	175
	2022	Côte D'Ivoire	4	38
	2017	Ghana	11	42
	2022	Guinea-Bissau	29	77
	2022	Niger	240	320
	2018	Nigeria	23	90
	2022	Senegal	67	77
Sorghum	2022	Burkina Faso	163	231
	2022	Côte D'Ivoire	6	36
	2017	Ghana	14	83
	2022	Guinea-Bissau	27	99
	2022	Niger	156	258
	2018	Nigeria	74	118
	2022	Senegal	31	84

[1] Adult female equivalent

[2] In the SUA estimate of per capita daily availability, includes soyabean oil, groundnut oil, sunflower seed oil, rape and mustard seed oil, cottonseed oil, and palm oil

Table S10. Included countries and the corresponding Household Consumption and Expenditure Survey (HCES) used in this study

Country	HCES Survey (Year)	Survey Type	Enumeration areas	Households, n	Food items, n	Recall period	Food data type	Time horizon
Benin	Enquête Harmonisée sur le Conditions de Vie des Ménages, (2021-2022)	Cluster panel	670	8032	176	Previous 7 days	Household consumption quantities	Nov 2021 - July 2022
Burkina Faso	Enquête Harmonisée sur le Conditions de Vie des Ménages, (2021-2022)	Cross sectional	585	7176	176	Previous 7 days	Household consumption quantities	Sept 2021 - June 2022
Cote D'Ivoire	Enquête Harmonisée sur le Conditions de Vie des Ménages, (2021-2022)	Cluster panel	1084	13,693	176	Previous 7 days	Household consumption quantities	Nov 2021 - July 2022
Guinea-Bissau	Enquête Harmonisée sur le Conditions de Vie des Ménages, (2021-2022)	Cluster panel	450	5351	177	Previous 7 days	Household consumption quantities	Nov 2021 - July 2022
Ghana	Ghana Living Standards Survey (2016-2017)	Cross sectional	1000	14,009	188	Diary to record daily food expenditure for 18 days	Household food expenditure	Oct 2016 - Oct 2017
Mali	Enquête Harmonisée sur le Conditions de Vie des Ménages, (2021-2022)	Cluster panel	500	6143	176	Previous 7 days	Household consumption quantities	Oct 2022 - July 2022
Niger	Enquête Harmonisée sur le Conditions de Vie des Ménages, (2021-2022)	Cluster panel	504	6622	176	Previous 7 days	Household consumption quantities	Nov 2021 - Aug 2022
Nigeria	Living Standards Survey, (2018-2019)	Cross sectional	2213	22,118	148	Previous 7 days	Household consumption quantities	Sept 2018 – Sept 2019
Senegal	Enquête Harmonisée sur le Conditions de Vie des Ménages, (2021-2022)	Cluster panel	598	7120	176	Previous 7 days	Household consumption quantities	Dec 2021 - Aug 2022
Togo	Enquête Harmonisée sur le Conditions de Vie des Ménages, (2021-2022)	Cluster panel	540	6462	176	Previous 7 days	Household consumption quantities	Oct 2021 - July 2022

Table S11. Food items in each Household Consumption and Expenditure Survey classified as or containing fortifiable staple grains and industrially produced edible oils.

Staple food item	Survey	HCES food item included	Proportion of food item containing staple food[1]
Wheat flour	EHCVM	Wheat	1
		Local or imported wheat flour	1
		Modern bread	0.75
		Traditional bread	0.75
		Biscuits	0.63
		Cakes	0.64
		Fritters, pancakes	0.64
	NGA	Wheat flour	1
		Bread	0.74
		Cake	0.64
		Buns/Pofpof/Donuts	0.64
		Biscuits	0.63
		Meat pie/Sausage roll	0.28
	GHA	Wheat flour	1
		Bread, wheat, wholemeal, unfortified	0.75
		Bread/rolls, white, unfortified	0.75
		Cookies and bars, brand name	0.73
		Cookies and bars, unknown type	0.73
Edible oil	EHCVM	Refined peanut oil	1
		Soy oil	1
		Cottonseed oil	1
		Refined Palm oil	1
		Other oils	1
	NGA	Coconut oil	1
		Other oil and fat	1
		Groundnut oil	1
	GHA	Coconut oil	1
		Peanut oil	1
		Vegetable oil	1
Rice	EHCVM	Broken local rice	1
		Whole local rice	1
		Broken imported rice	1
		Whole imported rice	1
	NGA	Local rice	1
		Imported rice	1
	GHA	Local rice	1
		Imported rice	1
Sorghum	EHCVM	Sorghum	1
	NGA	Guinea corn/Sorghum	1
	GHA	Sorghum	1
Millet	EHCVM	Millet	1
		Millet flour	1

	NGA	Millet	1
	GHA	Millet	1
		Millet flour	1
Maize	EHCVM	Corn flour	1
		Corn grain/kernel	1
		Corn semolina	1
	NGA	Maize flour	1
		Maize (shelled/off the cob)	1
	GHA	Maize, dry, raw (corn dough)	1
		Maize (white)	1
		Maize (yellow)	1

[1] Proportion of wheat flour as part of wheat flour products were derived by the UCDavis MINIMOD project using local recipe data from Nigeria, Ghana, and Cameroon.

Table S12. Fortification recommendations for wheat flour, edible oil, rice, sorghum, millet, and maize presented in global normative guidance and experimental research

Vehicle	Micronutrients	Source	Notes
Wheat flour	Vitamin B12, folic acid, iron, zinc	WHO Guidelines: wheat flour	Consider adding VA when deficiency constitutes a PHP and no other strategies to address it are in place.
Edible oil	Vitamin A	WHO Guidelines: Edible oils and fats	
Rice	Iron, folic acid, vitamin B12	WHO Guidelines: Rice fortification; WFP specification for fortified rice	
Sorghum	Iron, zinc, folic acid	Tripathi and Platel 2011 and Tripathi et al. 2012	
Millet	Iron, zinc, folic acid	Tripathi and Platel 2011 and Tripathi et al. 2012	
Maize	Vitamin B12, folic acid, iron, zinc	WHO Guidelines Maize flour/corn meal	Other B-vitamins are specified for restitution of content loss during milling.
