# Supplementary material for: Diversified food fortification portfolios can enhance coverage of micronutrient-vulnerable populations in West Africa
Source: Nat Food. 2026 Sep 2;7(9):902–16. doi: 10.1038/s43016-026-01412-2 (PMC13593565; doi:10.1038/s43016-026-01412-2)
Supplement: Supplementary file 2 [file 43016_2026_1412_MOESM2_ESM.pdf]

nature portfolio

Corresponding author(s): Kevin Tang

Last updated by author(s): Jul 20, 2026

# Reporting Summary

Nature Portfolio wishes to improve the reproducibility of the work that we publish. This form provides structure for consistency and transparency in reporting. For further information on Nature Portfolio policies, see our Editorial Policies and the Editorial Policy Checklist.

## Statistics

For all statistical analyses, confirm that the following items are present in the figure legend, table legend, main text, or Methods section.

| n/a | Confirmed | |
|---|---|---|
| ☐ | ☒ | The exact sample size ($n$) for each experimental group/condition, given as a discrete number and unit of measurement |
| ☐ | ☒ | A statement on whether measurements were taken from distinct samples or whether the same sample was measured repeatedly |
| ☒ | ☐ | The statistical test(s) used AND whether they are one- or two-sided<br>*Only common tests should be described solely by name; describe more complex techniques in the Methods section.* |
| ☐ | ☒ | A description of all covariates tested |
| ☐ | ☒ | A description of any assumptions or corrections, such as tests of normality and adjustment for multiple comparisons |
| ☐ | ☒ | A full description of the statistical parameters including central tendency (e.g. means) or other basic estimates (e.g. regression coefficient) AND variation (e.g. standard deviation) or associated estimates of uncertainty (e.g. confidence intervals) |
| ☒ | ☐ | For null hypothesis testing, the test statistic (e.g. $F$, $t$, $r$) with confidence intervals, effect sizes, degrees of freedom and $P$ value noted<br>*Give P values as exact values whenever suitable.* |
| ☒ | ☐ | For Bayesian analysis, information on the choice of priors and Markov chain Monte Carlo settings |
| ☒ | ☐ | For hierarchical and complex designs, identification of the appropriate level for tests and full reporting of outcomes |
| ☒ | ☐ | Estimates of effect sizes (e.g. Cohen's $d$, Pearson's $r$), indicating how they were calculated |

*Our web collection on statistics for biologists contains articles on many of the points above.*

## Software and code

Policy information about availability of computer code

| | |
|---|---|
| Data collection | No software was used by the research team for data collection. This study is a secondary analysis of existing survey microdata. Data were collected by the national statistical agencies of each country where details of the instruments and collection software are documented in the respective survey reports. |
| Data analysis | All analyses were conducted in R (version 4.5.1). Analysis code is publicly available at https://github.com/MIMI-wfp/West-Africa-LSFF-Analysis. |

For manuscripts utilizing custom algorithms or software that are central to the research but not yet described in published literature, software must be made available to editors and reviewers. We strongly encourage code deposition in a community repository (e.g. GitHub). See the Nature Portfolio guidelines for submitting code & software for further information.

## Data

Policy information about availability of data

All manuscripts must include a data availability statement. This statement should provide the following information, where applicable:

- Accession codes, unique identifiers, or web links for publicly available datasets
- A description of any restrictions on data availability
- For clinical datasets or third party data, please ensure that the statement adheres to our policy

The household survey data underlying the vulnerability to inadequate intake estimates are held by third parties and are available from their original sources. The EHCVM (Enquête Harmonisée sur les Conditions de Vie des Ménages) and Nigeria Living Standards Survey data are available through the World Bank Microdata

Library (https://microdata.worldbank.org/index.php/catalog/lsms), subject to user registration and acceptance of the Microdata Library's terms of use, which do not permit redistribution of the data by third parties. The Ghana Living Standards Survey data are available from the Ghana Statistical Service under equivalent conditions (https://microdata.statsghana.gov.gh/index.php/catalog/97). The authors received no special access privileges to these data. All additional data sources used in this analysis, together with details of how each was accessed, are documented in the README of the project repository (https://github.com/MIMI-wfp/West-Africa-LSFF-Analysis).

## Research involving human participants, their data, or biological material

Policy information about studies with human participants or human data. See also policy information about sex, gender (identity/presentation), and sexual orientation and race, ethnicity and racism.

| | |
|---|---|
| Reporting on sex and gender | No applicable as we are using household level data. |
| Reporting on race, ethnicity, or other socially relevant groupings | Not applicable as we did not report according to race, ethnicity or other socially relevant groupings. |
| Population characteristics | See above |
| Recruitment | Participants were not recruited by the research team. Households were selected by the responsible national statistical agencies through multi-stage stratified probability sampling from national sampling frames. Non-response was addressed using sampling weights supplied by the agencies. |
| Ethics oversight | This study involved secondary analysis of fully de-identified, publicly available household survey microdata. It did not involve any interaction with human participants or access to identifiable information, and ethical approval was therefore not required. The original surveys were conducted by the national statistical agencies of each country under their respective national statistical legislation and governance frameworks, which include obtaining informed consent from participating households at the time of data collection. Access to the microdata was granted subject to the data providers' terms of use. |

Note that full information on the approval of the study protocol must also be provided in the manuscript.

# Field-specific reporting

Please select the one below that is the best fit for your research. If you are not sure, read the appropriate sections before making your selection.

☐ Life sciences ☒ Behavioural & social sciences ☐ Ecological, evolutionary & environmental sciences

For a reference copy of the document with all sections, see nature.com/documents/nr-reporting-summary-flat.pdf

# Behavioural & social sciences study design

All studies must disclose on these points even when the disclosure is negative.

| | |
|---|---|
| Study description | Secondary quantitative analysis of nationally representative Household Consumption and Expenditure Survey (HCES) microdata from ten West African countries. The study is cross-sectional and observational, using household food consumption data to estimate apparent nutrient supply, vulnerability to inadequate micronutrient intake, and consumption of staple foods considered as food fortification vehicles. |
| Research sample | The sample comprises all households surveyed in nationally representative HCES from ten West African countries: Benin, Burkina Faso, Côte d'Ivoire, Guinea-Bissau, Mali, Niger, Senegal and Togo (Enquête Harmonisée sur les Conditions de Vie des Ménages, EHCVM, 2021–2022); Ghana (Ghana Living Standards Survey, 2016–2017); and Nigeria (Nigeria Living Standards Survey, 2018–2019). Country-specific sample sizes are reported in Supplementary Table 10. Each survey was designed by the responsible national statistical agency to be representative at national, urban/rural and first administrative (ADM1) level. Household rosters record the age and sex of all members, which were used to derive adult female equivalents; the surveys cover the general population of all ages and both sexes. Countries were selected to include as many ECOWAS and/or Alliance des États du Sahel member states as possible for which a recent (within 10 years) nationally representative HCES was available, supporting regionally comparable estimates. |
| Sampling strategy | No sample size calculation was performed by the authors. This study uses existing survey data; all households with complete food consumption data were included, so sample sizes are determined by the original survey designs and are reported in Supplementary Table 10. Each survey used multi-stage stratified probability sampling implemented by the responsible national statistical agency, with stratification by region and urban/rural residence, selection of enumeration areas, and selection of households within them. These sample sizes are sufficient because each survey was powered by its originating agency to produce nationally and subnationally (ADM1) representative estimates, which is the level at which all results are reported. |
| Data collection | Data were collected by the national statistical agencies of each country, not by the research team. Food consumption was recorded at household level using a standardised fixed food item list with a multi-day recall period (typically seven days), alongside food acquisition sources and prices, household demographics, expenditure, assets, education and health indicators. The authors obtained de-identified microdata from the World Bank Microdata Library and the Ghana Statistical Service. No data were collected directly from participants by the research team, and no researcher was present during data collection. |

| | |
|---|---|
| Timing | Data collection was conducted by the responsible national statistical agencies. The EHCVM surveys in Benin, Burkina Faso, Côte d'Ivoire, Guinea-Bissau, Mali, Niger, Senegal and Togo were conducted in 2021–2022; the Ghana Living Standards Survey in 2016–2017; and the Nigeria Living Standards Survey in 2018–2019. Survey-specific collection periods are summarised in Supplementary Table 10. |
| Data exclusions | No households were excluded on the basis of their characteristics. Outlying food quantity values, likely reflecting recall error, were identified separately for each country and food item among consuming households: quantities were log-transformed and values exceeding the consumer mean plus four standard deviations on the log scale were flagged and replaced with the geometric mean of consumer-reported quantities for that item in that country. This procedure was part of the standardised data preparation pipeline applied uniformly across all ten countries. |
| Non-participation | Non-response and refusal were handled by the national statistical agencies during fieldwork and are documented in the respective survey reports. Sampling weights provided by the agencies were applied throughout to account for survey design and non-response. No participants dropped out of the present study, which is a secondary analysis of completed surveys. |
| Randomization | Randomization is not relevant to this study. This is an observational, cross-sectional secondary analysis with no experimental groups and no intervention allocation. Results are stratified by pre-existing characteristics — country, first administrative region, and wealth quintile — which are descriptive rather than assigned. Sampling weights were applied so that estimates are representative of the underlying populations. |

# Reporting for specific materials, systems and methods

We require information from authors about some types of materials, experimental systems and methods used in many studies. Here, indicate whether each material, system or method listed is relevant to your study. If you are not sure if a list item applies to your research, read the appropriate section before selecting a response.

## Materials & experimental systems

| n/a | Involved in the study |
|---|---|
| ☒ | ☐ Antibodies |
| ☒ | ☐ Eukaryotic cell lines |
| ☒ | ☐ Palaeontology and archaeology |
| ☒ | ☐ Animals and other organisms |
| ☒ | ☐ Clinical data |
| ☒ | ☐ Dual use research of concern |
| ☒ | ☐ Plants |

## Methods

| n/a | Involved in the study |
|---|---|
| ☒ | ☐ ChIP-seq |
| ☒ | ☐ Flow cytometry |
| ☒ | ☐ MRI-based neuroimaging |

## Plants

| | |
|---|---|
| Seed stocks | N/A |
| Novel plant genotypes | N/A |
| Authentication | N/A |
