# Supplementary material for: Diversified food fortification portfolios can enhance coverage of micronutrient-vulnerable populations in West Africa
Source: Nat Food. 2026 Sep 2;7(9):902–16. doi: 10.1038/s43016-026-01412-2 (PMC13593565; doi:10.1038/s43016-026-01412-2)
Supplement: Supplementary file 3 [file 43016_2026_1412_MOESM3_ESM.pdf]

nature portfolio

Peer Review File

Diversified food fortification portfolios can enhance coverage to micronutrient vulnerable populations in West Africa

Corresponding Author: Dr Kevin Tang

Version 1:

Reviewer comments:

Reviewer #1

(Remarks to the Author)
Title: Potential of fortifiable staple foods for addressing micronutrient vulnerability in West Africa (Manuscript NATFOOD-26020600A)
Summary: This paper sought to link nutritional vulnerability with access to fortifiable staples, with the goal of providing a framework to policymakers. In the revised version, authors connect these well and provide a useful table for selecting a crop/region for promoting fortification and consumption.
Reviewer's Comments (upload to journal portal):
1. While rice had the highest reach, some commentary on possible detrimental effects of promoting rice consumption may be warranted – regarding effects on glucose, etc., particularly if the "nutrition transition" or "westernization" is beginning to occur in Western Africa/Africa in general.
2. Table 3: If I understand the analysis correctly, it may be useful to have a traffic light or other ranking system to show which vehicle-coverage-consumption combination has the most potential or largest gain. For example, for Benin, it seems maize has the highest potential – with high coverage paired with mid-high intake.

(Remarks on code availability)

Reviewer #2

(Remarks to the Author)
This is a well-written paper that uses widely available secondary data to estimate population-level vulnerabilities to vitamin A, folate, vitamin B12, iron, and zinc inadequacy alongside estimates of reach and consumption of existing and emerging food vehicles for fortification. The paper covers an impressive number of countries (including subnational estimates for each country) and presents a framework that could potentially be applied in other contexts.

My main concern is that, throughout the paper, the authors refer to estimates of the reach/coverage and consumption of fortifiable staples, when in fact they have not accounted for whether or not the specific form of the food vehicle being consumed is fortifiable. For example, they categorize all maize, sorghum, and millet consumption as fortifiable, when in fact only maize, sorghum, and millet consumed in the form of flour that was industrially processed is considered fortifiable (there are examples of flour fortification at small-scale mills, but not any at scale in West Africa that I am aware of). This is a known shortcoming of using HCES data for fortification modeling. It is very difficult to accurately determine consumption of fortifiable vs non-fortifiable foods, but in this case, the authors do not even make an attempt. Rather, they include home-produced staples in their estimates of fortifiable staple consumption, whole grain forms of maize, sorghum, and millet, and do not consider the extent of industrial processing of any of the vehicles in each country. This could result in very misleading "alignment" between inadequate intake and high levels of consumption/coverage of food vehicles, the conclusion that certain vehicles are equitable across socioeconomic position and/or geography when in fact they are not, etc. They do do a nice job discussing the issue of feasibility for fortification in the discussion section, but I think the language throughout the paper (references to fortifiable staples, etc) needs to be cleaned up for accuracy, and the authors need to revise their estimates of reach, coverage, and consumption to, at a minimum, exclude home-produced staples and whole grains.

Other specific comments are listed below.

Lines 27-28: Flagging for tense inconsistency in this sentence (analysed and then assess).

Lines 47-50: The way this sentence is written implies that all five of these micronutrients impact immune and cognitive function, pregnancy outcomes, and productivity, which is not the case (e.g., no well-established links between vitamin A and productivity or pregnancy outcomes). Consider revising to accurately describe these relationships instead of lumping them all together.

Lines 78-81: What is the current design, and what about it lacks adaptability, specifically? Spelling this out will help readers understand the relationship between regional standards/coordination and national fortification action.

Lines 81-85: Saying that regional coordination needs to promote common standards and also support countries to adapt seems a bit contradictory. Are you saying harmonized/common regional standards are necessary or that countries should set their own standards based on dietary patterns? This is the challenge with regional standards, right? Those standards might not be suited to meet the needs of individual countries, and if individual countries adapt them to country-specific standards, then the benefits of regional harmonization (e.g., ease of trade and monitoring/regulation) are no longer fully achievable.

Line 107: Consider specifying that access to a fortifiable form of the vehicle must be widespread, not just access to the vehicle itself (e.g., locally milled flour vs industrially milled flour).

Lines 108-109: Unclear what is meant by complementary vs alternative vehicles. What's the difference and when would you need one vs the other?

Line 127-129: An issue I see here is that HCES data do not lend themselves to identifying consumption of or access to fortifiable food vehicles (key being the fortifiable component). A household consumed, e.g., rice that they purchased, but you do not know if that rice was purchased from a large-scale rice mill (fortifiable) or from a small-scale local rice mill (likely not feasible for fortification).

Line 137: Typo (study').

Line 183: Just regional standards? What about national standards (the two do not fully align for all of these countries, right?).

Line 224: Above rather than less than 10%, right?

Lines 272-274: Here is an example of where I think this approach may generate misleading results. Do we really think the poorest populations in these countries are consuming industrially processed millet and/or sorghum flour? It seems doubtful.

Line 276-278: I'm having a hard time following the regional-national pathway here. Can you please explain how regional standards would help countries prioritize those most appropriate vehicles and fortification levels according to country-specific dietary patterns and vulnerability contexts? Where do the regional standard fit in, and what, specifically, about your results, suggest a role for regional standards (I'm not saying there is not a role, I just think you need to explain what you mean here, including how your results fit into what you are suggesting).

Line 291: What is the difference between complementary and alternative vehicles? Do you mean complementary interventions?

Line 293: Typo – missing the word "are" before updated.

Line 349: Here, you are citing the rice fortification landscape for Senegal that specifically states that "Mandatory fortification of all locally produced and imported rice is may be impractical at this stage in Senegal given the fragmentation of the milling industry where around 87 percent of rice is still milled through small-scale mills." This is not to say that rice may not be a good food vehicle in Senegal, but depending on consumption patterns among vulnerable populations (probably pretty reasonable to assume that they are more likely to consume rice from small-scale mills), it may not be equitable. For rice and other vehicles, your approach misses this important dimension of designing successful (and equitable) food fortification programs.

Lines 357-379: Ok, yes, this is exactly what I am referring to above. These types of considerations need to be more directly integrated into your analysis (for all food vehicles, not just rice) to the extent possible (at a minimum, exclude consumption of home-produced staples, do not include whole grain versions of staples).

Line 393: Please also comment on the likelihood that millet or sorghum are industrially milled at any significant scale in these countries (and hence whether millet or sorghum flour fortification would, in any way, be feasible). My assumption is that industrial milling is quite uncommon, but I'm happy to be proven wrong.

Lines 405-413: Here again, please explain what about your results suggest the need for regionally aligned standards. And are you suggesting here that the standards for all food vehicles be the same in the region (which micronutrients and fortification levels) but then countries choose which food vehicles to mandate? Again, I'm not quite following what the suggestions is here and this this section needs to be reworked a bit for clarity. Also, small point, but I'm not sure what, "market surveys require a regional reference standard" means.

Line 419: I think you should probably also acknowledge the fact that although the data that you are using does allow for

quantitative modeling of the potential impacts of fortification on micronutrient inadequacy, you have chosen not to do so here and that setting fortification standards (either establishing or adapting) should really be informed not only by which food vehicles are consumed in sufficient quantities by populations with micronutrient inadequacies, but also by directly modeling the impact of fortification of individual and combinations of food vehicles on the gap between intake and requirements, the prevalence of inadequacy, and the risk of high intakes.

Line 435: Missing word (I think it should read, "…we assessed…").

Lines 486-488: I may be wrong, but I think the Ghana HCES data are fairly substantially different from the other countries and likely required very different processing methods. If this is correct, please make that clear and describe the way the Ghana HCES were processed relative to the other surveys. I think it's important to point out that there can be wide variation in how these surveys are conducted country to country, requiring adapted methods for preparing the data for this type of analysis.

Line 492: Were conversion factors provided for all surveys? Ghana?

Lines 494-496: Please describe, specifically, how you identified and corrected outliers.

Line 503-505: Is there a well-established pathway from B12 deficiency to mortality? If yes, please provide a citation (for B12 as well as for the other micronutrients).

Lines 505-507: This line needs to be reviewed and corrected/nuanced for accuracy, as it currently reads as though each of these five micronutrients have the same consequences (impaired linear growth, cognitive development, and functional capacity). Please also add citations for each of the pathways you are claiming (by micronutrient and outcome).

Lines 532-533: Please add a few lines here with additional detail on how AFEs were calculated for each county. What was the basis for energy requirements? Were the same energy requirements used for each country? How were breastfeeding children and pregnant and lactating women dealt with?

Line 541-543: I'm not quite following this line, "The overall MPI was estimated by combining the simple average of the probability of inadequacy for all five micronutrients." How were they combined? Do you mean "taking" the simple average rather than combining the simple average?

Line 552 and line 559: Maize flour instead of maize here, correct?

Lines 557-559: Hmm, Nigeria has a mandatory national standard for the fortification of maize flour so would not qualify as emerging by this definition, right?

Line 603: Millet flour and sorghum flour, correct?

Lines 629-630: What are the "operational constraints associated with vitamin A fortification of cereal vehicles"? Just off the top of my head, I know that both Nigeria and Malawi mandate the fortification of wheat flour and maize flour with vitamin A.

Lines 566-587: The subtitle of this section is "Access to fortifiable staple foods indicators", but what you describe here is access to staple foods (even including home-produced staples!), definitely not fortifiable staple foods. As I'm sure you know, access to a staple does not equal access to a fortifiable staple. Maize flour is an obvious example of this in many countries (e.g., GFDx shows that just 10% of maize flour in Togo is fortifiable). In Senegal, most of the locally milled rice is at very small scale and is therefore not feasible for fortification (87% according to this 2019 landscape analysis: https://www.nutritionintl.org/wp-content/uploads/2019/10/WFP-Lanscape-Analysis-Rice-Fortification-in-Senegal.pdf). And I would be surprised if the situation was not similar for millet and sorghum for many of these countries. You claim repeatedly in the introduction (and the title) that your analysis is linking nutritional vulnerability with access to fortifiable staples. But what this analysis misses (which may lead to very misleading suggestions for appropriate food vehicles to meet the micronutrient needs of the most vulnerable), is that access to fortifiable staples is often the lowest among the poorest, most rural (and often also the most nutritionally vulnerable) populations.

Table 1. Here, too, I'm not sure what "combining" the simple averages means. And if it means taking the simple average, the value does not seem correct for Burkina (average would be around .67?).

Table 1: The prevalence of vitamin A inadequacy for Nigeria seems significantly higher than other estimates I've seen. I'm curious what you assumed about palm oil. This paper describes how this issue was dealt with in a similar analysis via input from a stakeholder group in Nigeria: https://cdn.nutrition.org/article/S2475-2991(24)02456-9/fulltext

Table 2. Title has a small typo (two commas after rice).

Table 3: Small point, but the inclusion of "beneficiary population size" in the title does not seem accurate. Percent covered/reached is very different than the size of the population (e.g., if the beneficiary population is 50% in Nigeria, that population size would of course be extremely different than the size of a 50% beneficiary population in Guinea-Bissau).

Table S7: This table provides great context. Please add the survey year for each country.

Table S10: I am not following how maize, millet, and sorghum are fortifiable (as distinct from maize flour, millet flour, and sorghum flour – these are separate food items on most of the food lists). Biofortifiable, yes, but household consumption of whole grain maize, sorghum, and millet is not fortifiable. Please clarify or correct this.

Figure 2. Where is all that dietary folate coming from in Niger? You don't actually need to answer this question in the paper – it's just a bit surprising.

(Remarks on code availability)
I was able to access the code but did not review it in detail.

Reviewer #3

(Remarks to the Author)
General comments:
- This is an interesting and useful paper that provides important evidence that was previously missing in the LSFF research literature. The analysis is rigorously specified and includes very good data visualizations that help to present the findings in a succinct way. This paper will prove useful for policy-focused researchers and practitioners. Most of the edits requested below are very small and aimed at improving the flow of the presentation.
Specific comments:
- In some places the writing is not easy to follow, mainly due to sentence length, the combination of multiple infinitives, and long subordinate clauses. Some targeted revision would improve the paper's readability significantly. For example (not an exhaustive list):
o Lines 60 – 53: this sentence would be more readable if the final clause (line 6) were a separate sentence.
o Lines 72 – 74: restructuring this sentence so that it has fewer subordinate clauses would be helpful (especially the final clause at line 74).
o Lines 81-85: similar issues as in the above, with too many infinitives ("to promote", "to adapt", "to strengthen") making the primary point hard to track.
o Lines 96-100: similar issues as in the above.
- Line 98: A very brief definition or introduction of the term "vehicle" would likely help readers who are not familiar with LSFF.
- Lines 168 – 178: It would be more precise and clear to say "Vulnerability to inadequacy is higher at lower socioeconomic positions", or even "Poor households are more vulnerable to inadequacy." Firstly, since this is a cross-sectional analysis that doesn't observe changing income status across a panel of households, and secondly, as the phrasing at 170-171 is ambiguous about the direction of change.
- Lines 179 – 220: A very brief definition of the term "reach" would be useful here. I understand that the journal format puts the Methods section later, but a shortened version of the full definition from lines 568 to 571 would improve clarity as this term is not self-evident in the way that "consumption" is. This is already done (and helpful) for the definition of "coverage" at line 223-224.
- Lines 527 – 533: Please provide some brief discussion of whether the AFE conversion approach accounts for potential reporting error in the dietary recalls performed for household surveys. Perhaps the AFE approach is sufficiently equivalent to energy adjustment, but readers familiar with nutrition assessment will want to know whether this is the case.
- Lines 549 – 541: This is a very reasonable approach. I wonder, though, if it would be possible or useful to allow the bioavailability assumption to vary across households depending on the composition of reported diets. Households with higher consumption of animal-source foods would be subject to a higher assumed bioavailability of iron and zinc. Variation in consumption of animal-source foods is likely to be large in several countries, which would further exaggerate the variation in MPI for iron and zinc.
- Lines 583 – 585: If reach and consumption are measured using all forms of the food consumed, then doesn't this result in an over-estimate of the potential consumption of the fortified food vehicle? That is, the portion of consumption that is home produced is almost certainly not fortifiable. You may have good reason to keep this way of measuring reach and consumption, rather than limiting it to only market acquired foods. This is mentioned at lines 422 -425. However, please provide a bit more explanation on why this is the best approach and what the consequences might be.
- Multiple places: the term "Socioeconomic position by quintile" is perhaps unnecessarily long when these are really "expenditure quintiles" (or even "income quintiles" give longstanding usage in the literature). You are free to disagree if you strongly prefer this term, but since the terms "poor" and "wealthy" are also used elsewhere in the text, I suggest streamlining here as well.
Stray typos/errors:
- Line 435: "In conclusion, assessed…" – appears to be missing "we" or another subject of the verb "assessed".
- Table 2 title: extra comma after rice

(Remarks on code availability)

Version 2:

Reviewer comments:

Reviewer #1

(Remarks to the Author)
Authors have sufficiently addressed my comments.

(Remarks on code availability)

Reviewer #2

(Remarks to the Author)
The authors have done a very nice job revising their paper in response to my feedback and in providing comprehensive responses to each of my comments. While I'm still a little uncomfortable with including home-produced and whole grain consumption in the candidate fortification vehicles calculations, the authors' reframeing (bifurcation of objective 2) and improved discussion around fortifiability/need for investments in milling capacity, food technology, etc to make the leap from existing demand to fortification, are a reasonable way to get around this issue for this specific analaysis. I think the paper is ready for publication. Nice work!

(Remarks on code availability)

Reviewer #3

(Remarks to the Author)
The authors have carefully responded to my comments as well as those of the other reviewers, making improvements that clarify the methods and interpretation of the paper. I am confident that the revised paper meets the requirements for publication in Nature Food.

(Remarks on code availability)
The code is not formatted in such a way that allows for immediate reproducibility, as the underlying household survey data are not directly linked (which is appropriate for avoiding data use violations), and as the file structure is not set up within the scripts. Depending on the journal's requirements, a somewhat more detailed README would be helpful for 1) making it clear where the household survey data should be downloaded, and 2) how someone interested in reproducing the results should set up their own directories to run all scripts. However, the code appears to be complete and readily usable.

Open Access This Peer Review File is licensed under a Creative Commons Attribution 4.0 International License, which permits use, sharing, adaptation, distribution and reproduction in any medium or format, as long as you give appropriate credit to the original author(s) and the source, provide a link to the Creative Commons license, and indicate if changes were made.
In cases where reviewers are anonymous, credit should be given to 'Anonymous Referee' and the source.
The images or other third party material in this Peer Review File are included in the article’s Creative Commons license, unless indicated otherwise in a credit line to the material. If material is not included in the article’s Creative Commons license and your intended use is not permitted by statutory regulation or exceeds the permitted use, you will need to obtain

permission directly from the copyright holder.
To view a copy of this license, visit https://creativecommons.org/licenses/by/4.0/

Potential of fortifiable staple foods for addressing micronutrient vulnerability in West Africa

- POINT-BY-POINT –

Comment: Thank you for submitting your manuscript entitled "Variation in vulnerability to inadequate micronutrient intake and access to fortifiable staple foods in West Africa". We have given the paper our careful consideration and find the topic highly relevant and appreciate the level of granular detail of the data presented. However, due to certain shortcomings we are concerned that sending the current manuscript out to review could lead to unnecessary delays and quite possibly an undesirable outcome of the review process.

> **Response:** We thank the editor for their valuable feedback and for taking the time to review our manuscript. We have carefully considered the comment and suggestions in the revised version of the manuscript. The point raised will be addressed point by point, as outlined below, to further strengthen the manuscript and improve the impact and longevity of the piece.

Comment: In particular, we believe the connections between the two main components of the analysis could be strengthened and developed in a way that is generalizable and reproducible. While the manuscript identifies which staple foods are most consumed in particular areas — and therefore the most promising vehicles for fortification — and maps where specific nutrient deficiencies exist at the time of measurement, it is not yet clear how one would use this information to decide which foods to fortify, nor if that decision-making process would adapt as the underlying data evolve over time. If a central contribution of this work is providing a framework for merging these two bodies of information to guide fortification policy in a way that is reproducible across contexts and time periods, that logic should be made more explicit.

At minimum, this could be addressed through an expanded discussion section. You might also consider adding a table or figure illustrating a possible decision-making framework, or a brief case example showing how some of the findings could be applied in practice. These are suggestions rather than requirements; we welcome your own thinking on how best to address this gap. The key is that the paper should serve not only as a snapshot in time and a demonstration of why detailed data matter, but also as a piece that offers a clear conceptual contribution for researchers and policymakers working in this space.

> **Response:** We thank the Editor for this insightful comment and agree that strengthening the connection between vulnerability to inadequate micronutrient intake and access to fortifiable staple foods, and making this connection generalizable and reproducible, is central to the contribution of this work.
>
> To address this, we have substantially revised the manuscript to explicitly develop and present a structured analytical framework that integrates these two components to inform fortification decision-making. Specifically, we introduced a third objective that assesses the public health potential of each vehicle by combining: (i) the proportion of the population vulnerable to inadequate intake of the micronutrients that each vehicle can deliver, (ii) the coverage of the vehicle among this population, and (iii) the quantity of the vehicle consumed among those reached. These components are now clearly defined in the Methods and operationalized in the Results (Table 3), providing a transparent and reproducible approach for evaluating and comparing fortification vehicles across countries. We have also expanded the Discussion to explicitly articulate the decision logic implied by this framework, namely that vehicles are most appropriate where high levels of vulnerability align with high coverage and sufficient consumption. The revised Discussion further clarifies how this approach can be applied across contexts and updated over time as dietary patterns and vulnerability profiles evolve, addressing the need for generalizability and adaptability highlighted in the comment. In addition, the Introduction and Abstract have been revised to foreground this framework as a central contribution of the manuscript, positioning the study not only as a cross-country analysis but as a practical and reproducible approach for linking nutritional vulnerability with access to fortifiable foods to guide policy.
>
> Together, these revisions strengthen the integration between the two components of the analysis and clarify how the findings can be used to

support systematic, data-driven prioritization of fortification strategies across settings and over time.

Potential of fortifiable staple foods for addressing micronutrient vulnerability in West Africa

-POINT-BY-POINT-

Table of Contents

Reviewer #1 ... ***2***

Major Comments ... **3**

Reviewer #2 ... ***4***

Major Comments ... **5**

Differentiated analysis of current vs. candidate fortification vehicles ... 5
Regional-national policy logic ... 10
Sorghum & millet fortification ... 14
Micronutrient-specific health pathways ... 17
Methodological assumption documentation ... 19
Expand description of surveys details ... 21
Analysis approach ... 22

Minor Comments ... **25**

Reviewer #3 ... ***28***

Major comments ... **29**

Differentiated analysis of current vs. candidate fortification vehicles ... 29
Definition of key terms ... 30
Methodological assumption documentation ... 32

Minor comments ... **34**

Reviewer #1

General perspectives

Comment: This paper sought to link nutritional vulnerability with access to fortifiable staples, with the goal of providing a framework to policymakers. In the revised version, authors connect these well and provide a useful table for selecting a crop/region for promoting fortification and consumption.

Response: We thank the reviewer for their thoughtful review of our work.

Major Comments

Comment: While rice had the highest reach, some commentary on possible detrimental effects of promoting rice consumption may be warranted – regarding effects on glucose, etc., particularly if the “nutrition transition” or “westernization” is beginning to occur in Western Africa/Africa in general.

> **Response:** We thank the reviewer for flagging this important concept. Our study's scope is the identification of fortifiable vehicles with the reach and production characteristics needed to address micronutrient adequacy gaps. It does not advocate increased rice consumption. Fortification adds nutrients to staples already consumed, it does not alter nor promote consumption levels. Concerns about rice intake are therefore separate questions from the ones we address. The broader questions the reviewer raises (i.e., glycaemic impact and the nutrition transition in West Africa) are important but lie outside our analytical scope, which does not model health outcomes or longitudinal shifts in consumption. Therefore, we have not added commentary on these themes.

Comment (Table 3): If I understand the analysis correctly, it may be useful to have a traffic light or other ranking system to show which vehicle-coverage-consumption combination has the most potential or largest gain. For example, for Benin, it seems maize has the highest potential – with high coverage paired with mid-high intake.

> **Response:** We thank the reviewer for this interesting suggestion. A single ranking or traffic-light system would collapse a multidimensional assessment (i.e., micronutrient-specific intake inadequacy, reach, consumption, fortifiable form coverage) into one score, and we think this would mislead rather than help. The reviewer's example illustrates the risk: in Benin, maize has high reach and substantial intake, but its potential as a fortification vehicle depends on the share of maize consumed in fortifiable form, which can sharply constrain feasibility. A ranking that omits this dimension would point to the wrong vehicle. What counts as the "greatest potential contribution" depends on which micronutrient is being addressed, where a vehicle strong for iron may be weak for vitamin A, and one ranking cannot capture both. We have kept Table 3 in its current form, which preserves the dimensions readers need to consider.

Reviewer #2

General perspectives

Comment: This is a well-written paper that uses widely available secondary data to estimate population-level vulnerabilities to vitamin A, folate, vitamin B12, iron, and zinc inadequacy alongside estimates of reach and consumption of existing and emerging food vehicles for fortification. The paper covers an impressive number of countries (including subnational estimates for each country) and presents a framework that could potentially be applied in other contexts.

My main concern is that, throughout the paper, the authors refer to estimates of the reach/coverage and consumption of fortifiable staples, when in fact they have not accounted for whether or not the specific form of the food vehicle being consumed is fortifiable. For example, they categorize all maize, sorghum, and millet consumption as fortifiable, when in fact only maize, sorghum, and millet consumed in the form of flour that was industrially processed is considered fortifiable (there are examples of flour fortification at small-scale mills, but not any at scale in West Africa that I am aware of). This is a known shortcoming of using HCES data for fortification modeling. It is very difficult to accurately determine consumption of fortifiable vs non-fortifiable foods, but in this case, the authors do not even make an attempt. Rather, they include home-produced staples in their estimates of fortifiable staple consumption, whole grain forms of maize, sorghum, and millet, and do not consider the extent of industrial processing of any of the vehicles in each country. This could result in very misleading “alignment” between inadequate intake and high levels of consumption/coverage of food vehicles, the conclusion that certain vehicles are equitable across socioeconomic position and/or geography when in fact they are not, etc. They do a nice job discussing the issue of feasibility for fortification in the discussion section, but I think the language throughout the paper (references to fortifiable staples, etc) needs to be cleaned up for accuracy, and the authors need to revise their estimates of reach, coverage, and consumption to, at a minimum, exclude home-produced staples and whole grains.

> **Response:** We thank the reviewer for the careful read and for identifying what we agree was the central methodological weakness of the original submission. The reviewer is correct that treating all reported staple consumption as equivalent to fortifiable consumption conflated current programme reach with future programme potential. We have restructured the analysis to address this directly, by differentiating how reach, coverage, and consumption are measured for current regional standard vehicles (wheat flour, edible oil) versus candidate vehicles not currently included in regional standards (rice, maize, millet, sorghum). The specific implementation, including the objectives and methods bifurcation, context and terminology precision, results restructure, and revised introduction and discussion, is described in our responses to the reviewer's individual comments below.

Major Comments

Differentiated analysis of current vs. candidate fortification vehicles

Preface: We thank the reviewer for their careful attention to our study's treatment of potential vehicles. As the reviewer notes, the original manuscript conflated consumption of a staple with the potential of the staple as a vehicle for fortification and we recognise that the appropriate measure depends on the maturity of each vehicles' fortification pathway. To address this, the measure of access is now determined by the vehicle's position in the regional fortification standards, and this principle is applied consistently across the analysis.

In the revisions, vehicles included in current ECOWAS regional standards (wheat flour, edible oil) are backed by industrial supply chains. For these vehicles, we now report the fortifiable share, operationalized as the market-purchased portion of consumption as a proxy for industrial processing. Candidate vehicles considered for inclusion (rice, maize, millet, sorghum) do not yet have an established fortification pathway. For these vehicles, we retain total consumption regardless of source. The fortification pathways for these vehicles are less developed and need to be informed by pre-existing demand for the staple, so total consumption reflects the full demand that would justify and shape future fortification infrastructure. This is the relevant measure for the policy question these candidate vehicles raise, with an emphasis on advocacy over operationalisation. Most substantially, the revisions now divide these two different analyses into two different study objectives to provide more overall clarity to the manuscript's narrative. These two objective are:

2. To assess fortification vehicles included in the current regional standards (wheat flour, edible oil) by examining their reach and consumption.
3. To assess other staple foods (rice, maize, millet, sorghum) as candidate fortification vehicles in the region by examining current consumption patterns to identify opportunities for future programme development.

The reviewers' individual comments are addressed in turn below within this framing.

Comment (Line 107): Consider specifying that access to a fortifiable form of the vehicle must be widespread, not just access to the vehicle itself (e.g., locally milled flour vs industrially milled flour).

> **Response:** We thank the reviewer for bringing attention to this intricacy and we agree that it is necessary to provide more attention to the fact that the staples already included in the regional guidance must be fortifiable forms of the vehicle to provide a public health contribution. Additionally, to provide clarity for the research question for the candidate vehicles as described in the preface, we have rephrased the argument so it now reads as follows: "For vehicles already included in regional standards, this requires quantifying whether the fortifiable forms of the vehicle reach vulnerable subgroups; for candidate vehicles outside standards, the prior question is whether fortification is feasible at all given prevailing consumption patterns."

Comment (Line 127-129): An issue I see here is that HCES data do not lend themselves to identifying consumption of or access to fortifiable food vehicles (key being the fortifiable component). A household consumed, e.g., rice that they purchased, but you do not know if that rice was purchased from a large-scale rice mill (fortifiable) or from a small-scale local rice mill (likely not feasible for fortification).

> **Response:** We thank the reviewer for flagging this point. We agree that HCES data cannot, on their own, distinguish whether a household consumed a fortifiable form of a vehicle (e.g., industrially milled rice) from a non-fortifiable form (e.g., rice from small-scale local milling). We have addressed this structurally in the revised manuscript rather than within Objective 4 itself.
>
> Specifically, we have bifurcated the original Objective 2 into two distinct objectives that make the analytical treatment of fortifiable form explicit. Objective 2 now examines vehicles already included in regional fortification standards (wheat flour, edible oil), for which we estimate coverage of the fortifiable form (as detailed in the revised Methods). Objective 3 examines candidate vehicles considered for inclusion (rice, sorghum, millet, maize), for which we estimate total consumption, which is appropriate because the policy question for these grains is whether demand exists to justify future infrastructure investment, not whether industrial fortified supply currently reaches consumers. With this distinction established upstream, Objective 4's reference to "the extent to which these potential beneficiaries could be covered" carries the appropriate interpretation for each vehicle: fortifiable-form coverage where industrial supply chains exist, and consumption-pattern alignment where they do not yet.

Comment (Line 349): Here, you are citing the rice fortification landscape for Senegal that specifically states that "Mandatory fortification of all locally produced and imported rice may be impractical at this stage in Senegal given the fragmentation of the milling industry where around 87 percent of rice is still milled through small-scale mills." This is not to say that rice may not be a good food vehicle in Senegal, but depending on consumption patterns among vulnerable populations (probably pretty reasonable to assume that they are more likely to consume rice from

small-scale mills), it may not be equitable. For rice and other vehicles, your approach misses this important dimension of designing successful (and equitable) food fortification programs.

> **Response:** We thank the reviewer for raising this important equity dimension. We agree that the original draft did not sufficiently address how decentralised milling interacts with the equitable reach of rice fortification, and have substantially restructured the rice discussion paragraph (new P7) to make this explicit.
> The revised paragraph (1) articulates the two pathways through which rice fortification operates in West Africa (fortification of imports at the border, and fortification of domestically produced rice through consolidating small- and medium-scale milling); (2) acknowledges the equity risk explicitly, stating that "populations that consume the most rice may have limited access to a fortified product, unless capacity for supply chains and milling infrastructure can be strengthened"; and (3) identifies the data gap that limits a more precise equity assessment, calling for research mapping intra-regional rice trade flows and clarifying the contributions of industrially milled, locally milled, and imported rice to consumption patterns.

Comment (Lines 357-379): Ok, yes, this is exactly what I am referring to above. These types of considerations need to be more directly integrated into your analysis (for all food vehicles, not just rice) to the extent possible (at a minimum, exclude consumption of home-produced staples, do not include whole grain versions of staples).

> **Response:** We thank the reviewer for this comment, which has driven substantial revision of the Discussion. We agree that the original Discussion under-engaged with the distinction between fortifiable and non-fortifiable consumption, and we have rewritten the relevant Discussion paragraphs to make this distinction central rather than peripheral. We respond first to the broader question of integrating these considerations into the analysis, then to the two specific exclusions the reviewer raises.
>
> Integrating fortifiable-form considerations into the analysis. As described in our response to the comment on Lines 127-129, we have restructured the objectives to separate vehicles already included in regional fortification standards (Objective 2: wheat flour, edible oil) from candidate vehicles considered for inclusion (Objective 3: rice, sorghum, millet, maize). For Objective 2 vehicles, coverage estimates are restricted to the fortifiable form (see Methods). For Objective 3 vehicles, the analytical target is total consumption as a demand signal, because the policy question (i.e., whether infrastructure investment in fortifying these grains is warranted) depends on aggregate demand rather than on current industrial output.
>
> The rewritten Discussion paragraphs carry this distinction through to the policy implications. The new Discussion paragraph on rice explicitly distinguishes imported rice fortified at the border from domestically produced rice requiring consolidation of small- and medium-scale milling, notes that "populations that consume the most rice may have limited access to a fortified product unless capacity for supply chains and milling infrastructure can be

strengthened," and commits to future research clarifying the contributions of industrially milled, locally milled, and imported rice. The revised Discussion paragraph on candidate vehicles names industrial milling and food-technology capacity as remaining at the research stage, and explicitly reports the recent finding that maize reach averaged 73% but only 37% is fortifiable, which is precisely the feasibility gap the reviewer points to.

For the two specific exclusions presented by the reviewer in this comment:

- **Home-produced staples:** For Objective 2 vehicles, home production is by definition outside industrial supply chains and therefore does not enter the fortifiable-form coverage estimate. For Objective 3 vehicles, home production is a meaningful consumption channel (particularly for sorghum and millet in the Sahel) and excluding it would distort the demand signal that motivates considering these grains for future inclusion.
- **Whole-grain versions of staples:** For candidate vehicles, the analytical target is consumption demand, which exists regardless of current processing form. The implication that fortification at scale would require shifting whole-grain consumption into industrially milled forms is now made explicit in the revised Discussion paragraph on candidate vehicles ("fortification of sorghum and millet at scale would depend on industrial milling and food-technology capacity that remain at the research stage in the region"), with the closing sentence committing to validating food technology and developing industrial milling capacity in the region. Excluding whole-grain consumption from the demand estimate would eliminate the very signal that justifies pursuing this technical pathway.

Comment (Lines 566-587): The subtitle of this section is “Access to fortifiable staple foods indicators”, but what you describe here is access to staple foods (even including home-produced staples!), definitely not fortifiable staple foods. As I’m sure you know, access to a staple does not equal access to a fortifiable staple. Maize flour is an obvious example of this in many countries (e.g., GFDx shows that just 10% of maize flour in Togo is fortifiable). In Senegal, most of the locally milled rice is at very small scale and is therefore not feasible for fortification (87% according to this 2019 landscape analysis: https://www.nutritionintl.org/wp-content/uploads/2019/10/WFP-Lanscape-Analysis-Rice-Fortification-in-Senegal.pdf). And I would be surprised if the situation was not similar for millet and sorghum for many of these countries. You claim repeatedly in the introduction (and the title) that your analysis is linking nutritional vulnerability with access to fortifiable staples. But what this analysis misses (which may lead to very misleading suggestions for appropriate food vehicles to meet the micronutrient needs of the most vulnerable), is that access to fortifiable staples is often the lowest among the poorest, most rural (and often also the most nutritionally vulnerable) populations.

Response: We thank the reviewer for this comment, which captures two related concerns with our original treatment of staple food vehicles. First, our indicators measured consumption of any form of each staple, when the

manuscript's framing implied access to the fortifiable form. Second, even with a sharper indicator, consumption of these staples is assumed to be concentrated in poorer and more rural households least likely to be reached by formal fortification infrastructure, raising the concern that the analysis could point toward vehicles whose reach to the most vulnerable would remain limited even after programme investment.

For the first concern, we have made modification in either the analytical approach (methods/results) or the narrative framing (introduction/discussion) to address this. For vehicles in current ECOWAS regional standards (wheat flour, edible oil), the analytical approach has been modified so that the indicators are restricted to the market-purchased share of consumption as a proxy for the industrially processed, fortifiable form. Home-produced consumption is excluded by construction, so reach and per capita daily consumption speak to populations reachable through formal industrial supply chains. For candidate vehicles considered for inclusion (rice, millet, sorghum, maize), the narrative framing for the interpretation of the original approach has been redefined. Total consumption (including home-produced, locally milled, and whole-grain forms) is now framed as a demand signal to inform where future programme investment in industrial milling and food-technology capacity would be warranted, not as a measure of current access to a fortifiable form.

For the second concern, we acknowledge the broader concern that the populations consuming the most of these candidate staples are often those least reached by current formal supply-chain infrastructure. We agree this concern is important, but it speaks to programme design rather than indicator construction. Our study does not presuppose a fortification technology or supply-chain pathway for these grains. The role of the consumption-demand indicator is to identify which staples warrant investment in developing such pathways in the first place. Whether a future programme reaches the most vulnerable depends on vehicle selection, technology choice, supply-chain investment, and distribution structure, where all of which sit downstream of this demand mapping analysis. Removing home-produced and locally milled consumption from the indicator would assume that future programmes cannot reach those households, and would erase the very evidence needed to design programmes that can. The revised Discussion engages this directly: the rice paragraph notes that supply-chain and milling infrastructure must be strengthened for the most rice-consuming populations to access fortified product, and the candidate-vehicles paragraph identifies development of fortification technology and infrastructure as the next required step for these grains.

Regional-national policy logic

Preface: We thank the reviewer for their careful attention to our study's regional-national policy logic. As the reviewer notes, the original manuscript invoked regional standards without making the underlying architecture sufficiently explicit. To address this, we have rewritten the relevant passages of the introduction and the policy implications section of the discussion so that this architecture is now the organizing frame.

The regional-national policy architecture in West Africa has two layers. The regional layer (e.g., ECOWAS, AES) provides a common reference which acts similarly to the role that other global guidance provides, i.e., suggesting candidate vehicles, micronutrient parameters, and a shared monitoring framework. Its function is to enable intra-regional trade, give large industry and small- and medium-scale millers compliance pathways suited to their scale, and make cross-country results comparable. The national layer (e.g., state governments) selects which vehicles to mandate, calibrates fortification levels to country-specific dietary patterns and vulnerability, and handles implementation and enforcement. The two layers operate at different tiers of governance where the regional standard sets the operating space, and national decisions populate it.

The scope of this study focuses on the regional layer, but our results connect to the whole of the architecture directly. Cross-country variation in vulnerability profiles and vehicle reach shows that no single national configuration fits the entire region. The overlap in vulnerability profiles, shared candidate vehicles, and intra-regional trade flows is what justifies the regional layer. It allows national choices to differ without the regional system fragmenting. The reviewer's individual comments are addressed below within this framing.

Comment (Lines 78-81): What is the current design, and what about it lacks adaptability, specifically? Spelling this out will help readers understand the relationship between regional standards/coordination and national fortification action.

> **Response:** We thank the reviewer for flagging this point on the design of the regional-national policy pathway for clarity. We expanded the current design of the regional-national policy pathway explicitly and acknowledge that "lacks adaptability" was imprecise and have removed the phrase. The revised introduction now states the current design more directly: "Fortification policy in West Africa is structured across two hierarchical layers, in which regional standards establish the parameters within which national policy is set. Regional standards specify candidate vehicles, micronutrient parameters, and a shared monitoring framework, enabling intra-regional trade and compliance pathways across mill scales. National governments select which vehicles to mandate, calibrate fortification levels to country-specific dietary patterns and vulnerability, and handle implementation and enforcement."

Comment (Lines 81-85): Saying that regional coordination needs to promote common standards and also support countries to adapt seems a bit contradictory. Are you saying harmonized/common regional standards are necessary or that countries should set their own standards based on dietary patterns? This is the challenge with regional standards, right? Those standards might not be suited to meet the needs of individual countries, and if individual countries adapt them to country-specific standards, then the benefits of regional harmonization (e.g., ease of trade and monitoring/regulation) are no longer fully achievable.

> **Response:** We thank the reviewer for raising this point. We agree that common regional standards and country-specific adaptation pose a contradictory narrative, and the original manuscript did not present the interaction between regional guidance and national policy clearly enough. By assigning different elements to each layer, the design preserves the trade and regulatory benefits of harmonization at the regional level while still allowing fortification mandates and levels to be calibrated to country conditions.
>
> In the revision, we have separated the two layers so that it is explicit which elements are harmonized and which are adapted. The regional layer harmonizes the elements where common standards support trade, regulation, and comparability across countries: "candidate vehicles, micronutrient parameters, and a shared monitoring framework." The national layer adapts the elements that need to fit country-specific contexts: "National governments select which vehicles to mandate, calibrate fortification levels to country-specific dietary patterns and vulnerability, and handle implementation and enforcement."

Comment (Line 183): Just regional standards? What about national standards (the two do not fully align for all of these countries, right?).

> **Response:** We thank the reviewer for this point. The reference to regional standards at this line is intentional. Our framework classifies vehicles based on their position in the regional layer: vehicles already included in guidance

for regional standards (wheat flour and edible oil) versus candidate vehicles considered for inclusion. The line the reviewer flags refers to vehicles already included in regional standards, because the analytical question this study addresses operates at the regional standard-setting layer. We acknowledge that national standards do not fully align with regional standards across the ten countries, and that some countries have mandated additional vehicles at the national level. This is outside the scope of our framework, which is designed to inform the regional layer rather than to catalogue national-level variation. The revised introduction states this scope directly.

Comment (Line 276-278): I'm having a hard time following the regional-national pathway here. Can you please explain how regional standards would help countries prioritize those most appropriate vehicles and fortification levels according to country-specific dietary patterns and vulnerability contexts? Where do the regional standard fit in, and what, specifically, about your results, suggest a role for regional standards (I'm not saying there is not a role, I just think you need to explain what you mean here, including how your results fit into what you are suggesting).

Response: We thank the reviewer for flagging this point. We agree that the original sentence did not adequately explain how the regional layer connects to our results. We have revised the discussion paragraph to make this connection explicit: "The cross-country variation in vulnerability profiles and vehicle access observed here shows that no single national configuration fits the entire region, while the overlap in candidate vehicles across countries (e.g., rice across most of the region, millet and sorghum across the Sahel) points to where regional-level perspectives in fortification pathways is justified. The role of the regional standards is therefore to maintain a shared menu and parameter set, with national governments calibrating specific mandates and fortification levels to country-specific dietary patterns and vulnerability profiles."

Comment (Lines 405-413): Here again, please explain what about your results suggest the need for regionally aligned standards. And are you suggesting here that the standards for all food vehicles be the same in the region (which micronutrients and fortification levels) but then countries choose which food vehicles to mandate? Again, I'm not quite following what the suggestions is here and this section needs to be reworked a bit for clarity. Also, small point, but I'm not sure what, "market surveys require a regional reference standard" means.

Response: We thank the reviewer for this point. We agree that the original passage did not clearly explain how the regional layer connects to our results, what is harmonized versus what is national, or what was meant by "market surveys require a regional reference standard." We have revised the passage to address each of these. The revised passage grounds the framework in established global and regional references and applies it to our findings:

"These findings reinforce the framework laid out in the WHO/FAO Guidelines on Food Fortification with Micronutrients, in which harmonized international or regional reference parameters support trade and regulation while national decisions determine which vehicles to mandate and calibrate fortification

levels to local contexts. In West Africa, harmonisation efforts through regional governing institutions have produced meaningful progress, though political-economy constraints continue to shape pathways for further alignment. Our findings suggest that regional fortification guidance should harmonize the elements that support intra-regional trade, regulatory infrastructure, and cross-country comparability such as the candidate vehicle menu, the micronutrient parameters, and a shared monitoring framework. The national policy determines which subset of those vehicles to mandate and calibrates fortification levels to country-specific dietary patterns, production systems, and vulnerability profiles. The diversity of consumption patterns across West Africa documented in this study underscores the value of an expanded regional menu of candidate vehicles and parameters, so that countries can select the vehicles best suited to their context."

Sorghum & millet fortification

Preface: We thank the reviewer for their careful attention to our treatment of parameters reflecting millet and sorghum fortification. As the reviewer notes, the original manuscript did not clearly distinguish current consumption from current fortifiability for these grains. To address this, we now present millet, sorghum, and maize as advocacy cases for future infrastructure investment to develop these vehicles.

Under this framing, HCES data capture grain consumption in any form depending on the grain. Reach is thus reported as the share of households consuming the grain, which signals where food demand exists that a future fortification pathway could meet. Consumption is thus reported as the quantity of grain consumed regardless of form, indicating the scale of demand that would justify investment in food technology and industrial milling capacity. We have revised the Results and Discussion text to ensure that the paper does not claim that millet, sorghum, or maize reach vulnerable populations through fortification today. The reviewer's individual comments are addressed below within this framing.

Comment (Lines 272-274): Here is an example of where I think this approach may generate misleading results. Do we really think the poorest populations in these countries are consuming industrially processed millet and/or sorghum flour? It seems doubtful.

> **Response:** We thank the reviewer for flagging this point. We agree that the original phrasing risked implying that the poorest populations are currently consuming industrially processed millet or sorghum flour, which is not what our analysis supports. As described in the preface to this section, our intent is to characterise demand patterns that could justify future infrastructure investment in millet and sorghum as fortification vehicles, not to claim that fortified forms of these grains currently reach vulnerable populations.
>
> We have revised the sentence to make this distinction explicit: "Beyond wheat flour, other staple grains present unique opportunities for fortification in West Africa, with rice showing widespread consumption and high per capita daily intake among the population in need in most countries and millet and sorghum showing consumption concentrated in economically vulnerable populations and geographies with elevated vulnerability to inadequate micronutrient intake in the Sahel and certain regions of other countries." The revised phrasing speaks to where demand exists for a future fortification pathway to anchor, not to current industrial supply.

Comment (Line 393): Please also comment on the likelihood that millet or sorghum are industrially milled at any significant scale in these countries (and hence whether millet or sorghum flour fortification would, in any way, be feasible). My assumption is that industrial milling is quite uncommon, but I'm happy to be proven wrong.

> **Response:** We thank the reviewer for raising this point. We agree with the reviewer's assumption, where industrial milling of millet and sorghum is uncommon at significant scale in West Africa, and fortification of these grains in their current consumption forms would face significant constraints without further development of industrial milling and food-technology capacity. As described in the preface to this section, this is why we have positioned millet, sorghum, and maize as candidate vehicles whose consumption patterns make the case for future infrastructure investment.
> We have revised the Discussion paragraph to make this position explicit. The revised paragraph states directly that "fortification of sorghum and millet at scale would depend on industrial milling and food-technology capacity that remain at the research stage in the region," and identifies validation of food technology and development of industrial milling capacity as required next steps.

Comment (Table S10): I am not following how maize, millet, and sorghum are fortifiable (as distinct from maize flour, millet flour, and sorghum flour – these are separate food items on most of the food lists). Biofortifiable, yes, but household consumption of whole grain maize, sorghum, and millet is not fortifiable. Please clarify or correct this.

Response: We thank the reviewer for raising this point and want to clarify our treatment of these grains. We agree fully that whole-grain maize, millet, and sorghum cannot be industrially fortified in the same way that other flours can.

The reason these grains appear in Table S10 is that we positioned them in this paper as candidate vehicles for future fortification investment, not as vehicles currently operating in their consumed form. As described in the preface to this section, the analytical question for candidate vehicles is whether existing consumption patterns are concentrated enough to justify future investment in the industrial milling and food-technology capacity that would make fortification feasible. Under this framing, total consumption of the grain (in any form) is the relevant parameter, because it signals the scale of demand that could anchor a future fortification pathway.

We have revised the Discussion paragraph that interprets these results to state this explicitly: *"fortification of sorghum and millet at scale would depend on industrial milling and food-technology capacity that remain at the research stage in the region."*

Comment (Line 603): Millet flour and sorghum flour, correct?
Comment (Line 552 and line 559): Maize flour instead of maize here, correct?

Response: We thank the reviewer for flagging these points about sorghum and millet flour (similar with maize). We have retained the original terminology. Under the revised Objective 3 framing, these sections describe consumption of the commodity as a candidate vehicle, not its processed flour form, in order to describe demand for candidate vehicles through consumption patterns.

Micronutrient-specific health pathways

Comment (Lines 47-50): The way this sentence is written implies that all five of these micronutrients impact immune and cognitive function, pregnancy outcomes, and productivity, which is not the case (e.g., no well-established links between vitamin A and productivity or pregnancy outcomes). Consider revising to accurately describe these relationships instead of lumping them all together.

> **Response:** We thank the reviewer for flagging this point. We agree that the original sentence implied universal attribution across all five micronutrients of immune and cognitive function, pregnancy outcomes, and productivity, which the literature does not support.
>
> We have removed the original lumped sentence and replaced it with a differentiated description of mortality pathways grouped by micronutrient cluster: "Vitamin A and zinc deficiencies elevate child mortality risk through impaired immune function and infection susceptibility, while iron, folate, and vitamin B12 deficiencies contribute to maternal mortality and adverse birth outcomes via anaemia." Productivity has been dropped from the intro entirely. Fuller pathway differentiation, with citations by micronutrient, is provided in the corresponding Methods section.

Comment (Line 503-505): Is there a well-established pathway from B12 deficiency to mortality? If yes, please provide a citation (for B12 as well as for the other micronutrients).

> **Response:** We thank the reviewer for raising this point. Yes, vitamin B12 deficiency contributes to maternal and infant mortality through the anaemia pathway. Vitamin B12 deficiency clinically manifests as megaloblastic anaemia via impaired DNA synthesis (Stabler 2013; Green 2017), and severe anaemia in pregnancy elevates the risk of maternal mortality from haemorrhage and adverse birth outcomes (Rahman 2016).
>
> We have revised the Methods paragraph to make this pathway explicit and added citations for each of the five micronutrients. The relevant passage now reads: "Iron, folate, and vitamin B12 deficiencies elevate maternal mortality risk via severe anaemia and contribute to adverse birth outcomes [Rahman 2016]. Iron deficiency is the leading cause of anaemia globally [Lopez 2016], while folate and vitamin B12 deficiencies clinically manifest as megaloblastic anaemia through impaired DNA synthesis [Green 2017; Stabler 2013]. Folate deficiency additionally contributes to neonatal and infant mortality through increased risk of neural tube defects [Blencowe 2010]."

Comment (Lines 505-507): This line needs to be reviewed and corrected/nuanced for accuracy, as it currently reads as though each of these five micronutrients have the same consequences (impaired linear growth, cognitive development, and functional capacity). Please also add citations for each of the pathways you are claiming (by micronutrient and outcome).

Response: We thank the reviewer for raising this point. We agree that the original sentence read as though each of the five micronutrients drives each of the listed developmental outcomes, which is not the case.

We have revised the sentence in three ways. First, "functional capacity" has been removed entirely, consistent with the parallel revision in the Introduction (Comment on Lines 47-50 above) where "productivity" was likewise dropped. Second, "contribute to" has been changed to "have been linked to" to avoid the implication of universal attribution. Third, the primary rationale for micronutrient selection now sits in the mortality-pathway sentences immediately preceding this one, where attribution by micronutrient and citations by pathway are explicitly provided. The remaining developmental claim is framed as supporting context, drawing on the Lancet Maternal and Child Nutrition series (Black 2013), which synthesises the literature on micronutrient deficiencies and developmental outcomes across this set of micronutrients.

The revised sentence reads: "These micronutrient deficiencies have also been linked to longer-term developmental outcomes, including impaired linear growth and cognitive development (Black et al., 2013), aligning with priorities reflected in the United Nations Sustainable Development Goals."

Methodological assumption documentation

Comment (Lines 494-496): Please describe, specifically, how you identified and corrected outliers.

> **Response:** We thank the reviewer for raising this point. We agree that the original Methods text did not provide sufficient detail on the outlier procedure and have revised the Data preparation subsection accordingly. The revised text reads:
>
> "To address extreme values likely reflecting recall errors, outliers were identified separately for each country and food item among households consuming that item: reported quantities were log-transformed, and any value exceeding the consumer mean plus four standard deviations on the log scale was flagged. Flagged values were replaced with the geometric mean of consumer-reported quantities for that food item in that country."

Comment (Lines 532-533): Please add a few lines here with additional detail on how AFEs were calculated for each county. What was the basis for energy requirements? Were the same energy requirements used for each country? How were breastfeeding children and pregnant and lactating women dealt with?

> **Response:** We thank the reviewer for this comment. We have expanded the Methods to specify the basis for energy requirements, confirm that the same requirements were applied uniformly across all ten countries, and describe how women of reproductive age in households with a child under two and the children themselves were handled. Pregnancy status is not collected in any of the underlying HCES, and we have clarified how this was addressed. The revised passage now reads:
>
> "Household micronutrient supply was derived by aggregating micronutrient values from all reported foods and dividing the total by household size standardized to adult female equivalents (AFEs). The primary assumption in the AFE approach is that household micronutrient supply is distributed across household members proportionally according to each member's mean daily energy requirements [68] estimated using information on age and sex of household members from the HCES household roster. Mean daily energy requirements were drawn from the FAO/WHO/UNU Human Energy Requirements report and applied uniformly across all ten countries. AFEs are defined as the ratio of each household member's mean daily energy requirement to that of a reference non-pregnant non-lactating 18–29-year-old woman [69]. In households with a child under two years of age, two adjustments were made: the woman of reproductive age was assigned the energy requirement of a breastfeeding woman, and the child's energy requirement was reduced to exclude the contribution of breastmilk. All other women of reproductive age (including those who may have been pregnant, since pregnancy status is not captured in HCES rosters) were assigned the energy requirement of a non-pregnant non-breastfeeding woman."

Comment (Line 541-543): I'm not quite following this line, "The overall MPI was estimated by combining the simple average of the probability of inadequacy for all

five micronutrients." How were they combined? Do you mean "taking" the simple average rather than combining the simple average?

> **Response:** We thank the reviewer for flagging this point. The reviewer's interpretation is correct, and the overall MPI is calculated by taking the average of the probabilities of inadequacy across the five micronutrients, and the original wording was imprecise. The Methods (Vulnerability indicators) has been revised to read: "The overall MPI was calculated as the arithmetic mean of the probabilities of inadequacy across the five micronutrients."

Comment (Table 1): Here, too, I'm not sure what "combining" the simple averages means. And if it means taking the simple average, the value does not seem correct for Burkina (average would be around .67?).

> **Response:** We thank the reviewer for raising this. The wording fix described in our response to L541-543 addresses the "combining" ambiguity but not the arithmetic discrepancy raised here. On the latter: the reviewer's calculation, or averaging the five per-micronutrient percentages displayed for Burkina ([0.94 + 0.66 + 0.45 + 0.76 + 0.56] / 5 = 0.67), uses outputs from the cut-point approach (for Vit A, folate, B12, zinc) and the full probability approach (for iron). The overall MPI, by contrast, is the arithmetic mean of the probability-of-inadequacy values produced by the probability approach. This is a different statistical method applied to the same intake distributions. The two sets of outputs are not interchangeable, which is why averaging the displayed percentages yields 0.67 while Burkina's overall MPI is 0.64. The Methods (Vulnerability indicators) describes both approaches. We have also added a footnote to Table 1 noting that the overall MPI is computed via the probability approach and is not equivalent to the mean of the percentage-vulnerable values in adjacent columns (cut-point approach for Vit A, folate, B12, zinc; full probability approach for iron).

Expand description of surveys details

Comment (Lines 486-488): I may be wrong, but I think the Ghana HCES data are fairly substantially different from the other countries and likely required very different processing methods. If this is correct, please make that clear and describe the way the Ghana HCES were processed relative to the other surveys. I think it's important to point out that there can be wide variation in how these surveys are conducted country to country, requiring adapted methods for preparing the data for this type of analysis.

> **Response:** We thank the reviewer for this comment, transparency around the data processing is important for the replicability of this work. As indicated, Ghana was different from the other surveys in both the recall period and type of household consumption data collected. The Ghana Living Standards Survey collected household food expenditure data via food diary method over an 18-day period, whereas the ECHVM and Nigeria Living Standards Survey collected household food consumption quantities through a 7-day recall. We have added two columns in Table S10 which now indicate the recall period and the food data type (e.g., food expenditure vs. consumption quantities). We have also added the following text to indicate the difference in data processing required for the Ghana Living Standards Survey:
>
> *For the ECHVM surveys and Nigeria Living Standards Survey, reported quantities of food items were converted into metric units using provided conversion factors and adjusted to exclude inedible parts of foods (e.g., the skin of a banana), to generate estimates of daily apparent household consumption. For Ghana, household food expenditure estimates were collected rather than food consumption quantities. Consumption quantity estimates, previously derived from these expenditure data by the UC Davis MINIMOD team, were used as the inputs for this analysis and were subsequently adjusted to exclude inedible portions of foods as described above.*

Comment (Line 492): Were conversion factors provided for all surveys? Ghana?

> **Response:** We thank the reviewer for this comment, and we agree it is important to maintain the highest level of clarity. Please see the response to the above comment where we have addressed this.

Comment (Table S7): This table provides great context. Please add the survey year for each country.

> **Response:** We thank the reviewer for flagging this. The table has been updated with a column for both the data source and survey year for each country.

Analysis approach

Comment (Line 419): I think you should probably also acknowledge the fact that although the data that you are using does allow for quantitative modelling of the potential impacts of fortification on micronutrient inadequacy, you have chosen not to do so here and that setting fortification standards (either establishing or adapting) should really be informed not only by which food vehicles are consumed in sufficient quantities by populations with micronutrient inadequacies, but also by directly modelling the impact of fortification of individual and combinations of food vehicles on the gap between intake and requirements, the prevalence of inadequacy, and the risk of high intakes.

> **Response:** We thank the reviewer for raising this point. We agree that the HCES data underlying this analysis could in principle support quantitative modelling of fortification's impact on intake gaps, the prevalence of inadequacy, and the risk of high intakes, and that setting fortification standards is ultimately informed by such modelling alongside descriptive characterization of vehicle reach and consumption. We did not pursue impact modelling here because our objective was the upstream step: characterizing which vehicles align with vulnerability in ways that warrant prioritization within and across countries. We have revised the Limitations paragraph to acknowledge this scope choice and to flag impact modelling as a logical next stage. The added passage reads:
>
> "…the underlying data also permit quantitative modelling of fortification's impact on intake gaps, the prevalence of inadequacy, and the risk of high intakes, but this analysis was scoped to the upstream task of characterizing which vehicles align with vulnerability across the region. Setting fortification standards, whether establishing new ones or adapting existing ones, should also be informed by direct impact modelling of single and combined vehicles, a logical next stage building on the priorities identified here."

Comment (Table 1): The prevalence of vitamin A inadequacy for Nigeria seems significantly higher than other estimates I've seen. I'm curious what you assumed about palm oil. This paper describes how this issue was dealt with in a similar analysis via input from a stakeholder group in Nigeria: https://cdn.nutrition.org/article/S2475-2991(24)02456-9/fulltext

Response: We thank the reviewer for raising an important point. The treatment of palm oil substantially influences vitamin A intake estimates in Nigeria, and our analysis differs from Adams et al. in how this ambiguity is handled.

In the Nigeria HCES, palm oil is recorded as a single item that does not distinguish unrefined (red, β-carotene-rich) from refined (clear, β-carotene-depleted) forms. Adams et al. addressed this through stakeholder consultation in Nigeria, concluding that the reported palm oil was predominantly red. We took a different methodological approach, drawing on quantitative consumption data from neighbouring countries where the survey instrument distinguishes the two forms, to assess whether regional patterns supported a uniform classification within Nigeria.

The bivariate maps below show subnational consumption of (A) unrefined/red palm oil and (B) refined palm oil across the region. Red palm oil consumption is geographically heterogeneous within some neighbouring countries. In Benin, for example, consumption is concentrated in the south and declines markedly moving northward. This gradient is broadly inverse to refined palm oil consumption, with northern regions (e.g., Niger) showing minimal red palm oil consumption and substantial refined palm oil use. Together, these data indicate that the form of palm oil consumed varies meaningfully across the region, and likely within Nigeria as well, making a single national-level classification difficult to substantiate on the basis of available consumption data alone.

Given this uncertainty, we elected to treat the palm oil item as refined for our primary analysis. This is a conservative assumption with respect to vitamin A intake: it yields lower modelled intake from palm oil, and therefore a higher estimated prevalence of inadequacy, than would result from assuming all palm oil is red. We view this as the appropriate default in the absence of disaggregated consumption data, and acknowledge that the true national estimate likely sits between these two bounds. We recognize that the stakeholder-informed approach taken by Adams et al. reflects a defensible alternative framing of the same underlying data limitation.

Figure A. Subnational reach and consumption quantities of **unrefined vitamin A-rich red palm oil** in EHCVM countries
Figure redacted.

[redacted]

Grey = no consumption; yellow = low reach, high quantity among consumers; blue = high reach, low quantity; green = high reach, high quantity

Minor Comments

Comments:

- **Lines 27-28:** Flagging for tense inconsistency in this sentence (analysed and then assess).
- **Line 137:** Typo (study').
- **Line 293:** Typo – missing the word "are" before updated.
- **Line 435:** Missing word (I think it should read, "…we assessed…").
- **Table 2:** Title has a small typo (two commas after rice).

  Response: We thank the reviewer for catching these typos. All identified typos and grammatical issues have been corrected, or the relevant text has been revised or removed in the course of the broader revisions.

Comments:

- **Lines 108-109:** Unclear what is meant by complementary vs alternative vehicles. What's the difference and when would you need one vs the other?
- **Line 291:** What is the difference between complementary and alternative vehicles? Do you mean complementary interventions?

  Response: We thank the reviewer for flagging this ambiguity. The text has been either deleted (first mention) or revised to refer more broadly to complementary interventions rather than distinguishing between vehicle types.

Comment (Line 224): Above rather than less than 10%, right?

> **Response:** We thank the reviewer for identifying this error. The text has been corrected to "exceeded 10%."

Comment (Lines 557-559): Hmm, Nigeria has a mandatory national standard for the fortification of maize flour so would not qualify as emerging by this definition, right?

> **Response:** We note that in the revised manuscript we have removed the "established"/"emerging" terminology in favour of "vehicles included in current regional standards" and "candidate vehicles considered for inclusion," which more precisely reflects the basis of our classification. That classification is anchored on regional rather than national standards: the ECOWAS harmonized fortification standards (i.e., wheat flour, edible oil, salt) are the only multilateral regulatory reference point applicable across all study countries. National-level mandates such as Nigeria's maize flour standard fall outside this regional framework, and maize is therefore treated as a candidate vehicle considered for inclusion. This rationale is now stated explicitly in the Methods.

Comment (Lines 629-630): What are the "operational constraints associated with vitamin A fortification of cereal vehicles"? Just off the top of my head, I know that both Nigeria and Malawi mandate the fortification of wheat flour and maize flour with vitamin A.

Response: We thank the reviewer and agree they are correct that Nigeria and Malawi mandate vitamin A in cereal flour, and our original wording was imprecise. By "operational constraints" we meant the well-documented retention losses in dry flour as vehicles (Klemm et al. 2010), the relatively higher and WHO 2022 guidance treating vitamin A in wheat flour as conditional if and only if no other vitamin A interventions are in place. These reasons contribute to why eight of the ten countries in our analysis carry vitamin A through edible oil rather than flours. We have revised the language to be more specific: "…vitamin A was added to the vehicle-specific MPI for wheat flour, rice, and maize to represent a scenario assuming full adherence to WHO fortification guidance, setting aside the retention and normative-guidance considerations that lead many West African countries to carry vitamin A primarily through edible oil rather than cereal vehicles [Kelmm et al., 2010; WHO, 2022]).

Comment (Table 3): Small point, but the inclusion of "beneficiary population size" in the title does not seem accurate. Percent covered/reached is very different than the size of the population (e.g., if the beneficiary population is 50% in Nigeria, that population size would of course be extremely different than the size of a 50% beneficiary population in Guinea-Bissau).

Response: We thank the reviewer and agree that "size" was imprecise given that the table presents proportions. The title has been revised to replace "size" with "share": "Public health potential of each vehicle based on the beneficiary population share and characteristics of vehicle consumption amongst potential beneficiaries."

Comment (Remarks on code availability): I was able to access the code but did not review it in detail.

Response: We thank the reviewer for confirming access to the code repository.

Comment (Figure 2): Where is all that dietary folate coming from in Niger? You don't actually need to answer this question in the paper – it's just a bit surprising.

Response: For the reviewer's reference: folate intake in Niger derives predominantly from grains, roots, and tubers (74%) (Figure A), with millet alone contributing 41% to total intake on average (Table A).

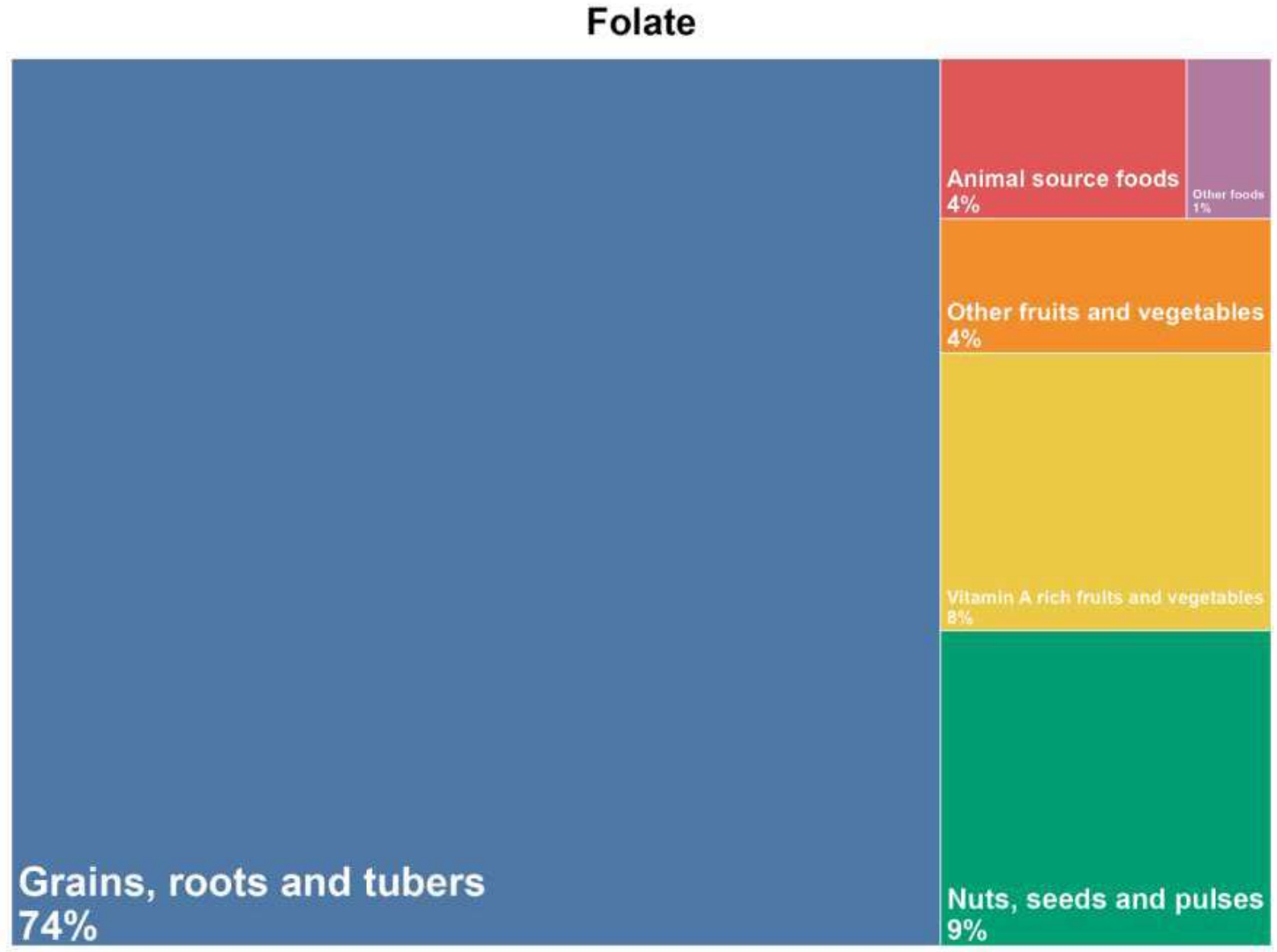

Figure A. Dietary sources of folate in Niger (food group contributions to total folate intake in Niger EHCVM 2021/2022)

Table A. Dietary sources of folate in Niger (top 10 food item contributions to total folate intake in Niger EHCVM 2021/2022)

Food item	Mean folate contribution to total intake, %
Millet	41
Pasta	13
Sorghum	6
Cowpea / Dried beans	5
Corn kernels	5
Dry okra	3
Soumbala (African mustard)	2
Imported long grain rice	2
Local or imported wheat flour	1
Wheat semolina	1

Reviewer #3

General perspectives

Comment: This is an interesting and useful paper that provides important evidence that was previously missing in the LSFF research literature. The analysis is rigorously specified and includes very good data visualizations that help to present the findings in a succinct way. This paper will prove useful for policy-focused researchers and practitioners. Most of the edits requested below are very small and aimed at improving the flow of the presentation.

> **Response:** We thank the reviewer for the positive assessment and for the careful reading of the manuscript. We have addressed each of the specific comments below.

Major comments

Differentiated analysis of current vs. candidate fortification vehicles

Comment (Lines 583 – 585): If reach and consumption are measured using all forms of the food consumed, then doesn't this result in an over-estimate of the potential consumption of the fortified food vehicle? That is, the portion of consumption that is home produced is almost certainly not fortifiable. You may have good reason to keep this way of measuring reach and consumption, rather than limiting it to only market acquired foods. This is mentioned at lines 422 -425. However, please provide a bit more explanation on why this is the best approach and what the consequences might be.

> **Response:** We thank the reviewer for raising this point. In response to this comment and additional related comments raised by Reviewer 2, we have restructured the analysis so that reach and consumption are no longer measured uniformly across vehicles.
> Vehicles are now treated according to the analytical question each is being used to answer. For wheat flour and edible oil (the two vehicles included in current regional standards with established industrial supply chains) reach and consumption are restricted to the fortifiable form, defined as market-acquired and industrially processed product. Home-produced and informally milled quantities are now excluded in these revised results. This addresses the over-estimation concern the reviewer raises directly, since the indicators now reflect only the share of consumption that could plausibly be reached by the existing fortification pathway. For rice, millet, sorghum, and maize (vehicles considered as candidates for future fortification) reach and consumption are measured using total consumption, irrespective of source. For these vehicles, the analytical question is not "how much of current consumption is reachable by an existing programme" but "what is the magnitude of staple demand that could justify and shape new fortification infrastructure." Restricting to market-acquired forms in this case would understate the policy-relevant demand signal and obscure the public health potential that domestic milling consolidation or import-based fortification could unlock.
> This bifurcation is now described explicitly in the revised Methods (Subsections called "*Reach and consumption of vehicles in current regional standards*" and "*Consumption patterns of candidate vehicles considered for inclusion*") and reflected in the Results, which present the two vehicle classes in parallel sections with footnotes on each table clarifying which definition applies. The Discussion also now notes the consequence of the candidate-vehicle definition: estimated public health potential for rice, millet, sorghum, and maize represents an upper bound that depends on the future fortifiability of the supply, which is a programmatic and infrastructural question that is discussed supported by programmatic reports of pilots that have explored these programmes for scale-up.

Definition of key terms

Comment (Line 98): A very brief definition or introduction of the term “vehicle” would likely help readers who are not familiar with LSFF.

> **Response:** We thank the reviewer for this suggestion. We have integrated a brief definition of "fortification vehicle" into the opening sentence of this introduction paragraph. The revised text reads: "Large-scale food fortification (LSFF), which involves adding micronutrients to widely consumed staple foods and condiments (fortification vehicles) during industrial processing, is a cost-effective, population-level intervention that, when effectively implemented, consistently improves micronutrient intake."

Comment (Lines 179 – 220): A very brief definition of the term “reach” would be useful here. I understand that the journal format puts the Methods section later, but a shortened version of the full definition from lines 568 to 571 would improve clarity as this term is not self-evident in the way that “consumption” is. This is already done (and helpful) for the definition of “coverage” at line 223-224.

> **Response:** We thank the reviewer for this observation. We agree that "reach" is not self-evident and have added a brief inline definition at first use in the Results, consistent with how "coverage" is defined. The sentence now reads: “For wheat flour and edible oil, percent reach (the percentage of the population consuming any amount of the staple; %) and…”

Comment (Lines 168 – 178): It would be more precise and clear to say “Vulnerability to inadequacy is higher at lower socioeconomic positions”, or even “Poor households are more vulnerable to inadequacy.” Firstly, since this is a cross-sectional analysis that doesn’t observe changing income status across a panel of households, and secondly, as the phrasing at 170-171 is ambiguous about the direction of change.

> **Response:** We thank the reviewer for suggesting this clarification and agree that the recommended phrasing is more precise and clear. We have now changed the sentences in this paragraph to reflect this more direct language. For example, the first sentence in this paragraph now reads: “Disaggregating the overall MPI by wealth quintiles showed that poorer populations are more vulnerable to inadequacy.”

Comment (Multiple places): the term “Socioeconomic position by quintile” is perhaps unnecessarily long when these are really “expenditure quintiles” (or even “income quintiles” give longstanding usage in the literature). You are free to disagree if you strongly prefer this term, but since the terms “poor” and “wealthy” are also used elsewhere in the text, I suggest streamlining here as well.

> **Response:** We thank the reviewer for this suggestion, and we agree that "socioeconomic position by quintile" is unnecessarily long and have streamlined the terminology throughout. We have replaced this term with

"poorest" and "wealthiest" for comparative language where appropriate. The term is defined once in the Methods and used consistently thereafter. Representative examples of the revised language are shown below:

- “with higher values observed in poorer subgroups as observed in…”
- “… showed increasing vulnerability with poorer populations”
- “indicating that poorer expenditure quintiles was associated with higher sorghum reach”

Methodological assumption documentation

Comment (Lines 527 – 533): Please provide some brief discussion of whether the AFE conversion approach accounts for potential reporting error in the dietary recalls performed for household surveys. Perhaps the AFE approach is sufficiently equivalent to energy adjustment, but readers familiar with nutrition assessment will want to know whether this is the case.

> **Response:** We thank the reviewer for raising this point. The AFE approach addresses variation in energy requirements across household members but is not equivalent to energy adjustment in the reporting-error sense used in individual dietary recall methods. We have added a brief clarification to the Methods noting that this is a recognised limitation of the HCES-based nutrient supply framework, and that MPI estimates should be interpreted as population-level proxies for vulnerability to inadequate intake rather than precise measures of individual consumption. The additional text reads as follows:
> "*The AFE approach addresses variation in energy requirements across household members but does not correct for household-level reporting error. This distinction is a recognised limitation of the HCES-based nutrient supply framework, and MPI estimates should be interpreted as population-level proxies for vulnerability to inadequate intake rather than precise measures of individual consumption.*"

Comment (Lines 549 – 541): This is a very reasonable approach. I wonder, though, if it would be possible or useful to allow the bioavailability assumption to vary across households depending on the composition of reported diets. Households with higher consumption of animal-source foods would be subject to a higher assumed bioavailability of iron and zinc. Variation in consumption of animal-source foods is likely to be large in several countries, which would further exaggerate the variation in MPI for iron and zinc.

> **Response:** We thank the reviewer for this suggestion. To assess sensitivity to the bioavailability assumption, we re-estimated vulnerability to inadequate iron and zinc intake using more favourable assumptions: 15% absorption for iron (WHO, 2006) and requirements corresponding to a refined diet for zinc (Allen et al., 2020). Results are presented in **Table S7.**
> In the Methods, we added: "*As a sensitivity analysis, vulnerability to inadequate iron and zinc intake was re-estimated using more favourable bioavailability assumptions: 15% absorption for iron and requirements corresponding to a refined diet for zinc.*"
> In the Results, we added: "*A further sensitivity analysis using more favourable bioavailability assumptions for iron (15% absorption) and zinc (refined diet) reduced estimated vulnerability by approximately 20 and 30 percentage points, respectively, though high vulnerability and the socioeconomic gradient persisted across all countries (**Table S7**).*"

Table S7. *Sensitivity analysis of vulnerability to inadequate iron and zinc intake assuming bioavailability of 15% for iron (REF WHO 2006) and a refined diet for zinc (REF Allen et al., 2020)*

		Percent vulnerable to inadequate intake			
Country	Socioeconomic position (by quintile)	Iron assuming 5% bioavailability (%)	Iron assuming 15% bioavailability (%)	Zinc assuming an unrefined diet (%)	Zinc assuming a refined diet (%)
Benin	National	57	35	77	46
	1 (poorest)	63	42	82	56
	2	60	37	82	50
	3	59	35	81	50
	4	56	32	76	42
	5 (wealthiest)	51	28	71	36
Burkina Faso	National	56	36	76	48
	1 (poorest)	66	47	85	66
	2	63	42	83	54
	3	57	36	77	46
	4	51	30	69	38
	5 (wealthiest)	45	25	64	31
Cote D'Ivoire	National	65	44	79	45
	1 (poorest)	74	54	89	62
	2	67	46	82	53
	3	63	42	81	46
	4	62	39	77	41
	5 (wealthiest)	61	38	74	34
Ghana	National	62	47	64	45
	1 (poorest)	77	66	81	64
	2	65	51	70	50
	3	61	44	63	40
	4	52	34	55	30
	5 (wealthiest)	43	25	44	19
Guinea-Bissau	National	74	53	81	56
	1 (poorest)	72	53	82	62
	2	74	54	82	57
	3	72	50	80	52
	4	77	56	82	57
	5 (wealthiest)	76	54	80	52
Mali	National	73	49	81	49
	1 (poorest)	80	60	92	69
	2	76	54	88	57
	3	73	49	83	49
	4	70	44	79	44
	5 (wealthiest)	68	44	77	41
Niger	National	58	34	81	43
	1 (poorest)	54	32	82	44
	2	52	29	81	39
	3	53	31	78	38
	4	57	33	79	32
	5 (wealthiest)	60	35	76	35
Nigeria	National	55	31	75	43
	1 (poorest)	79	55	93	76
	2	62	34	85	54
	3	53	27	76	42
	4	45	21	70	32
	5 (wealthiest)	38	19	58	26
Senegal	National	72	47	81	48
	1 (poorest)	81	60	89	64
	2	73	49	83	50
	3	71	45	82	47
	4	69	42	78	40
	5 (wealthiest)	60	32	66	26
Togo	National	69	48	85	61
	1 (poorest)	72	51	87	66
	2	72	51	89	66
	3	71	50	88	68
	4	68	47	87	65
	5 (wealthiest)	65	43	86	60

Minor comments

Comment: In some places the writing is not easy to follow, mainly due to sentence length, the combination of multiple infinitives, and long subordinate clauses. Some targeted revision would improve the paper's readability significantly. For example (not an exhaustive list):

- **Lines 60 – 53:** this sentence would be more readable if the final clause (line 6) were a separate sentence.
- **Lines 72 – 74:** restructuring this sentence so that it has fewer subordinate clauses would be helpful (especially the final clause at line 74).
- **Lines 81-85:** similar issues as in the above, with too many infinitives ("to promote", "to adapt", "to strengthen") making the primary point hard to track.
- **Lines 96-100:** similar issues as in the above.

  Response: We thank the reviewer for this feedback and agree that several sentences were unnecessarily long or structurally complex. We have revised the manuscript throughout to improve readability, focusing on three recurring issues: sentences exceeding 50 words, the combination of multiple infinitives, and long subordinate clauses.

Comment (Line 435): "In conclusion, assessed…" **–** appears to be missing "we" or another subject of the verb "assessed"**.**

> **Response:** We thank the reviewer for raising attention to this type and we have corrected it.

Comment (Table 2 title): extra comma after rice

> **Response:** We thank the reviewer for raising attention to this typo and we have corrected it.

Diversified food fortification portfolios can enhance coverage to micronutrient vulnerable populations in West Africa

-POINT-BY-POINT-

Table of Contents

Final reviewer comments ***2***

Remarks on code availability ***3***

Final reviewer comments

Reviewer #1: Authors have sufficiently addressed my comments.

Response: We thank the reviewer for their contributions.

Reviewer #2: The authors have done a very nice job revising their paper in response to my feedback and in providing comprehensive responses to each of my comments. While I'm still a little uncomfortable with including home-produced and whole grain consumption in the candidate fortification vehicles calculations, the authors' reframing (bifurcation of objective 2) and improved discussion around fortifiability/need for investments in milling capacity, food technology, etc to make the leap from existing demand to fortification, are a reasonable way to get around this issue for this specific analysis. I think the paper is ready for publication. Nice work!

Response: We thank the reviewer for their contributions.

Reviewer #3: The authors have carefully responded to my comments as well as those of the other reviewers, making improvements that clarify the methods and interpretation of the paper. I am confident that the revised paper meets the requirements for publication in Nature Food.

Response: We thank the reviewer for their contributions.

Remarks on code availability

Comment: The code is not formatted in such a way that allows for immediate reproducibility, as the underlying household survey data are not directly linked (which is appropriate for avoiding data use violations), and as the file structure is not set up within the scripts. Depending on the journal's requirements, a somewhat more detailed README would be helpful for 1) making it clear where the household survey data should be downloaded, and 2) how someone interested in reproducing the results should set up their own directories to run all scripts. However, the code appears to be complete and readily usable.

Response: We thank the reviewer for confirming that the code is complete and readily usable, and for the recommendation on documentation. We agree that the underlying household survey microdata cannot be redistributed within the repository, as this would breach the data providers' terms of use. We have expanded the README to address both points raised. It now specifies, for each of the ten countries, the source survey, the repository from which the microdata can be downloaded, and the registration or access conditions that apply. It also documents the directory structure the scripts expect, indicating where each downloaded survey file should be placed and the order in which scripts should be run to reproduce the analysis end to end. The repository has additionally been archived at Zenodo with a persistent identifier (https://doi.org/10.5281/zenodo.21470669), which is cited in both the Data Availability and Code Availability statements and included in the reference list.
